# A new extinct species of alligator lizard (Squamata: *Elgaria*) and an expanded perspective on the osteology and phylogeny of Gerrhonotinae

**DOI:** 10.1186/s12862-021-01912-8

**Published:** 2021-09-29

**Authors:** Simon G. Scarpetta, David T. Ledesma, Christopher J. Bell

**Affiliations:** 1 Department of Geological Sciences, Jackson School of Geosciences, The University of Texas at Austin, Austin, USA; 2 Department of Integrative Biology, The University of Texas at Austin, Austin, USA

**Keywords:** Gerrhonotinae, Phylogenetics, Morphology, Osteology, *Elgaria*, Fossils, Anza-Borrego, Pliocene, Biogeography

## Abstract

**Background:**

Alligator lizards (Gerrhonotinae) are a well-known group of extant North American lizard. Although many fossils were previously referred to Gerrhonotinae, most of those fossils are isolated and fragmentary cranial elements that could not be placed in a precise phylogenetic context, and only a handful of known fossils are articulated skulls. The fossil record has provided limited information on the biogeography and phylogeny of Gerrhonotinae.

**Results:**

We redescribe a nearly complete articulated fossil skull from the Pliocene sediments of the Anza-Borrego Desert in southern California, and refer the specimen to the alligator lizard genus *Elgaria*. The fossil is a representative of a newly described species, *Elgaria peludoverde*. We created a morphological matrix to assess the phylogeny of alligator lizards and facilitate identifications of fossil gerrhonotines. The matrix contains a considerably expanded taxonomic sample relative to previous morphological studies of gerrhonotines, and we sampled two specimens for many species to partially account for intraspecific variation. Specimen-based phylogenetic analyses of our dataset using Bayesian inference and parsimony inferred that *Elgaria peludoverde* is part of crown *Elgaria*. The new species is potentially related to the extant species *Elgaria kingii* and *Elgaria paucicarinata*, but that relationship was not strongly supported, probably because of extensive variation among *Elgaria*. We explored several alternative biogeographic scenarios implied by the geographic and temporal occurrence of the new species and its potential phylogenetic placements.

**Conclusions:**

*Elgaria peludoverde* is the first described extinct species of *Elgaria* and provides new information on the biogeographic history and diversification of *Elgaria*. Our research expands the understanding of phylogenetic relationships and biogeography of alligator lizards and strengthens the foundation of future investigations. The osteological data and phylogenetic matrix that we provided will be critical for future efforts to place fossil gerrhonotines. Despite limited intraspecific sampled sizes, we encountered substantial variation among gerrhonotines, demonstrating the value of exploring patterns of variation for morphological phylogenetics and for the phylogenetic placement of fossils. Future osteological investigations on the species we examined and on species we did not examine will continue to augment our knowledge of patterns of variation in alligator lizards and aid in phylogenetics and fossil placement.

**Supplementary Information:**

The online version contains supplementary material available at 10.1186/s12862-021-01912-8.

## Background

Alligator lizards (Squamata: Gerrhonotinae) compose a widely distributed group in North America with a relatively ancient history. There are approximately 60 extant species and a handful of known extinct alligator lizard taxa [1–3]. Extant gerrhonotines are found in southeastern Canada, across the western United States, throughout Mexico, and in Central America south to Panama [4–6]. Gerrhonotines inhabit a wide range of habitats including cloud-forests, temperate pine-oak forests, grasslands, and desert refugia [7–9]. Some extant taxa are habitat generalists, including several species of *Elgaria* [6], but others exhibit specialized ecologies, including arboreal species of *Abronia* [8, 10].

The cranial osteology of gerrhonotine lizards was examined by several researchers [11–16] and gerrhonotines were incorporated into a number of phylogenetic analyses of morphological data [17–21]. Some researchers previously noted difficulty in differentiating gerrhonotine genera based upon osteological morphology [11]. Most previous investigations into the osteology of gerrhonotines focused on identifying osteological features that would serve to distinguish the various genera. Those studies were based on limited sample sizes with relatively low taxon sampling. More recently, patterns of intra- and interspecific variation were documented in *Elgaria* and *Gerrhonotus* to increase our understanding of osteological variation in the skulls of extant gerrhonotines and to explore the potential consequences of that variation for phylogenetic analysis of osteological data [13, 14], with the goal of facilitating identifications of fossil gerrhonotines. Variation has also been documented in the external morphology of gerrhonotines [22].

The known fossil record of total clade Gerrhonotinae extends to the early Eocene [23]. Crown gerrhonotines are documented from the Miocene [24], and divergence-time analyses indicate a middle Oligocene [25], late-middle Eocene [5], or late Eocene to early Oligocene [26] origin for the crown clade. Although many Late Cretaceous and Cenozoic lizard fossils, mostly disarticulated and fragmented cranial elements, were referred to Gerrhonotinae [1, 23, 24, 27–37], most fossils described during the twentieth century lacked known apomorphies of Gerrhonotinae [38].

**Fig. 1:**
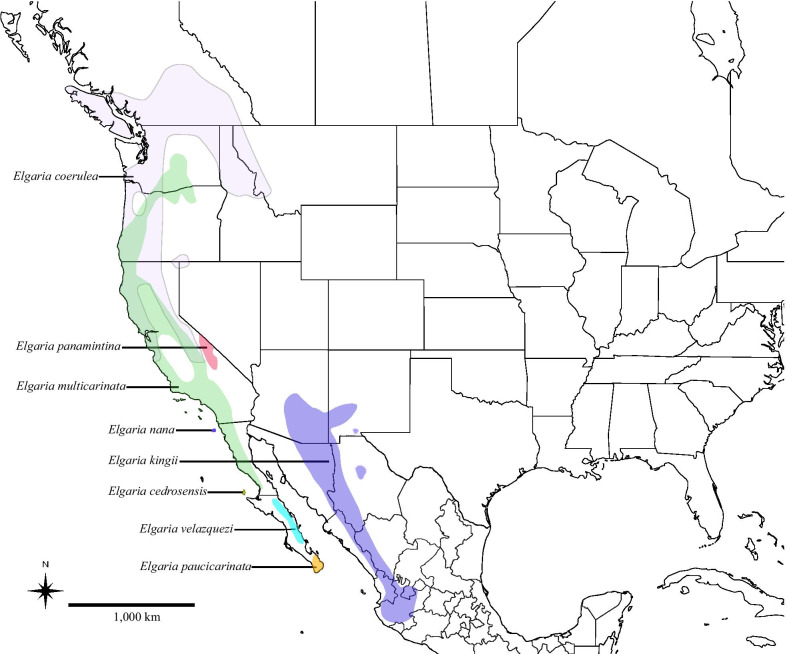
Geographic range distributions of modern species of *Elgaria*

We know of only three formally described articulated fossil skulls referred to Gerrhonotinae or placed via phylogenetic analysis in that clade [1, 12, 17, 33, 39]. One of those skulls, LACM (Natural History Museum of Los Angeles County, formerly the Los Angeles County Museum) 10601, is a well-preserved fossil from Pliocene sediments of the Anza-Borrego Desert of southern California [33]. Although many cranial elements are visible on LACM 10601, most of the previously established apomorphies of gerrhonotines [4] were reported to be obscured on the physical specimen, preventing a refined identification [33]. The fossil was found in an area that is currently inhabited by species of the genus *Elgaria*, which are found in Canada, the United States, and Mexico (Fig. 1) [5, 6], although that alone does not preclude its assignment to any of the other gerrhonotine genera [40]. LACM 10601 was more recently examined by Ledesma [41]; he provided a new description of the fossil using x-ray computed tomography, and investigated the evolutionary relationships of the fossil with phylogenetic analyses that included all species of *Elgaria* and several species of *Gerrhonotus*. Those analyses indicated that LACM 10601 was referable to *Elgaria*, and that the fossil may have a close relationship with species of *Elgaria* that are endemic to the Baja California Peninsula [41]. Recent molecular phylogenies indicate that extant species of *Elgaria* that are today endemic to Baja California are not all each other’s closest relatives. *Elgaria cedrosensis* and *E*. *velazquezi* are hypothesized to be sister taxa, but *E*. *paucicarinata* is hypothesized to be sister to the mainland taxon *E*. *kingii*, which currently inhabits the Sierra Madre Occidental in western Mexico and parts of the southwestern United States [5, 6].

Here, we expand upon the work of Ledesma [41]. We provide a redescription and a new diagnosis of LACM 10601 and formally designate it as a representative of a new extinct species of *Elgaria*. We examined the cranial osteology of a significantly expanded sample of gerrhonotine taxa, which includes many extant gerrhonotine species and clades. We created a phylogenetic character matrix that includes revised characters from the literature and characters that are new to the literature, and which is designed specifically for investigating phylogenetic relationships of Gerrhonotinae and for identifying fossil alligator lizards. In that process, we identified some characters that we excluded from phylogenetic analyses of Gerrhonotinae. Additionally, we provide new morphological data that may help inform the relationships of several systematically enigmatic gerrhonotines (e.g., *G*. *lugoi*, *G*. *parvus*). Using automated binning, we discretized several continuous morphological features into discrete character states. We conducted phylogenetic analyses to identify the fossil, informed by our novel dataset, our past studies of variation in gerrhonotine lizards [13, 14], and published molecular phylogenies [5, 10, 26].

## Collection and original description of specimen

LACM 10601 was collected by Harley Garbani sometime between 1960 and 1965 (L. Murray pers. comm.) and was first described by Norell [33]. The fossil was collected from the Olla Formation (sensu Cassiliano [42]) of the Palm Spring Group, at locality LACM 6552.

## Geological setting

The Palm Spring Group preserves Pleistocene and Pliocene sediments and fossils. The group is known for containing Pliocene petrified wood [43] and Pliocene and Pleistocene vertebrate fossils [44–47]. The mammals of the Palm Spring Group are particularly well-documented (e.g., Opdyke et al. [47]), but many lizards were described by Norell [33], including cnemidophorine teiids, *Xantusia*, skinks, and several pleurodontan iguanians, including sceloporines, *Phrynosoma*, *Dipsosaurus*, and the extinct species *Gambelia corona* (Crotaphytidae) and *Pumilia novaceki* (Iguanidae).

The Palm Spring Group is overlain by the Canebrake Formation, and overlies the marine Imperial Formation. Marine intrusions are known in several members of the Palm Spring Group [48]. The Palm Spring Group contains siltstone, claystone, and conglomerate of various colors and grain sizes, and across the formation the depositional environments include a floodplain, channel, lacustrine environment, or a tidal flat [49]. The Olla Formation is interpreted as an alluvial fan and stream deposit, and also contains deposits from migrating channels of the ancient Colorado River delta [49, 50]. The Olla Formation contains fine-grained gray to olive sandstone, mica-bearing siltstone, claystone, and sandstone conglomerate [48].

## Temporal constraint of locality and a minimum age for crown *Elgaria*

**Fig. 2:**
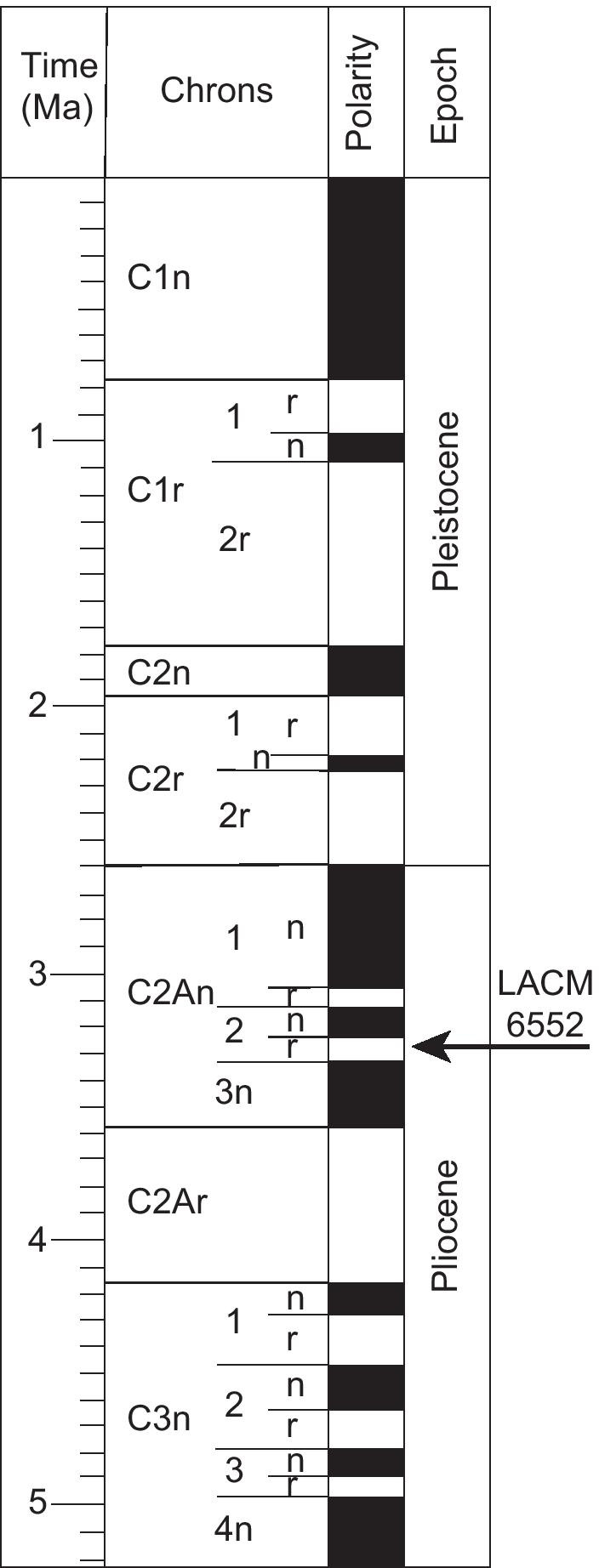
Late Neogene and early Pleistocene temporal framework showing correlation of known chrons with absolute time

LACM 10601 was recovered from the locality LACM 6552 in the Vallecito Creek Area of Anza-Borrego Desert State Park. The locality is in a magnetozone correlated with chron 2An.2r [47, 51] (and L. Murray pers. comm.). Chron 2An.2r spans an age range of 3.33–3.22 Ma (Fig. 2) [52], so we assign the fossil a minimum age of 3.22 Ma. We found LACM 10601 and the new species it represents to be part of crown *Elgaria* (see “Results”), so 3.22 Ma can be used in the future as a minimum age for crown *Elgaria* in divergence time analyses.

## Results

### Systematic paleontology

Squamata Oppel, 1811 [53]

Anguimorpha Fürbringer, 1900 [54]

Anguidae Gray, 1825 [55]

Gerrhonotinae Tihen, 1949 [16]

*Elgaria* Gray, 1838 [56]

*Elgaria peludoverde* sp. nov.

### Holotype

The holotype, LACM 10601, is a nearly complete and articulated fossil skull. The specimen is reposited in the paleontology collections of the Natural History Museum of Los Angeles County (LACM).

### Etymology

Named in honor of Harry W. Greene, an extraordinary educator, author, and tireless promoter of a greater appreciation of natural history, who has cultivated in his career a deep personal appreciation of the fossil record and of alligator lizards. “Peludo Verde” was the name he took while working in Costa Rica; from the Spanish, peludo (‘hairy’) and verde (‘green’).

### Diagnosis

**Fig. 3:**
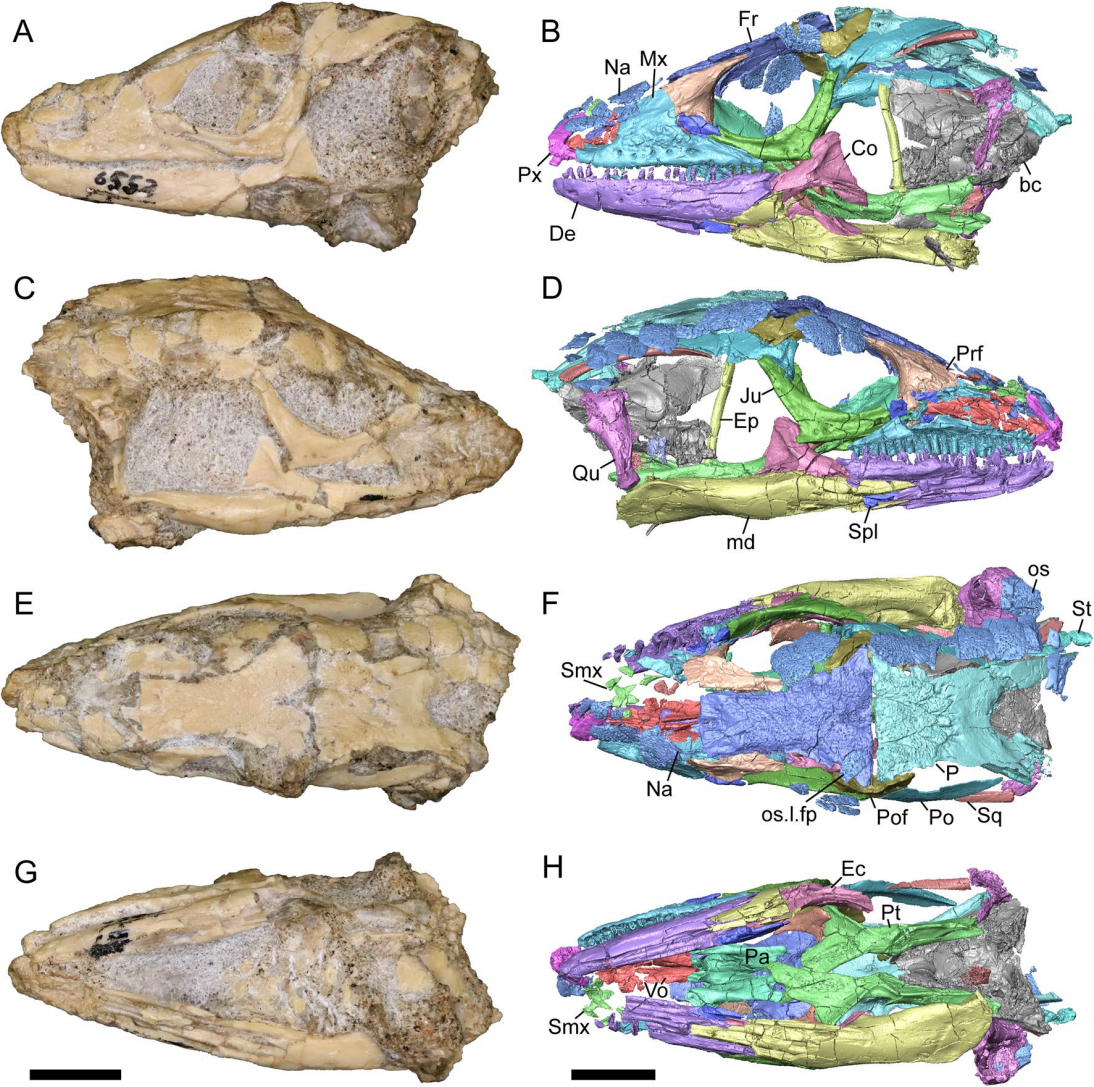
Holotype of *E. peludoverde* (LACM 10601). Images on the left column are of the physical specimen, and those on the right column are surface renderings of the segmented fossil. Scale bars = 5 mm. **a**, **b** Skull in left lateral view. **c**, **d** Skull in right lateral view. **e**, **f** Skull in dorsal view. **g**, **h** Skull in ventral view. *bc* braincase, *Co* coronoid, *De* dentary, *Ec* ectopterygoid, *Ep* epipterygoid, *Fr* Frontal, *Ju* jugal, *md* mandible, *Mx* maxilla, *os* osteoderm, *os.l.fp* osteoderm overlying the frontoparietal shield, *Na* nasal, *P* parietal, *Pa* palatine, *Po* postorbital, *Pof* postfrontal, *Prf* prefrontal, Pt pterygoid, *Px* premaxilla, *Qu* quadrate, *Smx* septomaxilla, *Spl* splenial, *St* supratemporal, *Sq* squamosal, *Vo* vomer

**Fig. 4:**
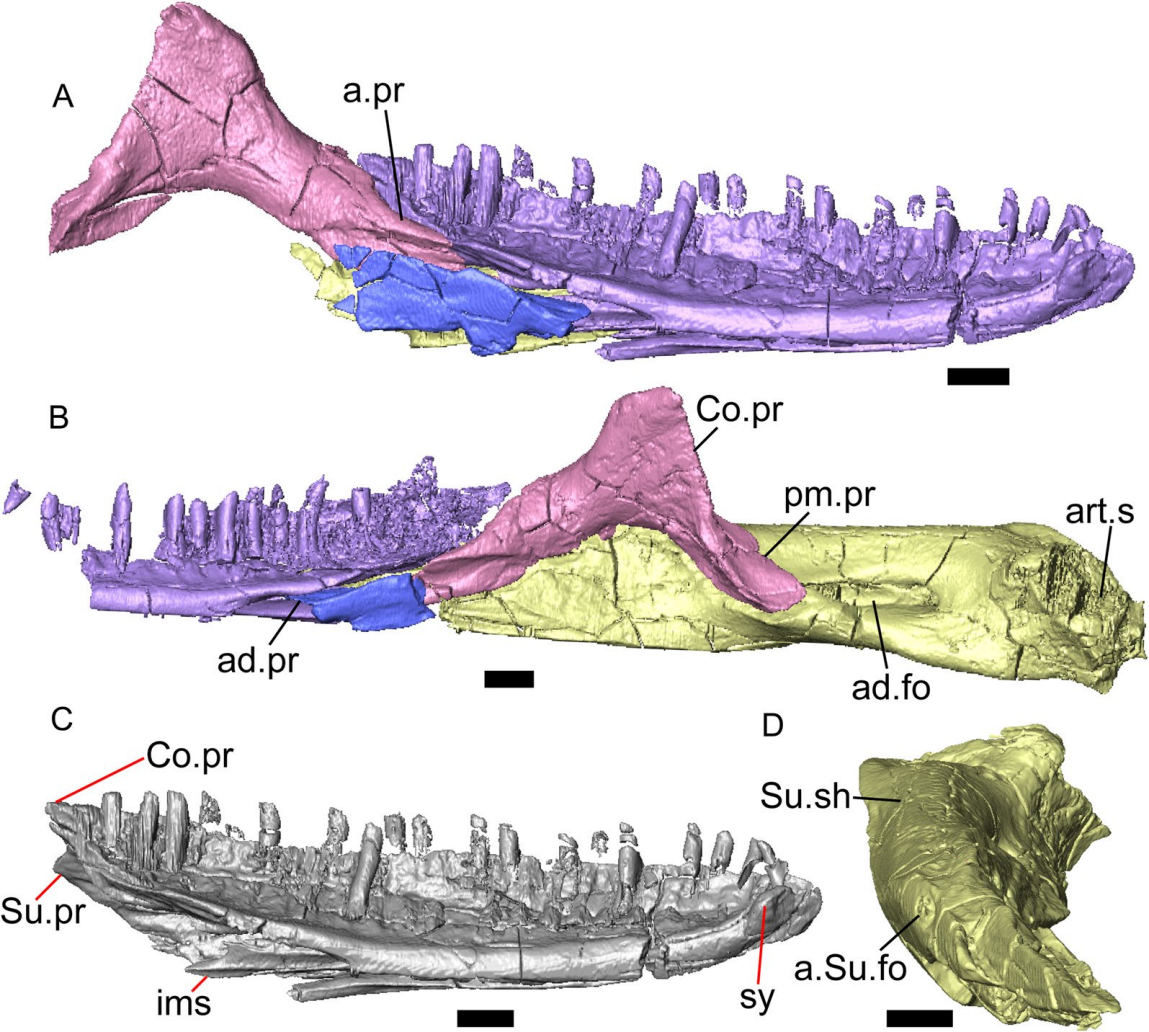
Mandibular elements of LACM 10601. Scale bars = 1 mm. **a** Left mandible in medial view. **b** Right mandible in medial view. **c** Left dentary in medial view. **d** Right surangular in anterior view. *a.pr* anterior process, *a.Su.fo* anterior surangular foramen, *ad.fo* adductor fossa, *ad.pr* anterodorsal process, *art.s* articular surface, *Co.pr* coronoid process (of either the coronoid or the dentary), *ims* intramandibular septum, *pm.pr* posteromedial process, *Su.pr* surangular process, *Su.sh* surangular shelf, *sy* symphysis

**Fig. 5:**
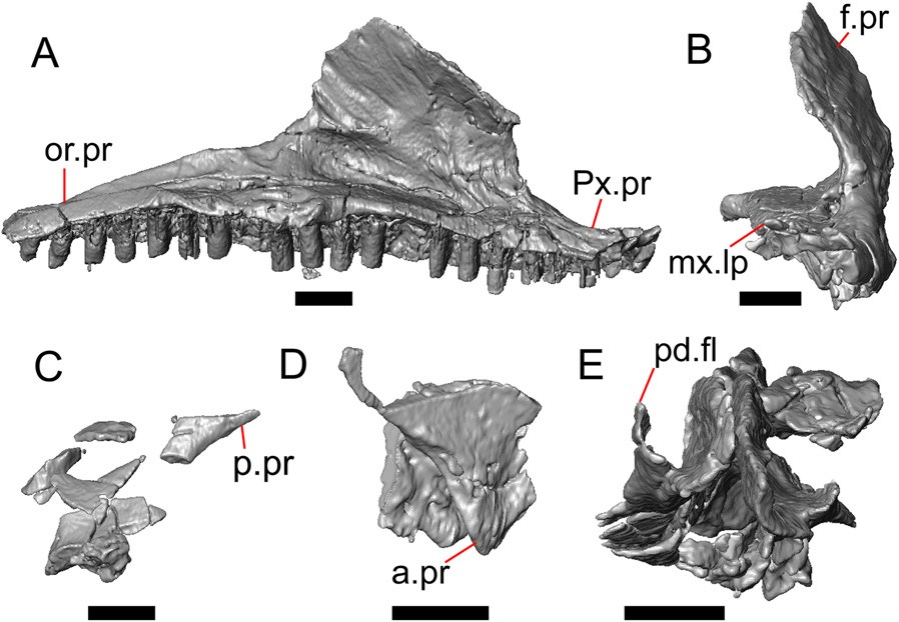
Select cranial elements of LACM 10601. Scale bars = 1 mm. **a** Left maxilla in medial view. **b** Left maxilla in anterior view. **c** Septomaxilla fragments. **d** Left nasal fragment in ventral view. **e** Vomers in posterior view. *a.pr* anterior process, *f.pr* facial process, *mx.lp* maxillary lappet, *or.pr* orbital process, *p.pr* posterior process, *pd.fl* posterodorsal flange, *Px.pr* premaxillary process

LACM 10601 (Fig. 3) is referred to Squamata based on the presence of pleurodont tooth implantation, a single premaxilla, and a splenial, as well as the absence of a quadratojugal and vomerine teeth [19, 57, 58]. We note that not all of those character states are present in all squamates (e.g., *Pseudopus* can have vomerine teeth and some skinks have a paired premaxilla) [19]. The fossil is a member of Anguimorpha given the presence of an open and ventrally-facing Meckel’s canal, non-compound cephalic osteoderms, and an anterior inferior alveolar foramen that is located between the splenial and the dentary (Figs. 3, 4) [28, 59]. The specimen is referred to Anguidae because it possesses laterally imbricating osteoderms, a free posteroventral margin of the intramandibular septum (although the free posteroventral margin is absent in extant anguines) [28, 38], a maxillary lapet, and a squamosal that is not mediolaterally expanded (Figs. 3a–f, 4, 5a, b) [60].

The anguid clades Diploglossinae and Anguinae possess distinct palatal processes of the premaxilla, which are absent in LACM 10601. LACM 10601 has a fused frontal (Fig. 3e–f), which is an apomorphy of Gerrhonotinae among extant Anguidae (including Gerrhonotinae, Anniellinae, Diploglossinae, and Anguinae) [28, 38]. A fused frontal is present in some taxa in the extinct anguid clade Glyptosaurinae, but that clade is unique among anguids in possessing tubercular-textured osteoderms [28], which are absent in LACM 10601. The frontals are also fused in the extinct European anguine *Pseudopus laurillardi* [61], although a suture line is still evident in that species and the frontal tapers uniformly in width anteriorly. LACM 10601 is referable to Gerrhonotinae.

**Fig. 6:**
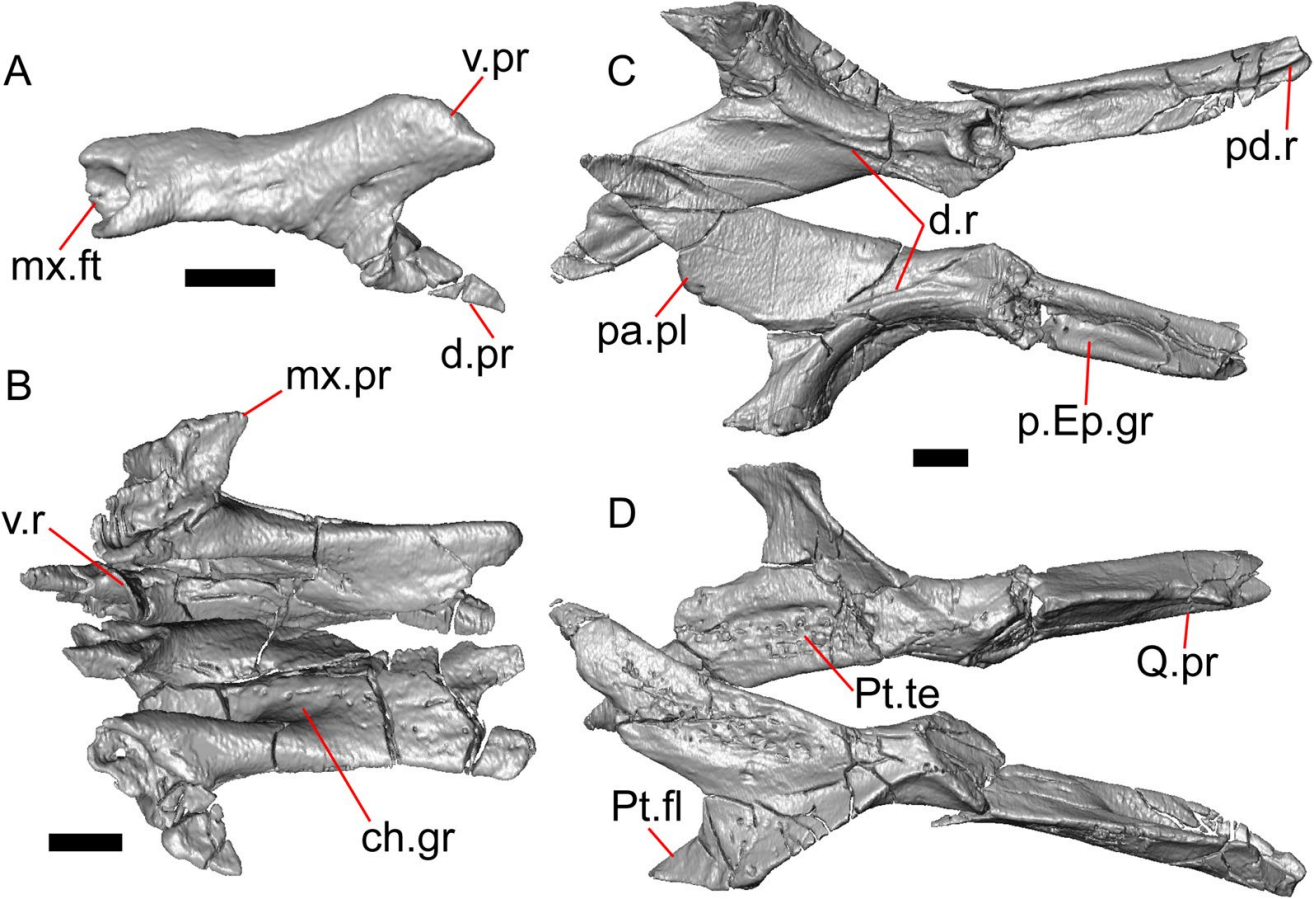
Palatal elements of LACM 10601. Scale bars = 1 mm. **a** Left ectopterygoid in ventral view. **b** Palatines in ventral view. **c** Pterygoids in dorsal view. **d** Pterygoids in ventral view. *ch.gr* choanal groove, *d.pr* dorsal process, *d.r* dorsal ridge, *mx.ft* maxillary facet, *mx.pr* maxillary process, *p.Ep.gr* postepipterygoid groove, *pa.pl* palatal plate, *pd.r* posterodorsal ridge, *Pr.fl* pterygoid flange, *Pt.te* pterygoid teeth, *Q.pr* quadrate process, *v.pr* ventral process, *v.r* ventral ridge

**Fig. 7:**
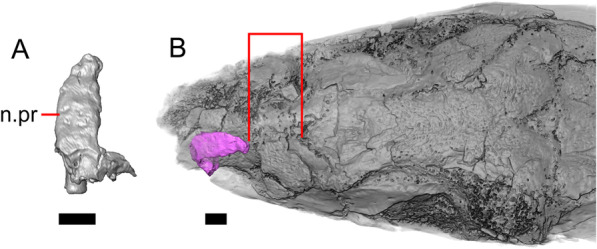
Premaxilla and spatial position of the premaxilla with respect to the frontal of LACM 10601. Scale bars = 1 mm. **a** premaxilla in anterior view. **b** Partially transparent volume rendering of the anterior portion of the skull, and (in magenta) a surface rendering of the premaxilla transformed to be at its natural position (using the transform module in Avizo Lite 9). Red lines mark the anterior margin of the frontal and the posterior margin of the premaxilla. *n.pr* nasal process

LACM 10601 possesses a large patch of pterygoid teeth (Fig. 6), which is present in *Elgaria* and some *Gerrhonotus* (see "Phylogenetic character list" in "Materials and methods"). The fossil lacks vermiculate and heavily sculptured osteoderms, which are characteristic of many species of *Abronia*, and also lacks contact of the premaxilla and the frontal, contact of the maxilla and the frontal, and an ossified bridge between the nasal process and the body of the maxilla (Figs. 3, 7); those features are present in many species of *Gerrhonotus*, *Barisia*, and *Abronia* ("Phylogenetic character list" in "Materials and methods"). The above evidence suggests that the fossil is part of total clade *Elgaria*. We evaluated the relationships of LACM 10601 via phylogenetic analysis of a morphological character matrix that we created, and we ultimately referred the fossil to the crown clade of *Elgaria*. The new taxon is diagnosed from other *Elgaria* in possessing the following unique combination of character states: a supralabial groove on the maxilla (not used as a phylogenetic character), a ventral lamina on the lateral surface of the orbital process of the jugal, a distinct surangular process of the dentary, and a ridge on the dorsal surface of the pterygoid (on the pterygoid flange anterior to the fossa columella).

#### Remarks

LACM 10601 was previously assigned to Gerrhonotinae based on the presence of an anterior process of the surangular, possession of long anterior and posterior maxillary processes, elongate and thin supratemporals, a fused frontal, and the fusion of the prearticular, articular, and surangular [33]. LACM 10601 was reported to resemble *Abronia* in having a supralabial groove on the maxilla [33]. We agree that there is a groove on the posterior portion of the maxilla, and that this feature seems to be unique in the fossil with respect to other *Elgaria*. However, we did not observe that groove in any of the *Abronia* that we examined. It was also suggested that LACM 10601 resembled *Abronia* in the reduction of the lateral concave surface of the quadrate [33]. We do not find that there is a reduction of the lateral concave surface of the quadrate of LACM 10601. Another reportedly derived feature of the fossil was a lacrimal and jugal everted at the maxillary suture [33], although the systematic meaning of that character was not specified. It was previously reported that eversion of the jugal and lacrimal only occurred in *Elgaria*, *Gerrhonotus*, and *Barisia* [12]. A relationship between LACM 10601 and *Abronia* was contradicted by the lack of a sublabial groove of the dentary on the fossil [33]. We agree that the ‘sublabial’ groove is absent on the fossil, and found the groove in some species of *Abronia* and *Barisia* (see Phylogenetic Character List). We interpret LACM 10601 as a mature individual because of the well-developed osteodermal crust on the parietal, constriction of the parietal table, relatively long paroccipital processes, and relatively long supraoccipital in dorsal view [14, 62].

### Description

#### Premaxilla

The right portion of the premaxilla is missing, and the nasal process is laterally curved to the left side due to taphonomic deformation (Figs. 3, 7). The premaxilla is rotated to the left so that it faces anterolaterally. As a result, contact of the premaxilla with adjacent bones was difficult to determine. We inferred the spatial relationship of the nasal process relative to the frontal by rotating the premaxilla in Avizo Lite so that the left maxillary facet on the lateral edge of the palatal shelf was aligned with the corresponding articulation surface of the intact maxilla, and determined that contact with the frontal could not have been present (Fig. 7). That observation is supported by the lack of a posterior extension of the ventral keel of the nasal process of the premaxilla (see "Phylogenetic character list" in "Materials and methods”).

The nasal process is not especially wide or narrow (Fig. 7a). The anterior surface of the nasal process is flat, and the process tapers in width posteriorly. The incisive process projects anteroventrally below the palatal process, and has a midline groove that gives the process a concave appearance ventrally. The preserved left portion of the palatal process preserves the medial ethmoidal foramen just lateral to the base of the nasal process. The foramen likely transmits the ophthalmic branch of cranial nerve five [63]. Some of the anterior face of the premaxilla is sheared away. An anterior midline foramen is evident ventral to the nasal process, but it is not clear whether the foramen would be open anteriorly because of the missing anterior portion of the premaxilla.

Most of the teeth are missing. There is one partial tooth and four or potentially five total tooth positions.

#### Septomaxilla

The right septomaxilla is preserved, but is broken into a few pieces (Figs. 3f, 5c). On one of the pieces, a foramen penetrates the bone horizontally. This piece has a flattened medial surface where it would have contacted or closely approached its contralateral element. Another piece of the septomaxilla preserves an anterolateral projection that is also present in many extant gerrhonotines. The posterior process is evident, and is long posteriorly.

#### Nasal

The anterior process and premaxillary facet of the left nasal are preserved. The anterior process is well-developed (Fig. 5d). An indistinct piece of bone on the right side of the skull may be a piece of the right nasal.

#### Maxilla

The left maxilla is mostly preserved, but only the orbital process of the right maxilla is present. The orbital process is long relative to the premaxillary process of the left maxilla. The facial process is broad (Figs. 3a, b, 5a), and is inflected medially (Fig. 5b). The anterior edge of the facial process possesses a small anterior spur. The premaxillary process is bifurcated with a medial maxillary lappet that is short and fragmented. The anterolateral portion of the premaxillary process is also short and is located above a distinct facet for the lateral edge of the palatal process of the premaxilla. The crista transversalis connects with the anteromedial surface of the facial process and extends anteriorly towards the base of the premaxillary process. There is a distinct recess at the base of the medial surface of the facial process (medial recess for the nasal sac) just posterior to the crista transversalis. The superior alveolar foramen and/or maxillary trigeminal foramen [64] emerge from a groove on the dorsal surface of the palatal shelf.

The palatine process is a distinct and substantial medial projection from the palatal shelf, and is thickened relative to the rest of the shelf (Fig. 5a, b). There is a small but deep articulation surface on the palatine process for the palatine.

There are five nutrient foramina on the lateral surface of the maxilla and the posteriormost foramen is noticeably larger (Fig. 3a, b). Smaller foramina are located more dorsally on the anterior half of the facial process. Posterior to the nutrient foramina, there is a shallow anterior–posterior depression along the anterior portion of the orbital process. The ventrolateral part of the facial process is covered with sculpturing, particularly the ventral part of the process. There are 20 tooth positions on the left maxilla.

#### Lacrimal

**Fig. 8:**
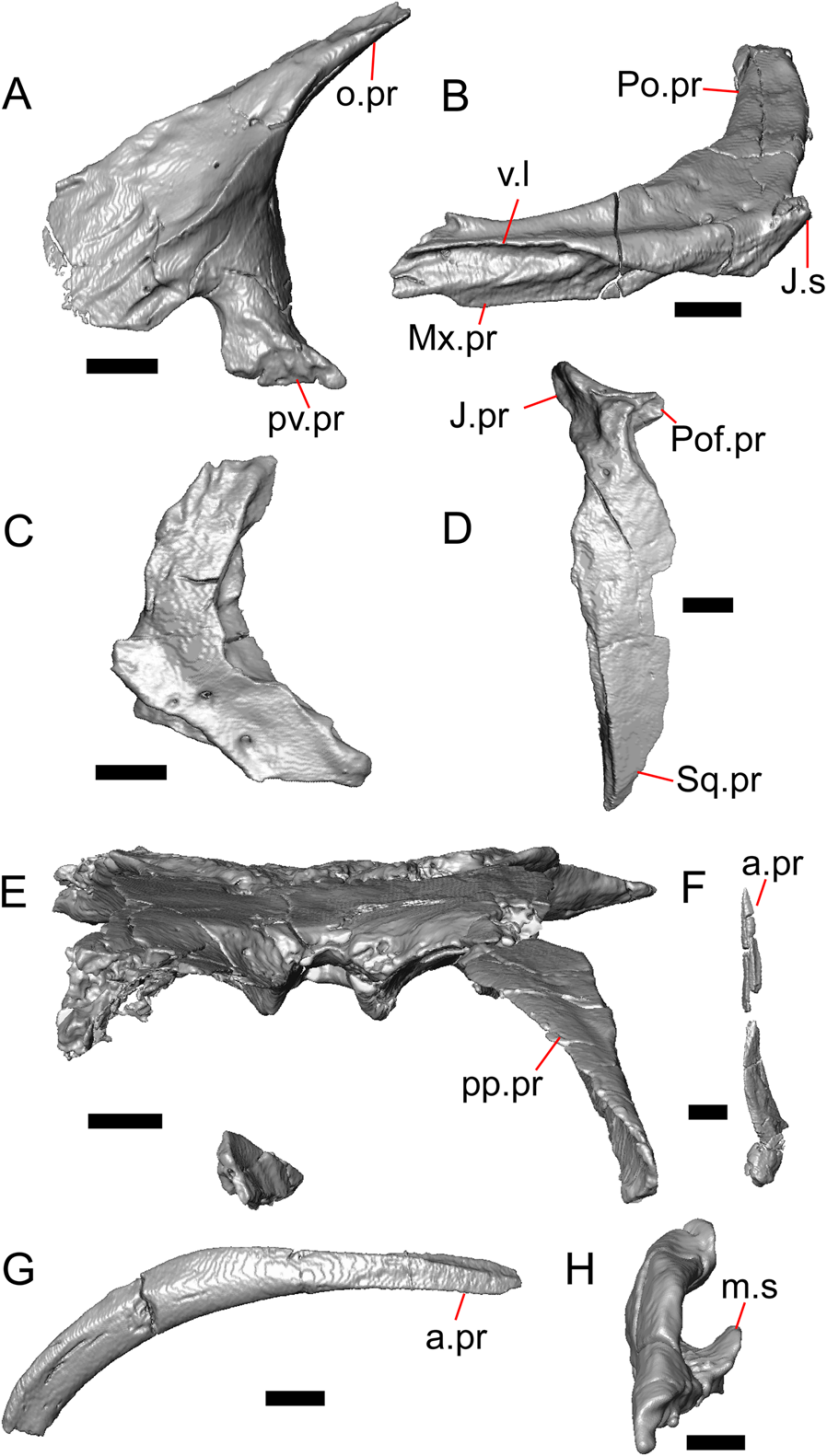
Select cranial elements of LACM 10601. Scale bars = 1 mm except for **h**, for which the scale bar = 0.5 mm. **a** Left prefrontal in lateral view. **b** Left jugal in ventrolateral view. **c** Left postfrontal in dorsal view. **d** Left postorbital in dorsal view. **e** Parietal in posterior view. **f** Right supratemporal in dorsal view. **g** Right squamosal in lateral view. **h** Left lacrimal in posterior view. *a.pr* anterior process (of either the supratemporal or the squamosal), *J.pr* jugal process, *J.s* jugal spur, *m.s* medial shelf, *Mx.pr* maxillary process, *o.pr* orbital process, *Po.pr* postorbital process, *pp.pr* postparietal process, *Pof.pr* postfrontal process, *pv.pr* posteroventral process, *Sq.pr* squamosal process, *v.l* ventral lamina

Both lacrimals are present, but only the left lacrimal is well-preserved (Fig. 3a, b). The posterior end of the bone is elongate and forms a posterior process that articulates with a facet at the anterior end of the jugal. The lacrimal is excavated medially and has a projection extending dorsally from the medial shelf which forms part of the medial border of the lacrimal foramen (Fig. 8h). The lacrimal lacks lateral sculpturing (Fig. 3b).

#### Prefrontal

The main body of the prefrontal is broad with a rounded anterior edge. The anterior half of the lateral surface of the main body is defined by an articulation surface for the facial process of the maxilla (Fig. 8a). There is a distinct ridge posterior to the articulation facet for the facial process that extends posterodorsally onto the ventral edge of the orbital process of the prefrontal. The orbital process articulates with the lateral surface of the frontal (Fig. 3b, d). The medial surface of the orbital process has a defined groove that continues anteriorly onto the main body of the prefrontal. The posteroventral process extends posteriorly to contact the jugal and contribute to the border of the lacrimal foramen. There are two foramina on the lateral surface of the prefrontal just dorsal to the lateral ridge.

#### Jugal

The anterior and posteriormost portions of the right jugal are highly fragmented or missing, but both jugals are otherwise complete (Fig. 3a, d). The postorbital (temporal) process has a posteromedial articulation facet for the postorbital. The jugal is broadened at the ventral inflection point in lateral view. Anterior to the inflection point, the maxillary (orbital) process narrows abruptly. The postorbital process narrows more gradually. There is a distinct ventral lamina on the orbital process to articulate with the orbital process of the maxilla (Fig. 8b). A jugal spur is present, as are two lateral foramina and one medial foramen near the inflection point.

#### Frontal

**Fig. 9:**
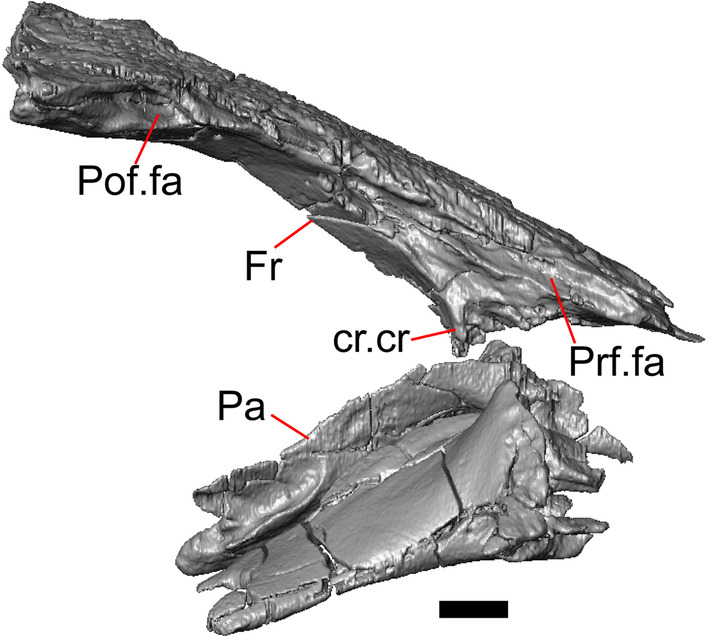
Frontal and palatines of LACM 10601 in right lateral view. Scale bar = 1 mm. *cr.cr* crista cranii, *Fr* frontal, *Pa* palatine, *Pof.fa* postfrontal facet, *Prf.fa* prefrontal facet

The frontal is a single midline element (Fig. 3e, f). The dorsal surface is moderately sculptured with a pitted and somewhat undulose texture. The bone widens posteriorly to meet the parietal, and there is a slight constriction between the orbits. The anteriormost tip of the bone is missing, but some part of all three anterior processes are preserved as are portions of the nasal facets, suggesting that only a small portion of the anterior tip is missing. Due to the shifting of the palatines within the skull, it is not possible to determine whether the crista cranii project below the dorsal surface of the palatine (Fig. 9). There is an anterolateral facet that articulates with the prefrontal, which has a well-developed ridge that slots into a groove on the prefrontal. The posterolateral edge of the frontal has an articulation facet for the postfrontal (Fig. 9).

#### Parietal

The parietal table is broad, flattened, and roughly trapezoidal (Fig. 3e, f). The anterodorsal surface of the parietal table is sculptured with osteodermal crust. The parietal foramen is present on the anterior sculptured portion of the parietal table. The anterior edge of the bone has a straight linear contact with the frontal. The post-parietal processes converge towards one another anteriorly where they connect with the main body of the bone, but do not contact (Fig. 8e). LACM 10601 lacks recesses on each side of the posterior surface of the parietal table (Fig. 8e). On the ventral surface, there are distinct ventrolateral crests (epipterygoid processes) as well as well-developed ridges that define the lateral edges of the pit for the ascending process of the supraoccipital. Although the bones appear to have shifted in their positions, the epipterygoid processes of the parietal and the left epipterygoid are far from contacting one another. The posterior edge of the parietal between the post-parietal processes has a slight notch.

#### Postfrontal

The lateral edge of the left postfrontal overlaps with the anteromedial portion of the left postorbital. The left postfrontal has a lateral expansion near the inflection point between the anterior and posterior process of the element (Fig. 8c), while the right postfrontal is characterized by a corner at the inflection point. Both postfrontals are rotated ventrally. The medial surface of the postfrontal has a facet for articulation with the frontal and parietal. There are three foramina on the dorsal surface of the posterior process (Fig. 8c).

#### Postorbital

The postorbitals are well-preserved, but are shifted ventrally. The jugal process projects ventrally along the medial surface of the jugal and bears a distinct articulation facet for the jugal. The postfrontal process is somewhat shorter than the jugal process. The postorbital is not markedly widened along its length; however, the left postorbital is missing a portion of the medial edge (Fig. 8d). The postorbital extends posterior to the anterior tip of the supratemporal as well as the posterior edge of the parietal between the post-parietal processes. The right postorbital and supratemporal contact one another, although this may be a result of the fossilization process (Fig. 3f).

#### Squamosal

Most of the right squamosal is well-preserved except for the posterior end of the bone (Fig. 8g). Both the anterior and posterior processes of the squamosal curve ventrolaterally, and the anterior end tapers to a point. The squamosal contacts the lateral surface of the supratemporal and the posterior portion of the postorbital. The posterior end of the squamosal is fragmented and we could not determine whether it curved anteriorly.

#### Supratemporal

Both supratemporals are present, but only the right is well-preserved (Fig. 3f). Posteriorly, the supratemporal broadens mediolaterally and increases in height. Anteriorly, the supratemporal tapers to a single point.

#### Vomer

Both vomers are present, but are fragmented (Fig. 3h). The anterior portion of the left vomer is shifted ventrally. The posterior palatine processes are in close proximity, and possess a facet for the anterior vomerine process of the palatine. There is a well-developed posterodorsal lamina (Fig. 5e). The medial ridge is tall and steeply inclined. On the left vomer there is a ridge that runs anterolaterally from the medial ridge of the vomer. The foramen for the medial palatine nerve appears to be present on the left vomer, but the angle at which the foramen penetrates the bone could not be determined.

#### Palatine

Both palatines are translated and rotated so that they medially overlap one another (Figs. 3h, 6b). The prefrontal blocks contact between the palatine and the jugal on the left side. The lateral maxillary process of the palatine is pointed posterolaterally. The bifurcated posterior process has medial and lateral projections that are short and blunt, with the lateral projection extending slightly farther posteriorly. The palatine has a deep choanal groove. The maxillary process of the left palatine has a concave facet for articulation with the maxilla. Just above that facet, the bone is pierced horizontally by the large infraorbital foramen. On the anterior portion of the dorsal surface of the palatine there is a distinct articulation facet for the prefrontal. The vomerine process of the palatine has a ventral ridge at the posterior margin of the articulation facet for the vomer (Fig. 6b).

#### Ectopterygoid

Both ectopterygoids are well-preserved (Fig. 6a). The anterior end of the bone has a deep concave facet for the orbital process of the maxilla. The posterior end of the bone is bifurcated with dorsal and ventral processes that form a tight articulation with the pterygoid flange (Fig. 6a). Viewed dorsally, the left ectopterygoid has a distinct lateral bulge which is less developed on the right ectopterygoid. There is a foramen on the ventral surface of the ectopterygoid just anterior to the suture line with the pterygoid. On the left ectopterygoid, there is a single foramen within the articulation facet for the transverse flange of the pterygoid, and there are two foramina on the right ectopterygoid.

#### Pterygoid

Both pterygoids are present, but the posteriormost portions of the quadrate processes are not completely preserved especially on the left side (Fig. 6c, d). The anterior portion of the bone has a flattened palatal plate that tapers anteriorly. The anterior end of the palatal plate has a facet on the dorsal surface for the medial posterior palatine process. There is an anterolaterally projecting pterygoid flange that is flattened laterally and curves to slot into the articulation facet of the ectopterygoid. There is a distinct anteroposteriorly directed ridge on the dorsal surface of the pterygoid, anterior to the fossa columella (Fig. 6c). The quadrate process of the pterygoid has a well-defined groove on the dorsal surface that begins just posterior to the fossa columellae, and on the right pterygoid a well-developed posterodorsal ridge is evident (Fig. 6c). A small foramen is visible just posterior to the fossa columella on the left pterygoid. There is a broad patch of blunt teeth and tooth implantation sites on the left pterygoid, and a similar patch of implantation sites on the right pterygoid (Fig. 6d).

#### Quadrate

**Fig. 10:**
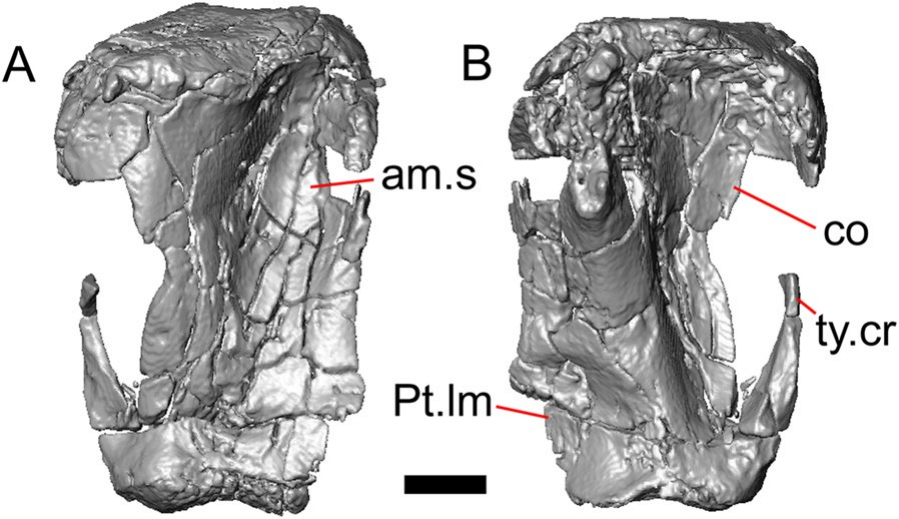
Right quadrate of LACM 10601. Scale bar = 1 mm. **a** Anterior view. **b** Posterior view. *am.s* anteromedial surface, *co* conch, *Pt.lm* pterygoid lamina, *ty.cr* tympanic crest

Both quadrates are present, and the right quadrate is nearly complete (Fig. 10), but the left quadrate is highly fragmentary. The tympanic crest is well-developed but incompletely preserved. The conch is deep (contra Norell [33]) and is substantially wider dorsally than ventrally (Fig. 10b). The cephalic condyle and squamosal notch are both present but portions of each structure are missing. The quadrate column widens near its base. There is a medial expansion of the pterygoid lamina dorsal to the articulation surface for the the quadrate process of the pterygoid. The anterior surface of the quadrate possesses a deep excavation medial to the convex anterior surface of the conch (Fig. 10a).

#### Epipterygoid

The left epipterygoid is preserved (Fig. 3b, d). The epipterygoid articulates in the fossa columella of the pterygoid and possesses a slight lateral groove at its ventral end. Although the epipterygoid does not contact the ventrolateral crest (epipterygoid process) of the parietal, it does contact the alar process of the prootic.

#### Sphenoid

**Fig. 11:**
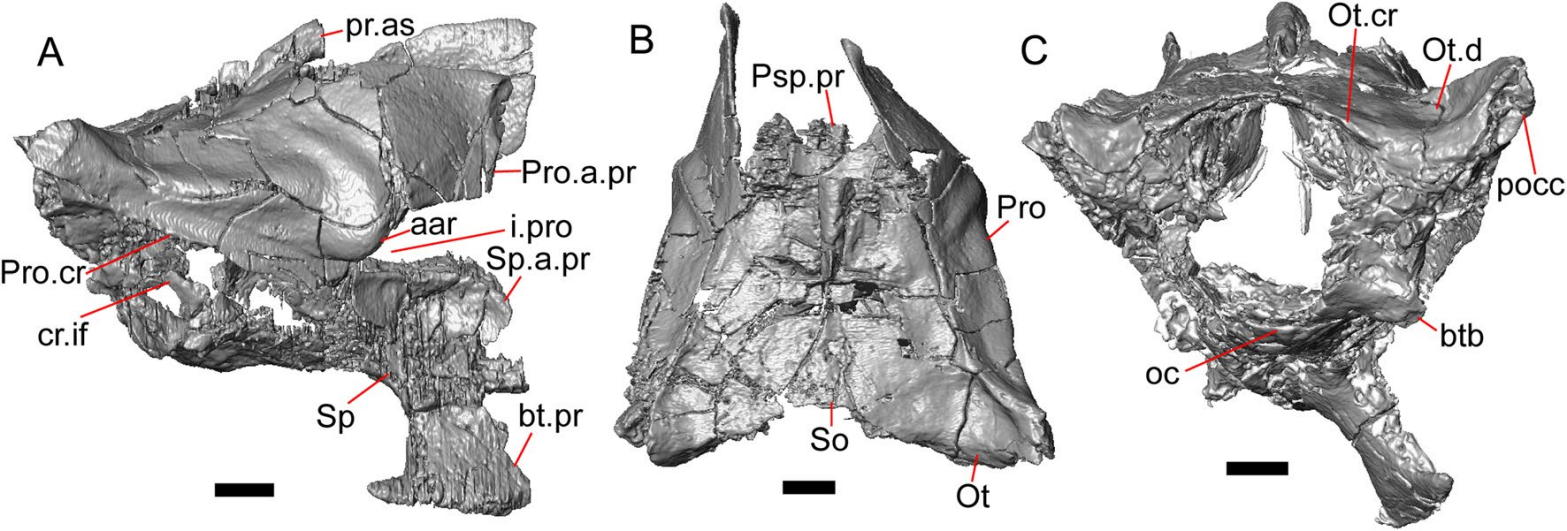
Braincase of LACM 10601. Scale bars = 1 mm. **a** Braincase in right lateral view. **b** Braincase in dorsal view. **c** Braincase in posterior view. *aar* anterior ampullar recess, *bt.pr* basipterygoid process, *btb* basiocciptal tubercle, *cr.if* crista interfenestralis, *i.pro* incisura prootica, *oc* occipital condyle, *Ot* otooccipital, *Ot.cr* otooccipital crest, *Ot.d* otooccipital depression, *pocc* paroccipital process, *pr.as* ascending process, *Pro* prootic, *Pro.a.pr* alar process of the prootic, *Pro.cr* prootic crest, *Psp.pr* parasphenoid process, *So* supraoccipital, *Sp* sphenoid, *Sp.a.pr* alar process of the sphenoid

The sphenoid is fragmented (Fig. 11). The sphenoid contacts the prootic dorsolaterally and the basioccipital posteriorly. There is a shallow depression on the ventral surface in between the basipterygoid processes. The left basipterygoid process is missing, but the right side is well-preserved and projects ventrolaterally from the main body of the bone, and has a prominent posterior spur. The alar process of the sphenoid projects anteriorly and is rounded. Between the alar processes the crista sellaris overhangs the pituitary fossa. Only the base of the parasphenoid process is preserved. A depression (sella turcica) is located between the trabeculae (crista trabecularis of Oelrich [65]), but the depression ends abruptly before reaching the posterior wall of the pituitary fossa. The depression is replaced posteriorly with a sloped surface of the dorsum sella that contains a carotid foramen on either side, which each open anteromedially. Only the right vidian canal is preserved and the anterior opening is located lateral to the trabecula. The posterior opening of the vidian canal is obscured by bone fragments and matrix. The openings for the abducens foramina are not preserved.

#### Supraoccipital

The supraoccipital is well-preserved (Fig. 11b). The suproccipital contacts the prootic anterolaterally and the otooccipital posterolaterally. On the dorsal surface, the ascending process projects anterodorsally and rises at a relatively low angle with respect to the bone. A tall medial crest (sagittal crest of McDowell and Bogert [66]) continues posteriorly on the dorsal surface (Fig. 11a). The base of the ascending process connects to the body of the supraoccipital close to the level of anterior extent of the anterolateral tips of the bone. The paths for the anterior and posterior semicircular canals are filled with matrix of a similar density to the fossil, which can be seen in the CT slices. Those paths become obscured at the osseous common crus where the bone is fragmented. An endolymphatic foramen is located on the medial (external) surface of the supraoccipital portion of the cavum capsularis. The supraoccipital appears to be largely fused to the otooccipital posteriorly.

#### Prootic

The prootics are present but fragmented (Fig. 11a, b). The prootic contacts the supraoccipital posterodorsally, the otooccipital posteriorly, the basioccipital ventrally, and the sphenoid anteroventrally. The alar process is long with a squared end that, on the left side, contacts the epipterygoid. The external surface of the anterior ampullar recess is large and bulbous, forming part of the dorsal border of a narrow incisura prootica. An elongate anterior inferior process that lies on the sphenoid forms the ventral border of the incisura prootica, and has a dorsal margin that is oriented horizontally. A well-developed supratrigeminal process is present, but is obscured in lateral view by the anterior ampullar recess (Fig. 11a). A well-defined crista prootica is preserved on the right prootic. Neither prootic is preserved well enough to distinguish the location of the facial foramen, but the prootic contribution to the anterior border of the fenestra ovalis is somewhat preserved. On the medial surface on the right prootic, the acoustic recess is visible. The medial opening for the facial foramen as well as the openings for the anterior and posterior acoustic foramina are not preserved.

#### Otooccipital

Portions of the otooccipitals are preserved (Fig. 11). The otooccipital contacts the supraoccipital dorsally and dorsomedially, although the exact contact between the bones is unclear due to fusion and fragmentation. The otooccipital contacts the basioccipital ventromedially and the prootic anteriorly. The posterior surface of the otooccipital is fragmented and the vagus and hypoglossal foramina were not identified. Much of the paroccipital processes are missing, but a depression is visible on the dorsal surface where the paroccipital process is continuous with the bone (Fig. 11c). A portion of the crista interfenestralis is preserved, thus preserving part of the otooccipital contribution to the lateral aperture of the recessus scala tympani.

#### Basioccipital

Parts of the left and posterior portions of the basioccipital are missing. The basioccipital contacts the sphenoid anteriorly, the prootic laterally, and the otooccipital posterolaterally. The ventral surface is convex ventrally, and a preserved basal tubercle is oriented ventrolaterally on the right side. The dorsal surface of the basal tubercle constitutes the ventral margin of the lateral aperture for the recessus scala tympani (Fig. 11c). The occipital condyle is incomplete.

#### Dentary

Portions of both dentaries are preserved, but much of the right dentary is missing (Fig. 3a–d). The dentary has a short dorsoventral height, and tapers gradually in height posterior to the ramus. The Meckelian groove is open, and faces medially along its posterior portion and ventromedially along its anterior portion. There is a well-developed, free posteroventral projection of the intramandibular septum (Fig. 4c).

The posteroventral portion of the left dentary is fragmented and so the development of the angular process was not possible to ascertain. The coronoid process is a pronounced triangular projection that articulates with the anterodorsal surface of the anterolateral process of the coronoid. There is also a discrete surangular process that is separate from the coronoid process (Fig. 4c). There are five or six nutrient foramina on the lateral surface of the bone. There are 24 tooth positions visible on the left dentary (Fig. 4a).

#### Coronoid

Both coronoids are well-preserved. The posteromedial process of the coronoid slants posteroventrally and terminates just anterior to the adductor fossa (Fig. 4b). The coronoid (dorsal) process is long in lateral view and possesses a ridge on the lateral surface anterior to a depression where adductor muscles would attach. The lateral process (labial process of Evans [63]) is a small projection that, together with the anteromedial process, creates a notch in which the surangular process of the dentary articulates. The anteromedial process extends below the dental shelf and reaches anteriorly past the third most distal tooth position of the dentary (Fig. 4a). Midway along this process there is a foramen that penetrates ventrally. The right coronoid does not contribute to the border of the anterior surangular foramen (Fig. 3c-d).

#### Surangular and articular

The right surangular and articular are well-preserved, but the left elements are largely absent besides some anterior fragments. The surangular and the articular (including the prearticular) are indistinguishable in CT slices and segmented surfaces. The lateral surface of the surangular has a crest that runs anteroventrally. The dorsal surface of the surangular is raised and is sloped on both its lateral and medial surfaces (Fig. 4d). The surangular possesses an anterior surangular foramen as well as a posterior foramen on the lateral surface under the surangular crest. The adductor fossa faces medially and is deep. The retroarticular processes are absent, but the articular surface for the quadrate is present.

#### Splenial

Fragmented pieces of both splenials are present (Fig. 4a, b). The splenial has a small anterior projection dorsal to the anterior inferior alveolar foramen. The left splenial has a preserved posterodorsal process that articulates with a facet on the lateral surface of the anteromedial process of the coronoid.

#### Dentition

Teeth and tooth crowns in particular are absent or poorly preserved due to over-preparation prior to the original examination of the specimen by Norell [33]. Those that are present on the dentary are ‘chisel-shaped’ (see Gauthier [28]) to nearly bicuspid, in that the distal portion of the crown attains a higher dorsal or ventral extent than the mesial portion of the crown (Fig. 4a–c).

#### Osteoderms

The osteoderms laterally imbricate and have a distinct anterior gliding surface (Fig. 4a–f). Osteoderms become more rectangular and regular in shape posteriorly. The osteoderms are moderately sculptured, and have a dorsal pattern of scattered lines with some pits. The cranial osteoderms lack keels. Sculpturing on the parietal and frontal is similar to the osteoderms. The frontoparietal shields of the frontal are distinct, and are separated by a triangular interparietal shield. There is an osteoderm that partially covers the left frontoparietal shield (Fig. 3d). On the right side that osteoderm appears to have fallen off, because there is an apparent facet for an osteoderm like the one present on the left side of the frontal. No ventral or lateral osteoderms are preserved.

### Phylogenetic analyses

We employed Bayesian inference and parsimony on our novel dataset to infer phylogenetic relationships among gerrhonotines and ascertain the placement of *Elgaria peludoverde* with respect to extant gerrhonotines. We performed analyses without any topological constraints, and two sets of analyses with scaffolds based on previously published molecular phylogenies (see "Materials and methods”).

### Relationships among extant gerrhonotines

**Fig. 12:**
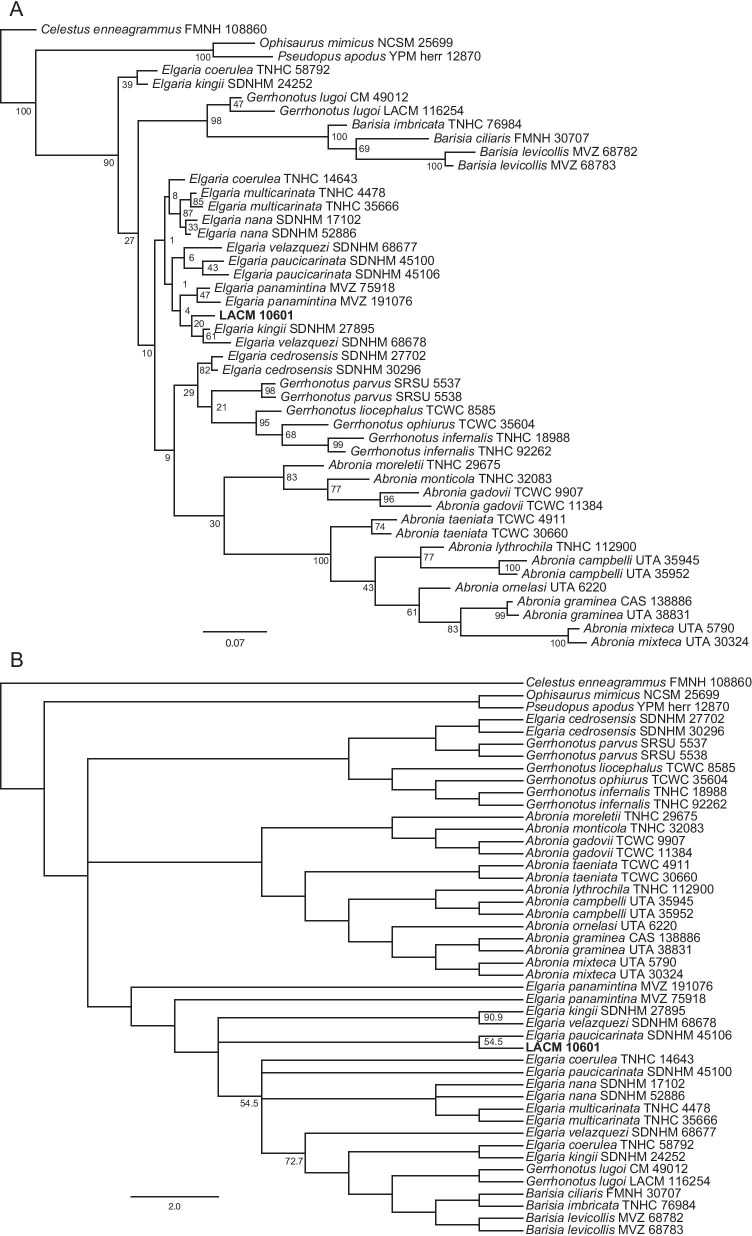
Majority-rule consensus trees from the unconstrained phylogenetic analyses. For the Bayesian analysis, node values are posterior probabilities as a percent. For the parsimony analysis, node values indicate the percent of MPTs in which a given node occurred; if no value is listed, the node occurred in all MPTs. **a** Bayesian analysis. **b** Parsimony analysis

The unconstrained phylogenies (Fig. 12) were similar in many ways to published molecular and morphological phylogenies, although many posterior probability values were low in the Bayesian analyses (Fig. 12a). We inferred monophyly of *Abronia* in all analyses; however, species formerly placed in *Mesaspis* (represented here by *A. gadovii*, *A monticola*, and *A. moreletti*) were sister to the rest of *Abronia* as in previous osteological analyses [12], as opposed to the paraphyletic ‘*Mesaspis*’ inferred by recent phylogenomic analyses [10]. We also found a monophyletic *Barisia* and a monophyletic keeled-scale clade of *Gerrhonotus* (represented here by *G. infernalis*, *G liocephalus*, and *G. ophiurus*) [26]. In the unconstrained Bayesian analysis, the keeled-scale clade of *Gerrhonotus* was sister to *Abronia* (Fig. 12a), but in the parsimony analysis (both the strict and majority-rule consensus trees) there was a polytomy among the major gerrhonotine clades (Fig. 12b). *Gerrhonotus lugoi* was consistently placed as the sister taxon of *Barisia*, a relationship found in some of the analyses by García-Vázquez et al. [26]. This is the first osteological phylogeny to infer a close relationship between *G*. *lugoi* and *Barisia*, and substantiates our previous observations [14]. Unconstrained analyses replicated several other relationships inferred in previous studies. Those include monophyly of species placed in the subclade *Auriculabronia* (represented here by *A. campbelli* and *A. lythrochila*) [8, 10], relationships among the keeled-scale *Gerrhonotus*, a close relationship between *G*. *parvus* and the keeled-scale *Gerrhonotus* ([26]; but see below), and a close relationship between *E*. *multicarinata* and *E*. *nana* [5].

In the unconstrained analyses, conspecific specimens were generally inferred to be sister taxa with high or moderate support, except for among *Elgaria*, in which only *E*. *multicarinata* and *E*. *nana* were consistently placed together (Fig. 12). Additionally, external morphological and phylogenomic studies suggest a close relationship between or a species complex of individuals referred to *A. graminea* and *A. taeniata* [10, 22]. In the unconstrained analyses from this study, however, *A. taeniata* was placed as sister to other *Abronia* (excluding species previously placed in *Mesaspis*).

*Elgaria* was not monophyletic in both unconstrained analyses here (Fig. 12), but monophyly for the genus is strongly supported by molecular data [5, 25]. *Elgaria cedrosensis* was outside *Elgaria* in the unconstrained analyses (see below). Two specimens of *Elgaria* were sister to all other gerrhonotines in the unconstrained Bayesian analysis, and in the parsimony analysis, *Barisia* and *G*. *lugoi* were nested in *Elgaria*. The difficulty we encountered with *Elgaria* aligns with our previous work in which we found substantial and sometimes excessive intra- and interspecific variation in *Elgaria*, including features that were purportedly diagnostic or apomorphic for the clade with respect to other gerrhonotines [14].

**Fig. 13:**
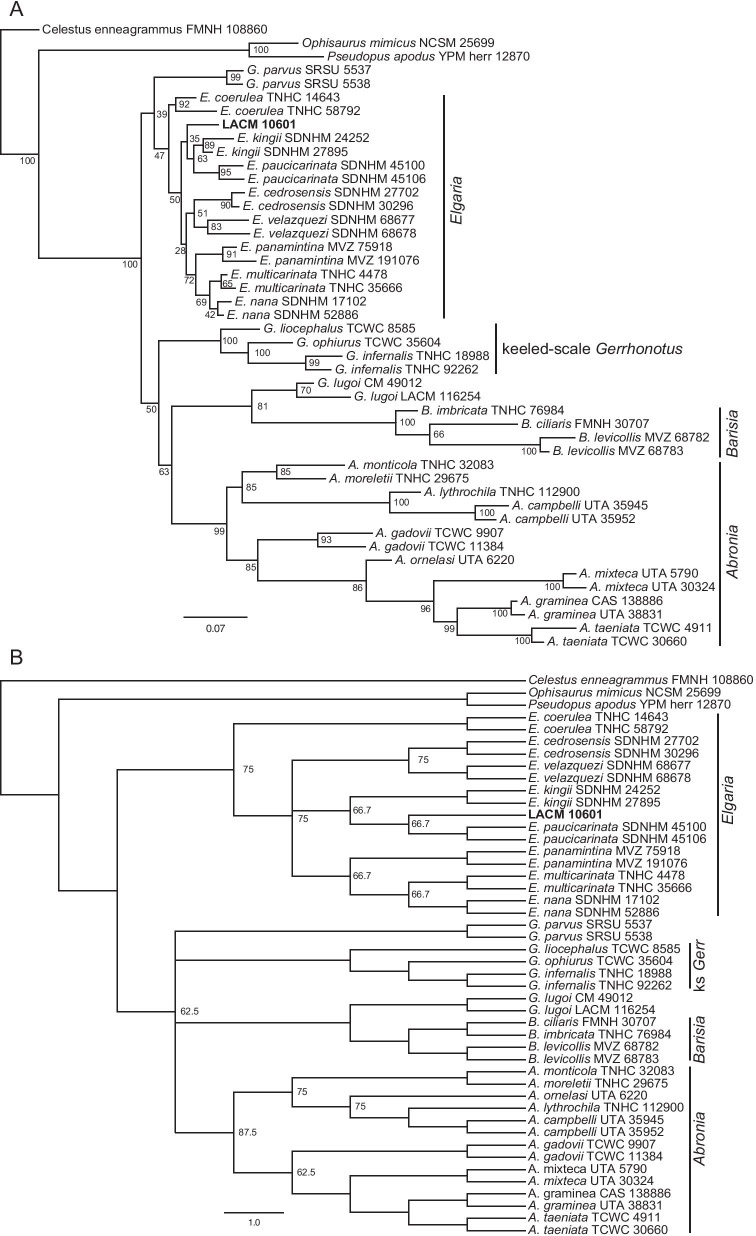
Majority-rule consensus trees from the partially constrained phylogenetic analyses in which *G. parvus* could attach anywhere. For the Bayesian analysis, node values are posterior probabilities as a percent. For the parsimony analysis, node values indicate the percent of MPTs in which a given node occurred; if no value is listed, the node occurred in all MPTs. **a** Bayesian analysis. **b** Parsimony analysis

**Fig. 14:**
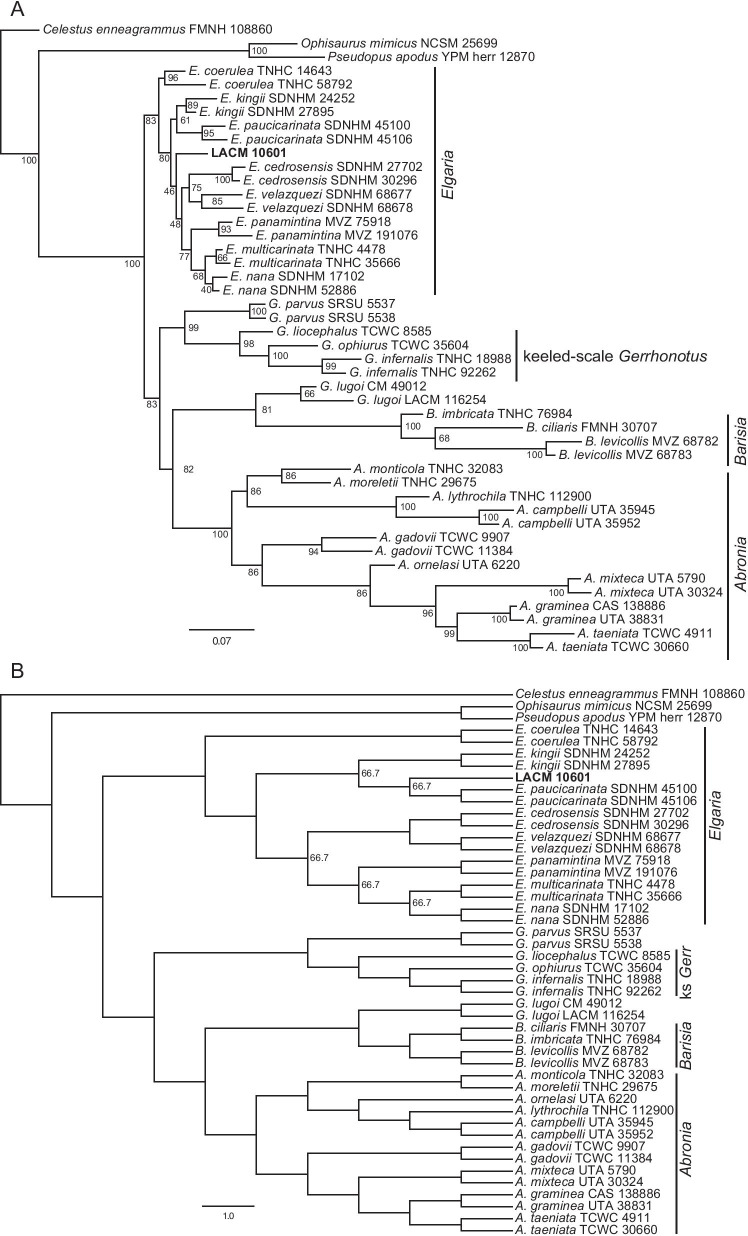
Majority-rule consensus trees from the fully constrained phylogenetic analyses in which *G. parvus* was constrained to be the sister taxon of the keeled-scale *Gerrhonotus*. For the Bayesian analysis, node values are posterior probabilities as a percent. For the parsimony analysis, node values indicate the percent of MPTs in which a given node occurred; if no value is listed, the node occurred in all MPTs. **a** Bayesian analysis. **b** Parsimony analysis

In all unconstrained and scaffold analyses *G*. *lugoi* was sister to *Barisia* (Figs. 12, 13, 14). The position of *A*. *ornelasi* varied. In all Bayesian analyses and the unconstrained parsimony analysis (Figs. 12, 13a, 14a), the species was sister to ((*A*. *graminea*, *A*. *taeniata*), *A*. *mixteca*) or (*A*. *graminea*, *A*. *mixteca*), but in the constrained parsimony analyses it was sister to species of *Auriculabronia* (Figs. 13b, 14b). The latter result is more consistent with previous analyses of morphological and molecular datasets [8, 25, 67]. The position of *G*. *parvus* was even more variable, mirroring difficulties previously encountered by researchers attempting to infer the relationships of the species [26]. *Gerrhonotus parvus* and *G*. *lugoi*, which are both part of the ‘smooth-scale’ group of *Gerrhonotus*, were never inferred to be sister taxa. In the unconstrained parsimony analysis *G*. *parvus* was sister to *E*. *cedrosensis*, and that clade was sister to the keeled-scale *Gerrhonotus* (Fig. 12b). In the unconstrained Bayesian analysis, *E*. *cedrosensis* was sister to the clade (*G*. *parvus*, keeled-scale *Gerrhonotus*) (Fig. 12a). In the constrained Bayesian analyses in which *G*. *parvus* could attach anywhere (Fig. 13a), the species was placed as sister to *Elgaria*. In the constrained parsimony analysis that allowed *G*. *parvus* to attach anywhere, the taxon was placed in a polytomy with the keeled-scale *Gerrhonotus* + the clade (*Abronia*, (*Barisia*, *G*. *lugoi*)) in the majority rule consensus tree (Fig. 13b), but was in a polytomy with many gerrhonotines in the strict consensus tree (see Additional file 1: Fig. S1). *Gerrhonotus parvus* is similar to *Elgaria* in lacking contact of the maxilla and the frontal and in lacking an ossified bridge between the nasal process and the body of the premaxilla, but possesses several features that are largely restricted to *Abronia* or *Barisia*, including contact of the premaxilla and the frontal, a low posterodorsal lamina of the vomer, reduced sublabial osteoderms, and the absence of a supratrigeminal process on the prootic. Contact of the premaxilla and the frontal and the absence of a supratrigeminal process were also observed in some specimens of *G*. *infernalis* and *G. lugoi*.

We note that for the parsimony analyses, the tree length of the scaffold trees was distinctly but not greatly longer than that of the unconstrained analyses. The tree length of the unconstrained parsimony tree was 324 steps, and the length of both constrained parsimony trees was 343 steps.

### Relationships of *Elgaria peludoverde*

*Elgaria peludoverde* was always inferred to be part of crown *Elgaria*. Relationships of *E*. *peludoverde* among *Elgaria* were largely consistent, but were poorly supported. Almost all relationships among *Elgaria*, including sister taxon relationships that were strongly supported by molecular analyses [5] and that were constrained here in scaffold analyses, were poorly supported (Figs. 12, 13, 14).

**Fig. 15:**
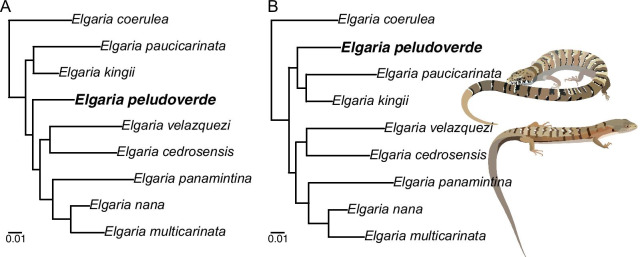
Relationships among *Elgaria* (based on [5]) and showing the two alternative hypotheses of the relationships of *E. peludoverde*. **a** The Bayesian analysis in which *G. parvus* was constrained to be the sister taxon of the keeled-scale *Gerrhonotus*. **b** The Bayesian analysis in which *G. parvus* could attach anywhere. The top illustration is *E. kingii* (drawn from a photo with a CC BY-NC 4.0 license, see https://creativecommons.org/licenses/by-nc/4.0/, courtesy of Kory Roberts and downloaded from https://www.inaturalist.org/observations/27879246) and the bottom illustration is of *E. multicarinata* (drawn from a photo taken by SGS)

In strict consensus trees from the parsimony analyses, *E*. *peludoverde* was in a polytomy with other species of *Elgaria* (excluding *E*. *coerulea*), or in a polytomy with other many gerrhonotines (Additional file 1: Figs S1–S3). In the majority rule trees from the constrained parsimony analyses, *E*. *peludoverde* was sister to *E*. *paucicarinata*, occuring in 66.7% of most parsimonious trees (MPTs) in both consensus trees (Figs. 13b, 14b), and in the unconstrained analysis the new taxon was sister to *E*. *paucicarinata* SDNHM 45106 (Fig. 12b). In the other MPTs from the parsimony analyses, *E. peludoverde* was sister to *E*. *panamintina*. In the unconstrained Bayesian analysis, *E*. *peludoverde* was sister to (*E*. *kingii* SDNHM 27895, *E*. *velazquezi* SDNHM 68678) (Fig. 12a). In the Bayesian scaffold analysis that allowed *G*. *parvus* to attach anywhere, *E*. *peludoverde* was sister to (*E*. *kingii*, *E*. *paucicarinata*) (Figs. 13a, 15b). In the Bayesian scaffold analysis in which *G*. *parvus* was placed as the sister taxon of the keeled-scale *Gerrhonotus*, *E*. *peludoverde* was placed as the sister taxon of the clade ((*E. cedrosensis*, *E. velazquezi*), ((*E. multicarinata*, *E. nana*), *E. panamintina*)) (Figs. 14a, 15a). That relationship was similar to the majority rule scaffold tree from Ledesma [41]. Although the relationships of *E*. *peludoverde* were not well-supported in any analysis, a close relationship with *E*. *paucicarinata* and/or *E*. *kingii* was recurring.

## Discussion

### Phylogenetic relationships of alligator lizards

We inferred relationships among gerrhonotine lizards using specimen-based phylogenetic analyses of a new osteological dataset. We corroborated several well-supported relationships previously inferred in phylogenetic analyses (see “Results”). Although we observed substantial inter- and intraspecific variation among many gerrhonotines (see "Phylogenetic character list" in "Materials and methods"), the main difficulty we encountered estimating phylogenetic relationships among extant gerrhonotines was with the interrelationships of *Elgaria*. That result was expected given the high amount of intraspecific variation in that genus that we documented previously [14].

The phylogenies and osteological data that we present here will be helpful towards future efforts to illuminate the relationships of two systematically enigmatic species of *Gerrhonotus*, *G. parvus* and *G. lugoi*. In both unconstrained analyses, *G. parvus* was in a clade with the keeled-scale *Gerrhonotus*, a relationship previously found by Conroy et al. [68], García-Vázquez et al. [26], and Zheng and Wiens [25]. In the partially constrained analyses which allowed *G. parvus* to attach anywhere, the species was placed in a polytomy with *Abronia*, *Barisia*, and the keeled-scale *Gerrhonotus* in the parsimony analysis (Fig. 13b), but the species was inferred to be the sister taxon of *Elgaria* in the Bayesian analysis (Fig. 13a). The former result is more consistent with previous molecular phylogenies and the presence of several shared features between *G. parvus* and *Abronia*, *Barisia*, and *Gerrhonotus*; the latter result may reflect convergence in shared character states between *G. parvus* and some species of *Elgaria*. Phylogenomic data may be required to provide resolution to the relationships of *G. parvus*. In all of our analyses, *G. lugoi* was the sister taxon of *Barisia*, a novel relationship first inferred by García-Vázquez et al. [26]; we provide the first osteological phylogeny supporting that relationship. That said, the presence of extensive patches of pterygoid teeth in *G. lugoi* is surprising given a close relationship between that species and *Barisia*, because we interpret a reduction of the pterygoid teeth as a derived character state in the clade containing *Barisia* and *Abronia*.

One notable discrepancy between our unconstrained phylogenies and recently published molecular phylogenies is the relationships among *Abronia*. We accept a recent phylogenomic hypothesis indicating paraphyly between *Abronia* and the previously recognized genus *Mesaspis* [10], but we did not find a paraphyletic *Abronia* (as it was previously circumscribed) in the present study. Instead, the terrestrial (‘*Mesaspis*’) and arboreal (‘*Abronia*’) morphotypes were monophyletic and were sister taxa, as was previously inferred from morphological data [8, 12]. The repeated appearance of those morphotypes and their associated ecologies is a fascinating issue that will hopefully be addressed in future genomic, ecological, and morphological studies.

### Osteological apomorphies of gerrhonotine lizards

Here, we list some morphological character states that we interpret as apomorphic of specific alligator lizards. These character states are interpreted through the constrained trees based on previously published molecular phylogenies and the taxonomies that reflect those phylogenies [5, 10, 26], not through the unconstrained trees from this study. We recommend that none of these characters be used individually in the absence of other morphological characters to frame a morphological diagnosis for any given fossil. Instead, we recommend that combinations of characters be assessed in a qualitative apomorphy-based diagnosis, or that a phylogenetic analysis be performed using the matrix that we provide here. We observed inter- and/or intraspecific variation in most character states that we consider apomorphic of a given species or clade. Thus, our interpretation of a character state as an apomorphy does not imply that the character state is present in all of the members of the specified clade. This list is intended to guide fossil identifications but is not intended to be an unambiguous key or a comprehensive summary of our findings. It is necessary to review the "Phylogenetic character list" in "Materials and methods") and/or the matrix itself to determine whether a character state can be used to identify a fossil via apomorphy-based diagnosis given the variation that we observed. Additionally, should a tree topology different from those employed here be leveraged to evaluate a fossil using the morphological characters from our matrix, topological discrepancies and any resulting differences in interpretation would need to be assessed and accounted for [69, 70]. Finally, we note that although our study contains a much broader taxonomic sample of gerrhonotines than any previous osteological survey, including several species for which osteological data were not previously available, we only sampled one or two specimens per species because in most cases no skeletons were available and relatively few alcohol-preserved specimens were available. Future studies with increased sample sizes for each species will augment our knowledge of patterns of variation for the morphological characters that we examined [14].

Character states that we observed only in a given species or clade among gerrhonotines are marked with a *. The phylogenetic character number and apomorphic character state number are listed following the brief character state description in the format ‘(phylogenetic character; apomorphic character state)’. We consider the phylogenetic position of *G. lugoi* and *G. parvus* to be ambiguous for the time being. However, each species exhibits character states that we consider to be apomorphic of other gerrhonotine clades, so in those cases we note the presence of those states in *G. lugoi* and/or *G. parvus*.

### *Elgaria*

- Presence of a midline foramen on the anterior surface of the premaxilla (5; 1).
- Presence of a large patch of pterygoid teeth (46; 2). This character state is also present in *G. lugoi*.

### Unnamed clade containing the southern population of *Elgaria multicarinata* and *Elgaria nana*

- Presence of keels on some dorsal cranial osteoderms (72; 1)*.

### Unnamed clade containing *Gerrhonotus*, *Barisia* and *Abronia*

- Presence of a bridge between the nasal process and the body of the premaxilla (2; 1). This character state is present in *G. lugoi* but is absent in *G. parvus*.
- Contact of the frontal and the premaxilla (4; 1)*. This character state is present in both *G. lugoi* and *G. parvus*.
- Contact of the frontal and the maxilla (22; 1)*.

### Keeled-scale *Gerrhonotus*

- Flat surangular shelf (66; 1).
- Presence of a postrostral osteoderm (78; 1).
- Separation of the posterior and anterior internasal osteoderms by the supranasal osteoderm (80; 1).

### *Gerrhonotus infernalis*

- Anterior opening of the carotid foramen of the sphenoid is directed anteriorly (54; 1)*.

### *Gerrhonotus lugoi*

- Palatine process of the maxilla lacks a discrete medial projection (21; 2)*.

### Unnamed clade containing *Abronia* and *Barisia*

- Presence of a distinct surangular process of the dentary (61; 1).
- Absence of the pterygoid teeth (46; 0)*.
- Conch of the quadrate does not widen dorsally (51; 1)*.
- Anteroposterior groove beneath the parapet on the posterolateral face of the dentary (62; 1)*.
- Contact of each posterior internasal osteoderm with each prefrontal osteoderm (79; 1)*.

### *Barisia*

- Relatively long distance between anterior margin of the nasals and the premaxillary process of the maxilla (13; 1). This character state is also present in *G. lugoi*.
- Concave anterior face of the nasal process of the premaxilla (7; 1)*.
- Sculpturing on the lateral surface of the jugal (27; 1)*.
- Absence of a frontonasal osteoderm (77; 0)*.

### *Barisia levicollis*

- Nasal process of premaxilla does not narrow in width posteriorly (8; 1)*.
- Absence of the anteromedial process of the nasal (12; 1)*.
- Facial process of the maxilla lacks a medial inflection (19; 1)*.

### *Abronia*

- Anterior medial process of the coronoid does not extend past the last tooth position on the dentary (64; 0).
- Cranial osteoderms with vermiculate texture that are heavily sculptured (71; 1).
- Supraciliary osteoderm series spans some, but not all, of the orbit (75; 1).
- Posterior margin of frontal wide relative to anteroposterior length of frontal (31; 0)*.
- Poorly ossified anteroventral cranial osteoderms (73; 1)*.
- Absence of an osteoderm overlying the frontoparietal shield (76; 1)*.

### Eastern clade of *Abronia* (*Auriculabronia* and the *moreletii* clade)

- Presence of a midline foramen on the anterior surface of the premaxilla (5; 1).

### *Auriculabronia* (represented here by *Abronia campbelli* and *Abronia lythrochila*)

- Separation of the posterior and anterior internasal osteoderms by the supranasal osteoderm (80; 1).

### *Abronia campbelli*

- Two anterior projections of the supratemporal (37; 1)*.

### *moreletti clade* (represented here by *Abronia moreletti* and *Abronia monticola*)

- Presence of a ventrolateral plate on the vomerine process of the palatine (44; 1)*.

### *Abronia mixteca*

- Large posterior projection from the facial process of the maxilla (20; 1).
- Posterior end of squamosal faces ventrally (35; 1)*

### *Abronia graminea* species complex (*Abronia graminea* and *Abronia taeniata*)

- Absence of a bridge between the nasal process and the body of the premaxilla (2; 0).

### *Abronia graminea*

- Presence of a coronoid contribution to the adductor fossa (67; 1).
- Absence of an anterodorsal projection of the splenial above the anterior inferior alveolar foramen (63; 2)*.

### Phylogenetic relationships of *Elgaria peludoverde*

Based on our phylogenetic analyses *E. peludoverde* is a member of crown *Elgaria*, and so is the oldest known fossil of the crown clade. The new species was generally inferred to be sister to or closely related to *E. paucicarinata* and/or *E. kingii*, but in one of the majority rule consensus trees from a Bayesian analysis, the new taxon was sister to the clade ((*E. cedrosensis*, *E. velazquezi*), ((*E. multicarinata*, *E. nana*), *E. panamintina*)) (see “Results”). Neither of those hypotheses were well supported by nodal posterior probabilities, nor were relationships among extant species of *Elgaria* regardless of constraints on the tree topology. The difficulty we encountered inferring relationships among *Elgaria* including *E. peludoverde* corroborates our previous study of variation among *Elgaria* [14]. Despite that variation, the alternative hypotheses for the relationships of *E. peludoverde* were recurring, and so we are able to provide a discussion of the potential biogeographic impacts of the discovery (see below).

### Diversification and biogeographic history of *Elgaria*

Fossils that do not occur within the modern-day distributions of extant relatives can provide important biogeographic insights that can not be inferred from the distributions of extant species [71, 72]. Extant species of *Elgaria* inhabit parts of southern British Columbia and much of the western U.S., and range as far south as the city of Jalisco, Mexico as well as onto the Baja California peninsula [73, 74] (see Fig. 1). One extralimital occurrence of *Elgaria* is known from Wyoming during the middle Miocene [24]. Previously, a paucity of fossils identified to *Elgaria* using apomorphies made interpretation of the biogeographic history of the genus largely reliant on molecular or other non-paleontological information [4, 5]. Additionally, hypotheses of relationships among *Elgaria* fluctuated greatly over the years, and correspondingly, biogeographic hypotheses changed as well (e.g., compare hypotheses proposed by Good [4] with those proposed by Leavitt et al. [5])**.** We investigated the phylogenetic relationships of *E. peludoverde* with respect to other species of *Elgaria* and in the context of modern phylogenetic hypotheses based on molecular data [5]. We inferred two alternative hypotheses of the relationships of *E. peludoverde* (Figs. 13, 14, 15). Here, we discuss the evolutionary and biogeographic history of *Elgaria* based on those hypotheses, published molecular phylogenies and divergence time estimates for *Elgaria* [5], modern species distributions (Fig. 1), and Neogene tectonic events in the southwestern U.S. and northern Mexico.

*Elgaria peludoverde* is currently the only described extinct species of *Elgaria*. The locality in Anza-Borrego Desert State Park from which the type specimen was recovered is today inhabited by *E. multicarinata*, which now ranges from Washington south to the west coast of Baja California. Divergence-time analyses indicate that *E. coerulea* diverged from other species of *Elgaria* in the Miocene circa 15 Ma (95% highest posterior density interval 19.1–11.8 Ma) [5]. That age coincides with and may be related to an episode of basin and range extension in the Nevada portion of the Great Basin region that is estimated to have started 17–16 Ma and ended 12–10 Ma [75]. The clade (*E. kingii*, *E. paucicarinata*) was estimated to have diverged from the other species of *Elgaria* between 8.7 and 5.5 Ma [5]. That divergence may be related to an episode of basin and range deformation in the southwestern U.S. and parts of northern Mexico lasting from about 15–12 Ma to around 6 Ma [76, 77]. *Elgaria kingii* and *E. paucicarinata* were estimated to have diverged from each other between 6.8 and 2.1 Ma [5]. A trans-gulfian vicariance event [78] resulting from the rifting of the Baja California Peninsula from mainland Mexico was hypothesized to play a role in the divergence between *E. kingii* and *E. paucicarinata* [5]. The initial rifting of the Baja California peninsula is estimated to have begun around 14–12 Ma, with sea waters first separating the southern peninsula by 8 Ma, and by 6.3 Ma the Gulf of California seaway was fully developed [79].

*Elgaria cedrosensis* and *E. velazquezi* are thought to have diverged from each other between 5.3 and 2.9 Ma [5]. The divergences between these two species and between the clades (*E. cedrosensis*, *E. velazquezi*) and (*E. multicarinata*, *E. panamintina*) in the late Miocene to early Pliocene were hypothesized to be related to the presence of a mid-peninsula seaway or marine incursion that may have existed near present day San Ignacio and Santa Rosalina [5, 80, 81]. *Elgaria cedrosensis* only occurs on Isla Cedros, so a vicariant event related to an uplift of Isla Cedros during the late Pliocene to Pleistocene [82] may have been involved in the divergence of *E. cedrosensis* from *E. velazquezi* [5]. The southern population of *E. multicarinata* and *E. panamintina* were estimated to have diverged from each other during the Pliocene [5], although Banta [7] previously envisioned a possible split as late as the Pleistocene.

LACM 10601 was deposited between 3.33 Ma and 3.22 based on correlation of the magnetozone at which the fossil was collected with chron 2An.2r. In many of our phylogenetic analyses, including the constrained Bayesian analysis in which *G. parvus* could attach anywhere, the majority-rule tree from the unconstrained parsimony analysis, and the majority-rule trees from both constrained parsimony analyses, *E. peludoverde* was inferred to be sister to *E. paucicarinata* or to the clade (*E. paucicarinata*, *E. kingii*). If *E. peludoverde* is indeed most closely related to these extant species, it is possible that *E. peludoverde* represents a northern population of total clade *E. paucicarinata* that diverged because of the development of the mid-Miocene mid-peninsula seaway. If *E. peludoverde* is more closely related to *E. kingii*, *E. peludoverde* may represent a western population of total clade *E. kingii* that diverged because of marine incursions during which the Gulf of California reached much farther north and partly isolated the Baja California peninsula and parts of southern California from the mainland (c. 4–3 Ma) [83]. Alternatively, *E. peludoverde* may be a stem member of the clade (*E. kingii, E. paucicarinata*) that diverged as a result of the basin-and-range deformation in the southwest U.S. and parts of northern Mexico mentioned above.

In the Bayesian analysis in which *G. parvus* was constrained to be the sister taxon of the keeled-scale *Gerrhonotus*, *E. peludoverde* was a stem member of the clade ((*E. cedrosensis*, *E. velazquezi*), ((*E. multicarinata*, *E. nana*), *E. panamintina*)). That clade was previously estimated to have a basal divergence age around 6–4 Ma [5]. It is unknown how long the lineage of *E. peludoverde* persisted, but under this hypothesis it would have diverged before 6–4 Ma. The location of *E. peludoverde* in southern California during the Pliocene and its position as a stem member of the ((*E. cedrosensis*, *E. velazquezi*), ((*E. multicarinata*, *E. nana*), *E. panamintina*)) clade would support a scenario of a southward dispersal of species within this clade onto the Baja California Peninsula, similar to the proposal of Welsh [84], although southern dispersal likely occurred prior to the Pleistocene scenario suggested by Welsh [84].

We provided some discussion on the evolutionary and biogeographic history of *Elgaria*, but our data are not exhaustive. Future biogeographic and phylogenetic analysis aided by well-supported fossil identifications will continue to shape interpretations of the biogeographic history of *Elgaria*. However, large amounts of intraspecific variation among species of *Elgaria* will likely make unambiguous identification of fossils to clades within crown *Elgaria* difficult [14]. Future investigations may benefit from using techniques such as geometric morphometrics, which have shown promise for identification of fossil lizards [85, 86] and may prove useful for interpreting the fossil record of *Elgaria*.

### Variation and fossil identifications

We sampled a broad range of gerrhonotine species in order to identify LACM 10601, ultimately concluding that the fossil represents a previously undescribed and extinct species of *Elgaria*. We examined all described extant species of *Elgaria* as well as many representatives from other gerrhontotine lineages. Our sampling strategy allowed us to gain a broad perspective of patterns of variation in the skulls of extant gerrhonotines. One limitation that we faced was a paucity of skeletal specimens for many gerrhonotine species. We partially overcame this limitation by CT-scanning alcohol preserved specimens of species for which skeletal information was scarce or lacking altogether. Although our dataset sampled many species, it is evident that there is much to be gained by sampling additional species as well as increasing sample sizes within sampled species (e.g., Ledesma et al. [14]). Our work as paleontologists will be greatly aided by continued efforts to understand patterns of osteological variation in extant taxa because investigating patterns of variation in extant taxa helps us to recognize and confidently identify extinct taxa. Documentation of variation in extant taxa is therefore an immensely valuable undertaking and indeed for many extant taxa, and for squamates generally, we have much to learn about patterns of osteological variation [87]. Before we investigate variation, we must address the need for comparative material. This need can be met by growing and maintaining dry skeletal collections [87]. CT data can also serve to increase the number of available comparative skeletal specimens, especially for rare, endangered, or otherwise underrepresented taxa in collections (e.g., most species of *Abronia*). However, the use of CT data can be restrictive because scanning and scan-processing software and hardware are costly and previously acquired CT data may not be readily available to all researchers [88]. We made all CT data used in our study freely and readily available at MorphoSource.org so that other researchers can build upon what we have learned and advance our understanding of osteological variation in gerrhonotines.

## Conclusions

The fossil record of gerrhonotines includes many specimens, but most of those specimens are isolated and fragmented cranial elements. Thus, there is limited potential for phylogenetic placement of most fossil alligator lizards. Articulated skulls like the one described here therefore yield new and critical insights. For example, we were able to provide a biogeographic assessment of *Elgaria* that was not possible from previously described fossils. That is especially important because the biogeography of *Elgaria* cannot be comprehensively inferred from the modern distributions of the extant taxa alone. Alternative biogeographic hypotheses informed by the new species of *Elgaria* are consistent with the geological history of the western U.S. and western Mexico including the Baja California peninsula. The results of our study reemphasize the importance of fossils for developing more holistic perspectives of evolutionary history.

A refined interpretation of the skull LACM 10601, first described in 1989 [33], had to await computed tomography, which revealed the full cranial anatomy of the fossil and allowed the production of an expanded osteological dataset of the alligator lizards from Baja California and other areas south of the contiguous US. Many of those species are rare and not widely available in collections, certainly not as skeletons. Our effort to CT-scan alcohol-preserved specimens to elucidate patterns of variation within and among gerrhonotines was a necessary step towards developing a reliable interpretation of the fossil. That endeavor resulted in a significantly expanded understanding of the osteology and phylogeny of alligator lizards and the biogeography of *Elgaria*, including a new phylogenetic matrix and an assessment of the phylogenetic utility of previously used morphological characters. The osteological dataset contains many more species of gerrhonotine lizards than were previously examined in phylogenetic analyses of osteological data, and the matrix will be instrumental for future efforts to describe and systematically place fossil gerrhonotines.

Patterns of skeletal variation among gerrhonotines, and especially among *Elgaria*, are complex. Although our taxonomic sample was more robust than those of previous studies, we still do not have a full appreciation of patterns of skeletal variation in alligator lizards. Our sample size was low (n = 1) for several species (largely due to specimen availability), yet we still encountered variation in many skeletal elements. We performed specimen-based phylogenetic analyses to account for that variation and avoid condensing our observations of multiple specimens to single terminal taxa. Broadly, our study underscores the need for greater efforts to expand osteological datasets for gerrhonotines and for squamates generally and for renewed efforts to integrate variable morphological characters into phylogenetic analyses. Small sample sizes remain inadequate for addressing many questions about the biogeography and evolutionary morphology of squamates. Multiple representatives of lineages should be sampled in morphological studies—the future of morphological and paleontological research relies on greater understanding of the extent and consequences of variation in the skeletal system.

## Materials and methods

### Anatomical nomenclature

Nomenclature follows Evans [63] unless otherwise noted.

### High-resolution computed tomography

Scanning information for specimens of *Abronia*, *Barisia*, LACM 10601, and outgroup anguids is in Table 1. Scanning information for all specimens of *Elgaria* and *Gerrhonotus* was provided by Ledesma et al. [14]. Digital segmentation was performed in Avizo Lite (8 and 9) by DTL and SGS. For the fossil LACM 10601, we segmented out as many elements as possible, but left those for which we could not separate the matrix from bone. We used the magic wand tool with a lower value of 25,000 or higher. Manual selections were often necessary to separate bone from the matrix. For extant specimens, we segmented all elements, generally with the magic wand and a gray-scale range of 18,000 to 30,000, but we occasionally segmented using manual selections when bones were in close contact. Unless otherwise noted, all images are surface renderings in orthographic view.

**Table 1:** Scanning information for *Abronia*, *Barisia*, *Elgaria peludoverde* LACM 10601, and anguid outgroups used in this study

Genus	Species	Institution	Number	Scanner	Portion scanned	Date Scanned	Voltage (kV)	Amperage (mA)	Slices in XY plane	Voxel size (μm)
*Abronia*	*campbelli*	UTA	R-35945	NSI Scanner	Head	6/23/2017	150	0.2	1424	22.1
*Abronia*	*campbelli*	UTA	R-35952	NSI Scanner	Head	6/23/2017	150	0.2	1424	22.1
*Abronia*	*graminea*	CAS	138886	Phoenix Vtome x M	Whole body	?	100	0.2	2789	39.6
*Abronia*	*graminea*	UTA	R-38831	NSI Scanner	Head	12/4/2017	150	0.2	1833	13.5
*Abronia*	*lythrochila*	TNHC	112900	NSI Scanner	Head	2/27/2019	140	0.14	1905	17.2
*Abronia*	*mixteca*	UTA	R-5790	NSI Scanner	Head	6/23/2017	150	0.2	1758	19.0
*Abronia*	*mixteca*	UTA	R-30324	NSI Scanner	Head	6/23/2017	150	0.2	1758	19.0
*Abronia*	*ornelasi*	UTA	R-6220	NSI Scanner	Head	6/26/2018	120	0.14	1873	10.9
*Abronia*	*taeniata*	TCWC	4911	NSI Scanner	Head	1/15/2020	120	0.17	1899	15.4
*Abronia*	*taeniata*	TCWC	30660	NSI Scanner	Head	1/15/2020	120	0.18	1857	15.6
*Abronia*	*gadovii*	TCWC	9907	NSI Scanner	Head	1/15/2020	120	0.15	1872	10.7
*Abronia*	*gadovii*	TCWC	11384	NSI Scanner	Head	1/15/2020	120	0.18	1857	15.6
*Abronia*	*monticola*	TNHC	32083	NSI Scanner	Head	3/30/2017	150	0.2	1595	12.4
*Abronia*	*moreletii*	TNHC	29675	NSI Scanner	Head	4/3/2017	150	0.2	1807	9.7
*Barisia*	*ciliaris*	FMNH	30707	Phoenix Vtome x M	Whole body	?	90	0.2	1935	48.7
*Barisia*	*imbricata*	TNHC	76984	NSI Scanner	Head	3/28/2017	150	0.2	1713	14.0
*Barisia*	*levicollis*	MVZ	68782	NSI Scanner	Head	6/22/2018	120	0.14	1859	22.4
*Barisia*	*levicollis*	MVZ	68783	NSI Scanner	Head	6/22/2018	120	0.14	1670	22.4
*Elgaria*	*peludoverde*	LACM	10601	NSI Scanner	Head	2/24/2016	100	0.24	1883	16.8
*Celestes*	*enneagrammus*	FMNH	108860	Actis Scanner	Head	?	180	0.13	525	14.6 (xy), 36.0 (z)
*Ophisaurus*	*mimicus*	NCSM	25699	Phoenix Vtome x M	Head	?	100	0.2	467	20.45
*Pseudopus*	*apodus*	YPM	12870	Actis Scanner	Head	?	100	0.2	840	29.4 (xy), 63.3 (z)

### Phylogenetic analysis

*Celestus enneagrammus* FMNH 108860 was treated as the outgroup in all analyses, following the topology of Burbrink et al. [89]. We performed unconstrained analyses, and analyses with scaffolds based on published molecular phylogenies. In the scaffold analyses, relationships among *Elgaria* follow Leavitt et al. [5], those among *Gerrhonotus* follow García-Vázquez et al. [26], and those among *Abronia* follow Gutiérrez-Rodríguez et al. [10]. Relationships among the major clades (*Elgaria*, (keeled-scale *Gerrhonotus*, (*Barisia*, *Abronia*))) follow Zheng and Wiens [25]. The phylogenetic positions of *G*. *lugoi* and *G*. *parvus* were uncertain with respect to other *Gerrhonotus* in the analyses performed by García-Vázquez et al. [26]. Previous morphological analyses placed *G*. *parvus* in *Elgaria *[4, 90], although later work using molecular data rejected that hypothesis [68]. *Abronia ornelasi* was not sampled by Gutiérrez-Rodríguez et al. [10]. We performed analyses in which we allowed *A. ornelasi*, *G*. *lugoi*, and *G*. *parvus* to attach anywhere on the tree. We also performed analyses in which *A. ornelasi* and *G. lugoi* could attach anywhere, but *G. parvus* was constrained to be the sister taxon of the keeled-scale *Gerrhonotus*, a recurring relationship in the analyses by García-Vázquez et al. [26] that was also found by Conroy et al. [68] and Zheng and Wiens [25]. Relationships among *Barisia* were allowed to vary in all analyses.

We performed Bayesian analyses in MrBayes v. 3.2.7 [91]. We ran analyses for two runs of 2,000,000 generations and four MCMC chains sampled every 1000 generations. The symmetric dirichlet hyperprior was set at infinity and lset coding was set to variable. We used Tracer v 1.7 [92] to confirm convergence of each run (effective sample size values > 200). The posterior distributions of trees were summarized as 50% majority rule consensus trees (sumt command). We conducted the parsimony analyses in PAUP* 4.0 [93] using a heuristic search, 10,000 replicates, random taxon addition, and with multistate codings treated as polymorphic. The resulting MPTs were summarized as strict consensus trees and 50% majority rule consensus trees. For all analyses, characters were treated as unordered and equally weighted, and should be treated as such in all future analyses of this matrix.

### Phylogenetic matrix construction

#### Taxon sampling

Apomorphy-based diagnosis established that LACM 10601 is a gerrhonotine (see “Results”). In the new matrix presented here, we only included CT-scanned gerrhonotines and CT-scanned diploglossine and anguine outgroups. We also only scored skeletally mature specimens for phylogenetic analyses. We examined supplemental CT-scanned specimens of juvenile gerrhonotines and adult *Xenosaurus*, and traditionally prepared skeletal material of gerrhonotines, diploglossines, anguines, and *Xenosaurus* (see Additional file 1).

Skeletal maturity was assessed via comparison to individual bone ontogenies described by Bhullar [62] and supplemented by our previous observations in *Elgaria *and *Gerrhonotus* [14]. Specifically, we established whether specimens were mature by examining the development of the parietal (constriction of the parietal table present, well-developed osteodermal crust on parietal present), otooccipital (relatively long paroccipital processes present), and supraoccipital (bone long relative to its width in dorsal view). Both specimens of *G. parvus* and *A. campbelli* UTA 35945 lack the constriction of the parietal table that is associated with skeletal maturity. The specimens of *G. parvus* (SRSU 5538 and SRSU 5537, the holotype and the paratype, respectively) are known to be adults because previous examination of the ovaries of the holotype indicated a mature individual, and the paratype was kept alive in captivity for five years prior to preparation in alcohol [94]. *Abronia campbelli* UTA 35945 is a large and robust specimen comparable in size to the other specimen of *A. campbelli*, and the other ontogenetic measures indicate a skeletally mature individual.

Our sample of extant gerrhonotines scored for the phylogenetic matrix consists of 25 species and 42 specimens. We examined all eight species of *Elgaria*, five species of *Gerronotus*, three species of *Barisia*, and nine species of *Abronia* (three of which were until recently placed in the genus *Mesaspis*). For outgroups, we sampled the anguids *Ophisaurus mimicus*, *Pseudopus apodus*, and *Celestus enneagrammus* (n = 1 for each). When possible, we scanned, examined, and scored two specimens of each species of gerrhonotine to accommodate at least some intraspecific variation. We sampled several lineages within each nominal genus, recognizing that several of those genera are not/may not be monophyletic. Although our sampling represents a substantial improvement over previous studies that examined the phylogeny of gerrhonotines from an osteological perspective, we emphasize that the sample is not exhaustive in terms of species nor is it robust in terms of number of specimens sampled.

The northern and southern populations of *E. multicarinata* were paraphyletic with respect to *E. panamintina* in the analyses of Leavitt et al. [5], so we restricted our CT data for *E. multicarinata* to the southern population. We did not observe consistent osteological differences between the northern and southern populations in a previous study [14]. Additionally, *E. nana* was not treated as a species separate from *E. multicarinata* by Leavitt et al. [5], but we treat it as a separate species here following Grismer [74] and pending further investigation. We sampled two specimens of each species of *Elgaria*.

*Gerrhonotus* is putatively non-monophyletic. A recent molecular phylogeny indicated that *G. lugoi* may be more closely related to *Barisia* than to other species of *Gerrhonotus*, and support for a sister-taxon relationship between *G. parvus* and the keeled-scale species of *Gerrhonotus* (e.g., *G. infernalis*, *G. liocephalus*, and *G. ophiurus*) was recurring but weak [26]. *Gerrhonotus infernalis* is probably paraphyletic as well [26]; both of our CT-scanned specimens are from Texas and so can be confidently ascribed to the same lineage.

For *Barisia*, we sampled *B. levicollis* (n = 2), *B. ciliaris* (n = 1), and *B. imbricata* (n = 1). The specimens of *B. ciliaris* and *B. imbricata* are both identified as *B. imbricata ciliaris*, but only one (FMNH 30707) falls within the range of *B. ciliaris*, in Nuevo León*.* The other specimen (TNHC 76984) was collected in the state of Hidalgo well within the currently recognized range of *B. imbricata* [95].

*Abronia* is paraphyletic with respect to the previously recognized genus *Mesaspis*. In a phylogenetic study of external morphological data, monophyly of *Abronia* (excluding *Mesaspis*) was supported but was not considered unambiguous [8]. Monophyly of *Abronia* (excluding *Mesaspis*) was also supported by a phylogenetic study of osteological data [4]. In molecular studies of Sanger-sequenced exonic and mitochondrial data, *Abronia* and *Mesaspis* were paraphyletic with respect to each other, but most relationships among the two groups were inferred with poor support [25, 67, 96]. Most recently, the authors of a phylogenomic study that used double-digest restriction site-associated (ddRADseq) data presented persuasive evidence of wide-spread paraphyly of *Abronia* and *Mesaspis* with respect to each other [10]. Those authors found two major clades distributed on either side of the Isthmus of Tehuantepec, each containing multiple clades of ‘*Abronia*’ and ‘*Mesaspis*.’ That result indicated that the geography of Mesoamerica explains the phylogeny of extant species *Abronia* and *Mesaspis*, as opposed to life history, the existing genus-level taxonomy, or previously proposed morphological apomorphies and/or diagnostic features, and consequently, the authors [10] recommended that species of *Mesaspis* Cope, 1877 [97] be placed in *Abronia *Gray, 1838 [57].

We endeavored to sample multiple clades that are or were placed in *Abronia, Mesaspis*, and *Gerrhonotus*. We follow the recommended taxonomy of Gutiérrez-Rodríguez et al. [10]; we consider all species formerly assigned to *Mesaspis* to be part of the genus *Abronia*. We sampled the clades *Abaculabronia* (*A. ornelasi*, n = 1), *Auriculabronia* (*A. campbelli*, n = 2; *A. lythrochila*, n = 1), members of the *taeniata* complex (*A. graminea*, *A. taeniata*, n = 2 for both), and a member of the *oaxacae* group (*A. mixteca*, n = 2). For species of *Abronia* previously placed in *Mesaspis*, we sampled *A. gadovii* (n = 2) and the *moreletti* group (*A. moreletti* and *A. monticola*, n = 1 for both species). In total, we sampled six of the eleven major subclades of *Abronia*, including five of the eight subclades sampled by Gutiérrez-Rodríguez et al. [10] and one of three other putative subgenera (*Aenigmabronia*, *Lissabronia*, and *Abaculabronia*). For *Gerrhonotus*, we sampled several species of the keeled-scale clade (*G. infernalis*, n = 2; *G. liocephalus*, n = 1; *G. ophiurus*, n = 1) and two smooth-scale species (*G. lugoi* and *G. parvus*, n = 2 for both species).

In addition to our relatively broad taxonomic sampling, our sample includes several holotypes (*G. lugoi* CM 49012 and *G. parvus* SRSU 5538) and paratypes (*A. ornelasi* UTA 6220, *E. velazquezi* SDNHM 68677 and SDNHM 68678, and *G. parvus* SRSU 5537). We also sampled several species for which osteological data were not previously available (e.g., *A. campbelli*, *A. lythrochila*, *A. monticola*, *A. ornelasi*, *B. levicollis*).

### Specimen sampling

For the most part, we scored specimens that were scanned at UTCT specifically for this project or for related research projects [13, 14]. *Pseudopus apodus* YPM 12870 and *C. enneagrammus* FMNH 108860 were previously scanned at UTCT for the Squamate Tree of Life Project, and those data were downloaded from MorphoSource.org. One specimen each of *A. graminea* (CAS 138886), *B. ciliaris* (FMNH 30707), and *O. mimicus* (NCSM 25699) were also downloaded from MorphoSource.org, and were scanned at CAS, FMNH, and UF, respectively.

### Character sampling

Here, we outline our character selection criteria (see Poe and Wiens [98]). The matrix has 80 characters and 75 parsimony informative characters. All phylogenetic characters pertain to the skull because LACM 10601 is a fossil skull, and reported fossil gerrhonotines are almost exclusively cranial elements [38]. We included previously described cranial characters that could not be scored on the fossil, with the intent that those characters will be useful for future efforts to systematically place skulls or isolated cranial elements of fossil gerrhonotines. We used autapomorphic characters of individual species, characters that varied among species, and characters that varied within individual specimens. We did not establish new invariant characters. We scored some previously published characters that diagnose Anguidae with respect to Xenosauridae and which identified LACM 10601 as an anguid (see “Diagnosis”), and those characters are listed in the matrix description as *Invariant*. We made a concerted effort to not use a priori groupings (i.e., the present genus-level taxonomy or previously published phylogenies) to frame morphological characters or character state values.

We investigated the systematic utility of some variable morphological features that we ultimately decided to exclude from the phylogenetic matrix. We provide a list of excluded characters at the end of the character list, including our rationale for excluding those characters. That list includes both characters used in previous phylogenetic analyses and features not previously investigated for gerrhonotine systematics.

Character state numbers were assigned arbitrarily, and no character state transformations are treated as ordered. Characters that were bilaterally asymmetric within an individual specimen were treated as polymorphic (e.g., ‘(01)’). The study from which we derived a character is listed in parentheses after the description. In some cases, the systematic or diagnostic utility of a character was described previously without being explicitly framed for phylogenetic analysis, but those references are still listed.

Specimen numbers for individual specimens are listed when appropriate, including for species represented by only one specimen. In cases for which both examined specimens of a given taxon exhibit a morphological character, the species name is listed by itself (as opposed to repeatedly listing both specimen numbers).

### Phylogenetic character list

#### Premaxilla

**Fig. 16:**
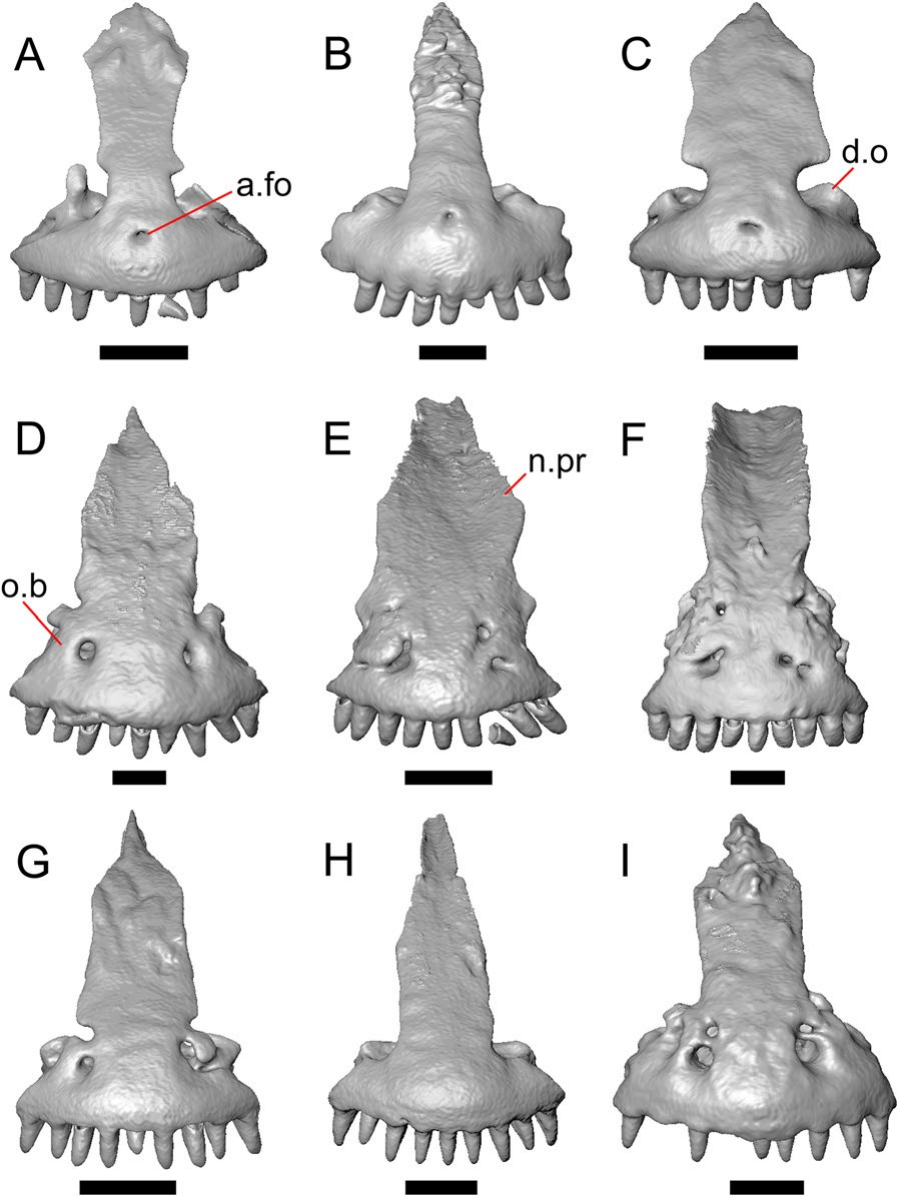
Premaxillae in anterior view. Scale bars = 1 mm. **a** *E. kingii* SDNHM 27985. **b** *E. velazquezi* SDNHM 68677. **c** *E. coerulea* TNHC 14643. **d** *G. infernalis* TNHC 18988. **e** *B. imbricata* TNHC 76984. **f** *B levicollis* MVZ 68783. **g** *Abronia gadovii* TCWC 9907. **h** *A. graminea* UTA 38831. **i** *A. lythrochila* TNHC 112900. *a.fo* anterior foramen, *d.o* dorsal ossification, *n.pr* nasal process, *o.b* ossified bridge

1. Raised dorsal ossification on the alveolar plate of the premaxilla posterolateral to the medial ethmoidal foramen (foramen for ophthalmic branch of CN5 of Evans [63]) (Scarpetta 2018 [24]): 0 = absent; 1 = present (Fig. 16c).

##### Remarks.

Gerrhonotines and diploglossines have a raised ossification on the dorsal surface of the alveolar plate posterolateral to the medial ethmoidal foramen, but the ossification is absent in examined anguines. The space between the nasal process and the alveolar plate is more heavily ossified in *Barisia* than in other gerrhonotines (see character 2), so the raised dorsal ossification is difficult to distinguish in anterior view (Fig. 16e, f).

2. Ossified bridge between the nasal process and the alveolar plate of the premaxilla (Good 1987 [12], characters 1 and 2; see Campbell and Frost 1993 [8]): 0 = absent (Fig. 16b, c, h); 1 = present (Fig. 16d–f, i).

##### Remarks.

In *Barisia*, *Gerrhonotus* (except *G. parvus* and *G. lugoi* CM 49012), *Abronia* (except *A. taeniata*, *A. graminea*, and *A. moreletti* TNHC 29675), and *E. kingii* SDNHM 24252, an ossified bridge connects the alveolar plate of the premaxilla to the nasal process. A bridge is absent in anguines and diploglossines. Ossification between the alveolar plate and the nasal process is more extensive in *Barisia* and some specimens of *Gerrhonotus* relative to other gerrhonotines, so the connection is a mass of bone instead of a bridge-like structure.

**Fig. 17:**
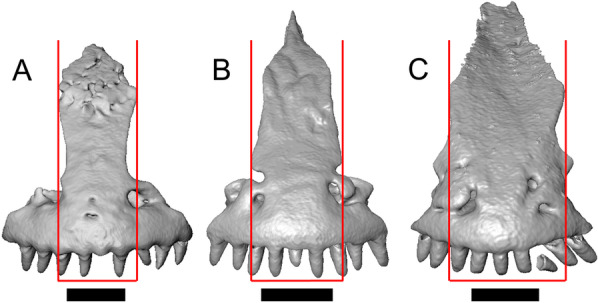
Premaxillae in anterior view illustrating the relative widths of the nasal process. The red lines illustrate where the width of the nasal process is widest for each specimen. Scale bars = 1 mm. **a** *E. kingii* SDNHM 24252. **b** *Abronia gadovii* TCWC 9907. **c** *B. imbricata* TNHC 76984

Bilateral asymmetry is present on *E. kingii* SDNHM 24252 (Fig. 17a) and *G. lugoi* LACM 116254, in which the bridge is present on the left side but not the right side. Those specimens were scored as polymorphic. In *E. kingii* SDNHM 27895 (Fig. 16a) and *A. moreletti* TNHC 29675, distinct projections from both sides of the nasal process nearly connect to the raised dorsal ossification, which attains an unusually tall height in both specimens. Those specimens are also scored as polymorphic. We also note that we and another author previously observed a prepared skeleton of *E. multicarinata* that has a fully ossified bridge [14, 61].

3. Width of the nasal process at its widest point, measured in tooth positions spanning the nasal process, in anterior view, divided by the total number of tooth positions (modified from Bhullar 2011 [60], character 21; Good 1987 [12], character 5 and 6).

0 =  < 0.473 (narrow; Fig. 17a); 1 = 0.473–0.618 (intermediate; Fig. 17b); 2 =  > 0.618 (broad; Fig. 17c).

##### Remarks.

Incomplete tooth positions of 0.5 or over were rounded to the next whole number (e.g., if the nasal process of the premaxilla was 3.5 tooth positions wide, that value was rounded up to 4 tooth positions). The tooth count spanning the nasal process was normalized by dividing the number of teeth spanned by the nasal process by the total number of premaxillary tooth positions. Gerrhonotines usually possess nine tooth positions on the premaxilla, but occasionally the central position is missing or an extra position is present on one side, resulting in eight or ten tooth positions, respectively. *Elgaria panamintina* MVZ 75918 and *E. coerulea* TNHC 14643 have eight premaxillary tooth positions, and *E. kingii* SDNHM 24252 has ten premaxillary tooth positions.

We used an automated classification method to assign our continuous data to discrete character states. We selected three states for the character and used the function ‘bin’ in the machine learning package OneR [99, 100] to bin the data into discrete states. We used the method ‘length,’ which splits the data into bins of equal length. We emphasize that these categories are specific to the specimens measured here. Should other specimens be scored for this character (and other characters that we discretized using this method), the categorization analysis would need to be repeated and revised character state boundaries established.

Given those categories, we found that the nasal process is narrow in most gerrhonotines in our sample. The nasal process is comparatively wider in *A. mixteca*, *A. gadovii*, *A. monticola* TNHC 32083, *B. levicollis*, *G. infernalis*, *G. ophiurus* TCWC 35604, and a few specimens of *Elgaria* (e.g., *E. multicarinata* TNHC 35666, *E. coerulea* TNHC 14643). The nasal process is exceptionally broad in *C. enneagrammus* FMNH 108860, *B. ciliaris* FMNH 30707, and *B. imbricata* TNHC 76984. In *B. imbricata* TNHC 76984, there are two projections from the ossified bridge that appear to be part of the nasal process in anterior view (see Fig. 17c), but that we do not consider to be part of the nasal process.

**Fig. 18:**
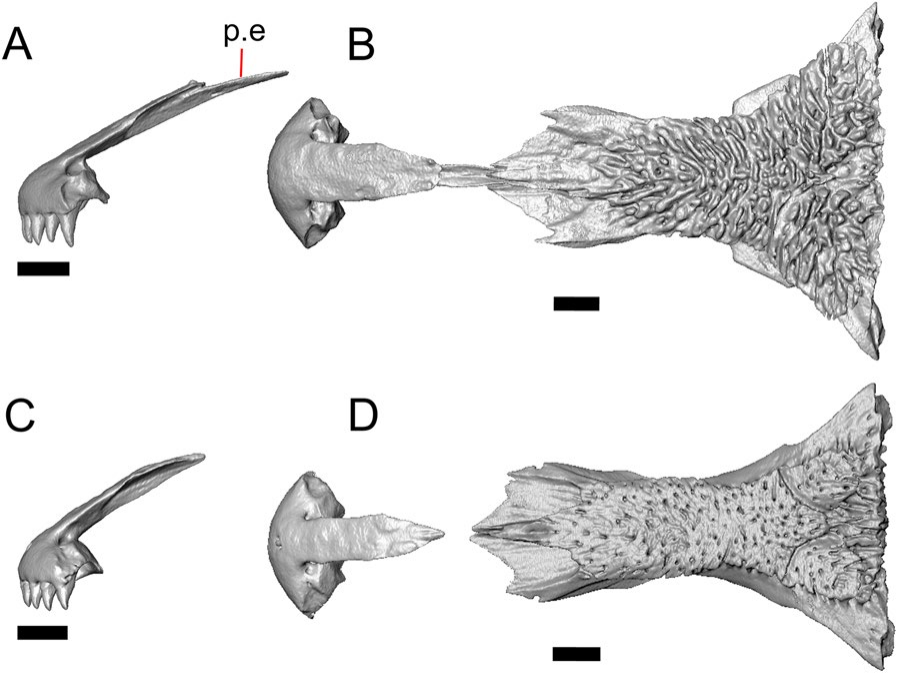
Premaxillae and contact (or lack thereof) between the premaxilla and the frontal. Scale bars = 1 mm. **a**, **b**, are *A. graminea* UTA 38831, **c, d** are *E. paucicarinata* SDNHM 45100. **a** Premaxilla in lateral view. **b** Premaxilla and frontal in dorsal view. **c** Premaxilla in lateral view. **d** Premaxilla and frontal in dorsal view. *p.e* posteroventral extension

4. Contact of the premaxilla and the frontal (modified from Good 1987 [12], character 9; Conrad et al. 2011 [18], character 13): 0 = absent (Fig. 18d); 1 = present (Fig. 18b).

##### Remarks.

Contact is scored as present when the premaxilla and the frontal are in contact, or overlap anteroposteriorly but are narrowly separated in the dorsoventral dimension. Contact is scored as absent when the premaxilla and frontal are not close to contacting, or are close to contacting but do not overlap in the anteroposterior dimension. Premaxilla-frontal contact is present in *A. graminea*, *A. mixteca*, *A. taeniata* TCWC 30660, *A. gadovii*, *A. monticola* TNHC 32083, *Barisia*, *G. infernalis* TNHC 92262, *G. lugoi*, and *G. parvus*. In most of those taxa, contact is present because there is a long and thin extension of the nasal process of the premaxilla posterior to the main body of the nasal process [14]. The posterior extension is absent in *G. infernalis* TNHC 92262. The nasal process appears elongated in *Barisia* relative to *Elgaria* and other gerrhonotines that lack the posterior extension of the nasal process, but in *Barisia* there is little width differentiation between the anterior and posterior portions of the process, especially in *B. levicollis*. Thus, the presence or absence of the posterior extension is unclear in *Barisia*.

5. Single midline foramen or two midline foramina on the anterior surface of the alveolar plate of the premaxilla (Scarpetta 2018 [24]; Smith 2009 [23]): 0 = absent (Fig. 16d–i); 1 = present (Fig. 16a–c).

##### Remarks.

A single anterior midline foramen is present on the anterior surface of the premaxilla in most species of *Elgaria*, but is absent in *E. cedrosensis* and *E. panamintina*. The foramen is also present in *A. moreletti* TNHC 29675, *A. campbelli*, and *Ophisaurus* [24]. Like Scarpetta [24], we interpret the foramen as separately derived in *Ophisaurus* and gerrhonotines. *Elgaria kingii* SDNHM 24252 is singular among examined specimens in that there are two midline foramina in close proximity and in a dorsoventral row. We assume that the two foramina of *E. kingii* SDNHM 24252 are homologous to the single foramen of other gerrhonotines and scored the specimen as state 1. In *E. paucicarinata* SDNHM 45100, a single midline foramen is divided by an anterior midline septum.

**Fig. 19:**
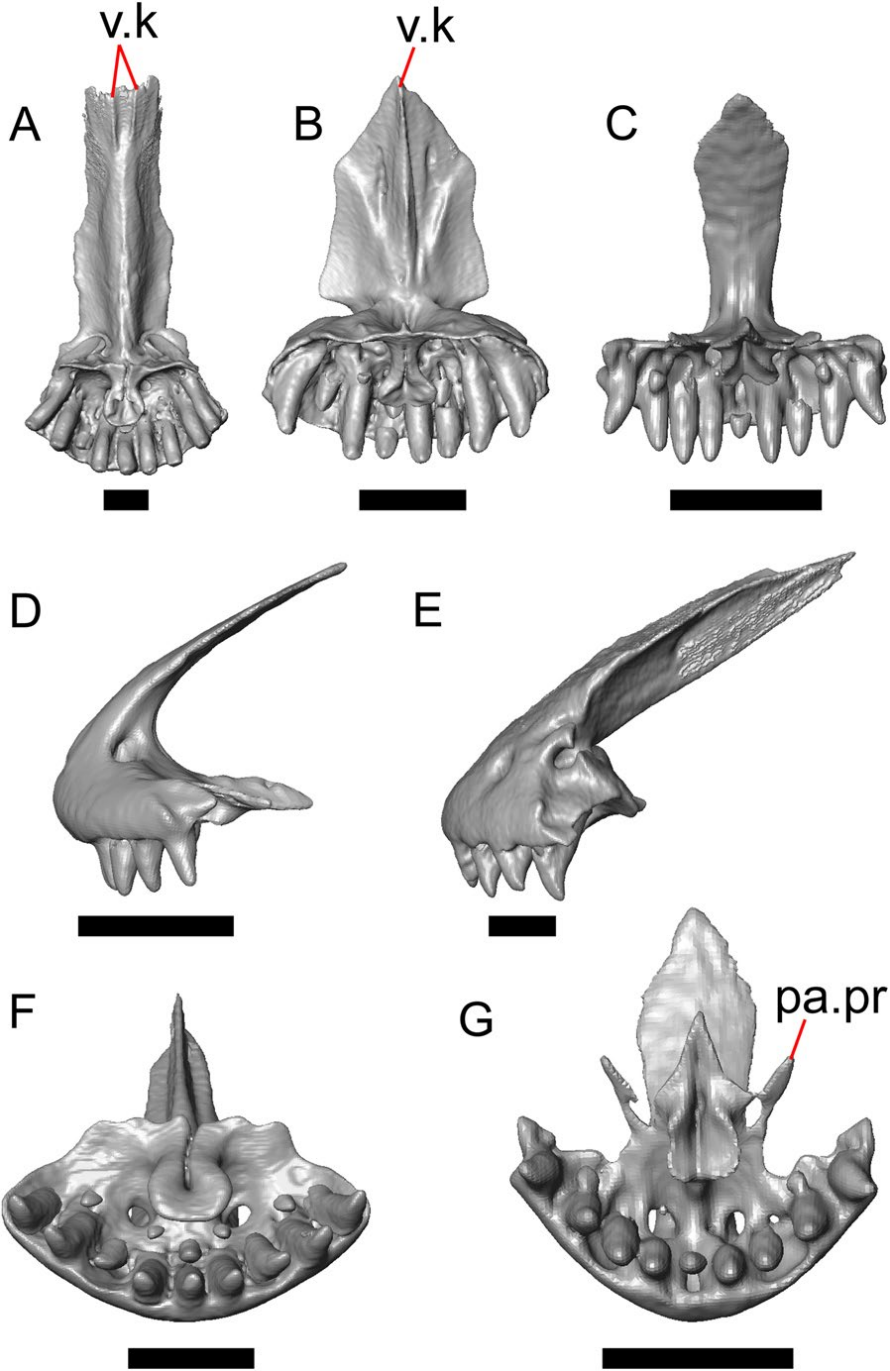
Premaxillae of anguid lizards. Scale bars = 1 mm. **a** *B. levicollis* MVZ 68782 in posterior view. **b** *E. coerulea* TNHC 14643 in posterior view. **c** *O. mimicus* NCSM 25699 in posterior view. **d** *O. mimicus* NCSM 25699 in left lateral view. **e** *G. infernalis* TNHC 198988 in left lateral view. **f** *E. velazquezi* SDNHM 68678 in ventral view. **g** *O. mimicus* NCSM 25699 in ventral view. *pa.pr* palatal process, *v.k* ventral keel

6. Anterior face of the premaxilla (modified from Scarpetta 2018 [24]): 0 = nearly flush with alveolar margin (Fig. 19e); 1 = protrudes anteriorly well past the alveolar margin (Fig. 19d).

##### Remarks.

In anguines the premaxilla is convex anteriorly, protruding well past the alveolar margin. In gerrhonotines and diploglossines, the anterior face of the premaxilla is nearly flush with the alveolar margin.

7. Shape of the dorsal surface of the nasal process of the premaxilla (new): 0 = slightly convex or flat (Fig. 16a–d, g–i); 1 = concave (Fig. 16e, f).

##### Remarks.

The dorsal surface of the nasal process is convex or almost flat in most gerrhonotines, but is concave in *Barisia*.

8. Morphology of the posterior portion of the nasal process of the premaxilla (Ledesma et al. 2021 [14]): 0 = posterior portion of the nasal process tapers relative to the immediately proximal portion of the process (Fig. 16a–e, g–i); 1 = posterior margin of the nasal process is broad and does not taper (Fig. 16f).

##### Remarks.

The posterior end of the nasal process tapers in width in most gerrhonotines, both in taxa with a posterior extension and in those without. In *B. levicollis,* the posterior portion of the nasal process does not taper and retains a width similar to the anterior portion of the process.

9. Ventral keel on the nasal process of the premaxilla (modified from Bhullar 2011 [60], character 24): 0 = present (Fig. 19a, b); 1 = absent (Fig. 19c).

##### Remarks.

The ventral keel on the nasal process is well-developed in gerrhonotines, and is absent in examined anguines and diploglossines.

10. Forked posterior end of the ventral keel of the nasal process of the premaxilla (new): 0 = absent (Fig. 19b, c); 1 = present (Fig. 19a).

##### Remarks.

The posterior end of the ventral keel of the nasal process is forked, creating two posterior keels, in *A. mixteca*, *A. gadovii*, *Barisia*, and *G. lugoi* LACM 116254. The keel terminates as a single keel in all other gerrhonotines.

11. Distinct palatal processes of the premaxilla (modified from Evans 2008 [63]; Conrad et al. 2011 [18], character 17): 0 = absent (Fig. 19f); 1 = present (Fig. 19g).

##### Remarks.

In gerrhonotines, the alveolar plate is a single surface from which distinct palatal processes do not extend. In anguines and diploglossines, there are discrete processes that meet the maxilla laterally and the vomers posteriorly that create an anterior fontanelle between the premaxilla and the maxilla.

### Nasal

**Fig. 20:**
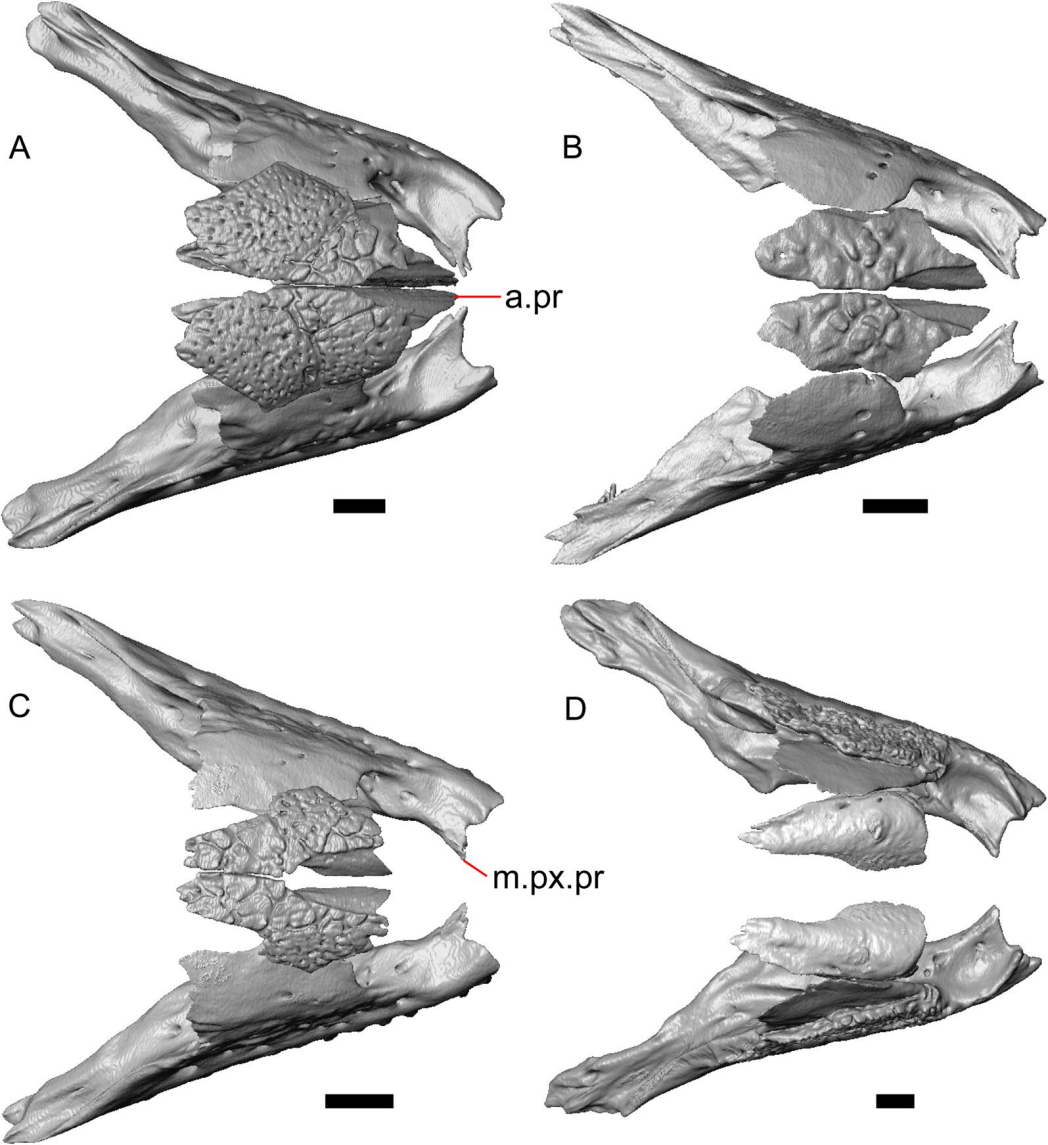
Nasals and maxillae in dorsal view. Scale bars = 1 mm. **a** *E. nana* SDNHM 17102. **b** *A. ornelasi* UTA 6220. **c** *G. lugoi* LACM 116254. **d** *B. levicollis* MVZ 68782. *a.pr* anterior process, *m.px.pr* medialmost inflection of the premaxillary process of the maxilla

12. Anteromedial process of the nasal (new): 0 = present (Fig. 20a–c); 1 = absent (Fig. 20d).

#### Remarks.

In most gerrhonotines, the premaxillary facet of the nasal has a subtriangular anteromedial process that articulates with the nasal process of the premaxilla. The anteromedial process of the nasal is absent in *B*. *levicollis*.

13. Distance between the anterior level of the medialmost inflection of the premaxillary process of the maxilla and the anterior end of the premaxillary shelf on the nasal (derived from Good 1987 [12], characters 53 and 54). 0 = relatively short (Fig. 20c, d); 1 = relatively long (Fig. 20a, b).

#### Remarks.

In most gerrhonotines, the distance between the anterior margin of the nasal and the anteriormost inflection of the premaxillary process of the maxilla is short. That distance is substantially longer in *Barisia* and *G*. *lugoi*. The scoring of this character is not dependent on the presence or absence of the anteromedial process because the distance is long in *G*. *lugoi*, *B*. *ciliaris* FMNH 30707, and *B*. *imbricata* TNHC 76984 in addition to *B*. *levicollis* (see character 12).

14. Contact of the nasals with one another near their anterior–posterior midpoint (derived from Good 1987 [12], character 54): 0 = nasals contact (Fig. 20a); 1 = nasals are separated (Fig. 20b–d).

#### Remarks.

The nasals are separated near their anterior–posterior midpoint in *A. graminea*, *A. mixteca*, *A. ornelasi* UTA R-6220, *A. gadovii*, *Barisia*, and *G. lugoi*. Contact between the nasal process of the premaxilla and the frontal was previously hypothesized to exclude contact of the nasals in *Abronia*, *Barisia*, and *Abronia* (*Mesaspis*) *gadovii* [12]. We did not find that separation of the nasals was tied to the contact of the premaxilla and the frontal, and so consider those features to be separate characters. For example, the nasals of *G. parvus* SRSU 5538 and *A. taeniata* TCWC 30660 are in close contact anteriorly and near their longitudinal midpoint, but the premaxilla and frontal are in broad contact. In *A. ornelasi* UTA R-6220, the nasals are broadly separated anteriorly and near their midpoint, but the premaxilla and frontal do not contact.

### Septomaxilla

**Fig. 21:**
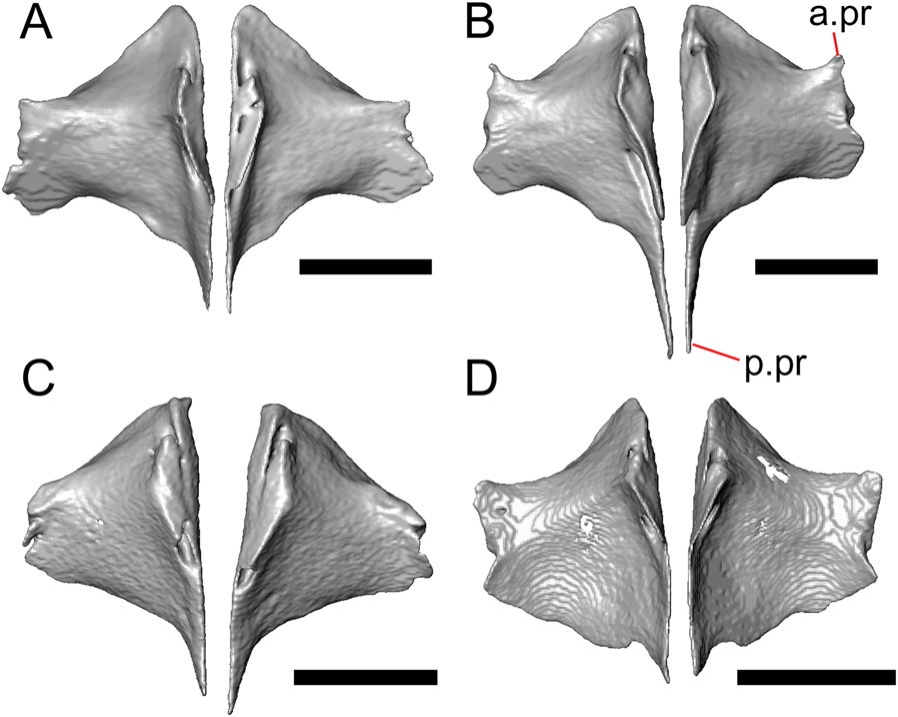
Septomaxillae. Scale bars = 1 mm. **a** *E. kingii* SDNHM 24252. **b** *E. paucicarinata* SDNHM 45106. **c** *A. gadovii* TCWC 9907. **d** *G. lugoi* LACM 116254. *a.pr* anterior process, *p.pr* posterior process

15. Posterior (septal) process of the septomaxilla (Good 1987 [12], character 62): 0 = relatively long (Fig. 21b); 1 = relatively short (Fig. 21a, c, d).

#### Remarks.

The posterior process of the septomaxilla is reduced in length compared to other gerrhonotines and other anguids in *G. lugoi*, *Barisia*, *A. monticola* TNHC 32083, *A. gadovii* TCWC 9907, *E. kingii* SDNHM 24252, *C. enneagrammus* FMNH 108860, and the right septomaxilla of *E. coerulea* TNHC 58792.

16. Anterolateral projection of the lateral plate of the septomaxilla (derived from Good 1987 [12], character 63): 0 = absent (Fig. 21c); 1 = present (Fig. 21a, b, d).

#### Remarks.

The anterolateral projection of the septomaxilla is absent in *A. taeniata* TCWC 30660, *A. moreletti*, *A. monticola* TNHC 32083, *A. gadovii* TCWC 9907, *B. ciliaris* FMNH 30707, and *B. imbricata* TNHC 76984.

### Maxilla

**Fig. 22:**
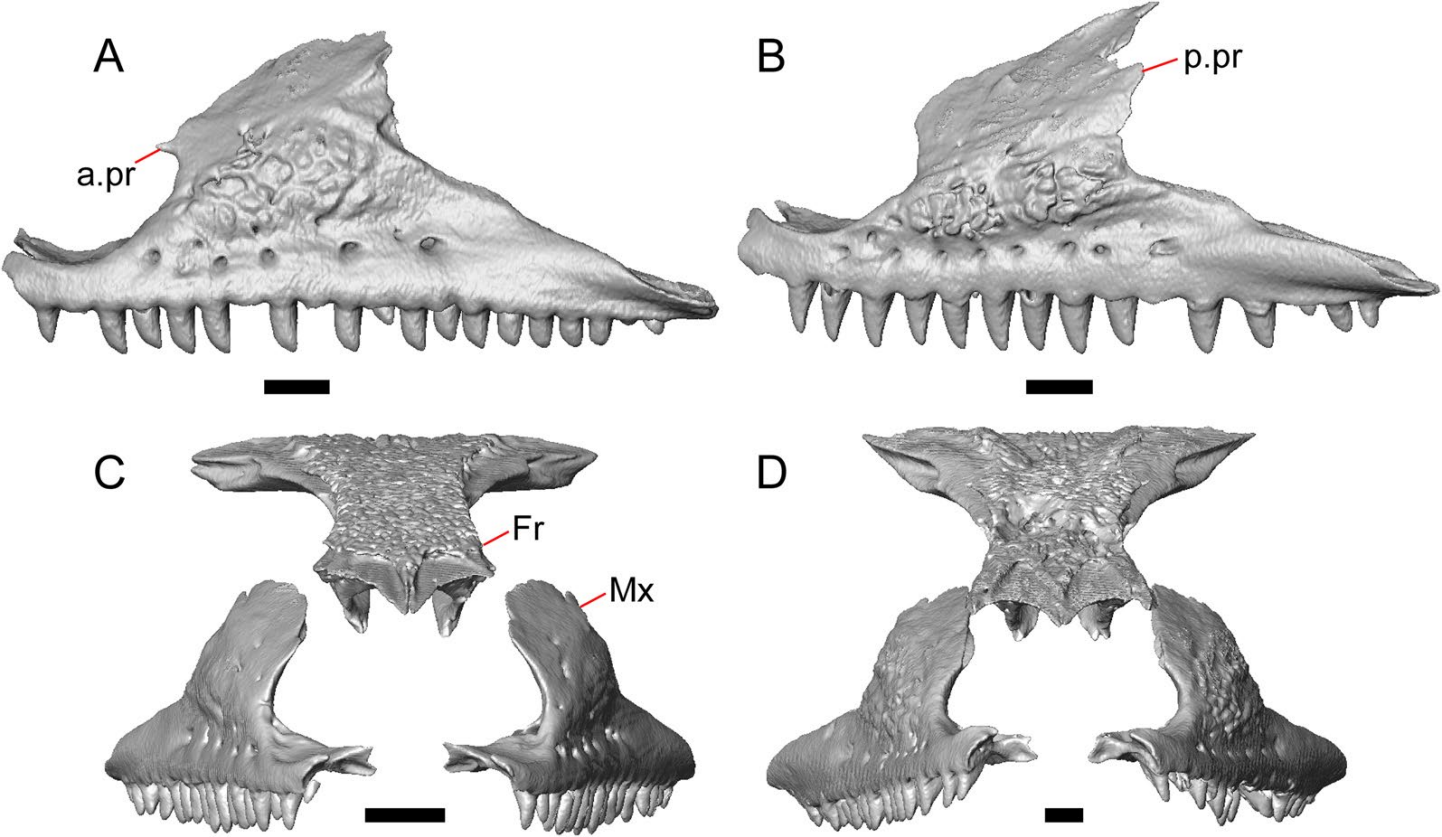
Maxillae and frontals. Scale bars = 1 mm. **a** Left maxilla of *E. multicarinata* TNHC 35666 in lateral view. **b** Left maxilla of *A. mixteca* UTA 30324 in lateral view. **c** Maxillae and frontal of *E. coerulea* TNHC 58792 in anterior view. **d** Maxillae and frontal of *G. infernalis* TNHC 18988 in anterior view. *a.pr* anterior process, *Fr* frontal, *Mx* maxilla, *p.pr* posterior process

17. Distinct anteriorly-facing projection on the anterodorsal face of the facial process of the maxilla (Ledesma et al. 2021 [14]): 0 = present (Fig. 22a); 1 = absent (Fig. 22b).

#### Remarks.

An anterodorsal projection of the facial process is present in most specimens of *Elgaria* (excluding *E. cedrosensis*), *G. lugoi*, *G. ophiurus* TCWC 35604, *Barisia* (excluding *B. imbricata* TNHC 76984), *A. campbelli* (although both specimens are bilaterally asymmetric), *A. taeniata*, *A. gadovii*, *A. monticola* TNHC 32083, and *A. moreletti* TNHC 29675. The projection is absent in other *Abronia* and in *G. infernalis*, *G. liocephalus*, and *G. parvus*.

**Fig. 23:**
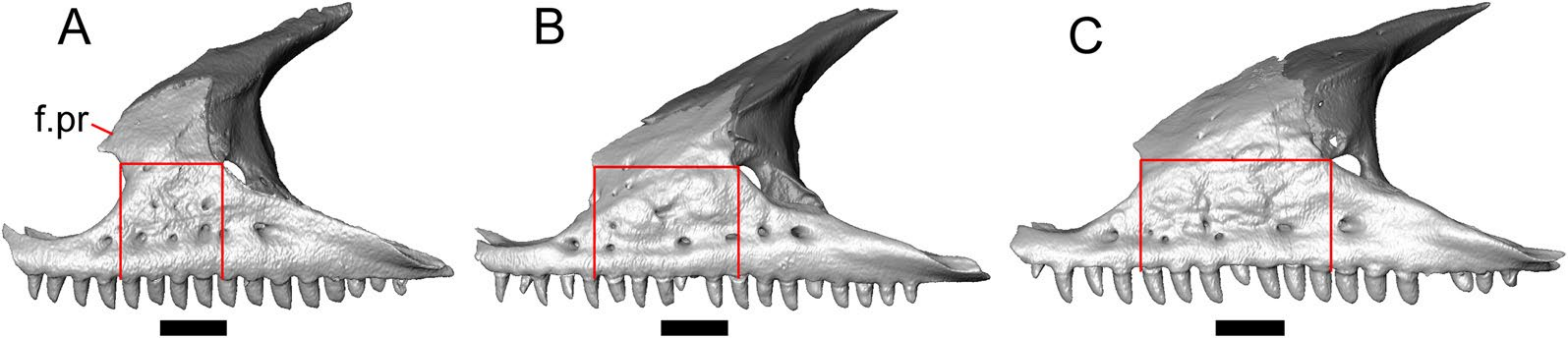
Relative width of the facial process of the left maxilla, marked by red lines. Left prefrontal shown to emphasize the anterior margin of the lacrimal foramen. Scale bars = 1 mm. **a** *A. monticola* TNHC 32083. **b** *E. cedrosensis* SDNHM 27702. **c** *G. lugoi* LACM 116254. *f.pr* facial process

18. Relative width of the facial process of the maxilla (left maxilla), measured in tooth positions spanning the facial process divided by the total number of tooth positions (modified from Good 1988 [38]). The posterior margin of the facial process was marked at the anterior margin of the lateral exposure of the lacrimal foramen, and the anterior margin was marked at the end of a line drawn from the posterior margin that is perpendicular to the horizontal access of the maxilla. The number of teeth under that line was counted and divided by the total number of tooth positions: 0 =  < 0.305 (narrow; Fig. 23a); 1 = 0.305–0.375 (intermediate; Fig. 23b); 2 =  > 0.375 (wide; Fig. 23c).

#### Remarks.

A narrow facial process is distinctive of species previously assigned to *Mesaspis* and was previously considered to be derived in *Mesaspis* and *Abronia* [38]. However, it is not straightforward to standardize the posterior and anterior points at which that narrowness is perceived and measured. We created a standardized line to examine the width of the facial process (see above). Instead of measuring the length of the line, we counted the number of tooth positions present under the line, and divided that number by the total number of tooth positions. Measuring the relative width of the facial process using tooth count is easier to score for fossils in which the premaxillary and/or orbital processes of the maxilla are warped or broken (both of which are frequent occurrences in our experience), and removes controversy over which length to measure (i.e., maximum length, length perpendicular to the tooth row, length of the tooth row, etc.). Incomplete tooth positions of 0.5 or over were rounded to the next whole number (e.g., if the facial process was 3.5 tooth positions wide, that value was rounded up to 4 tooth positions). The data were binned and assigned to three states of equal length with the function ‘bin’ with the ‘length’ method, as in Character 3.

Taxa and specimens that are scored as having a narrow facial process include *A. campbelli*, *A. lythrochila* TNHC 112900, *A. gadovii*, *A. moreletti* TNHC 29675, *A. monticola* TNHC 32083, *G. parvus* SRSU 5538, *E. panamintina* MVZ 191076, and *E. velazquezi* SDNHM 68678. A wide facial process is present in the anguid outgroups and *G. lugoi* LACM 116254. All other gerrhonotines exhibit the intermediate state.

**Fig. 24:**
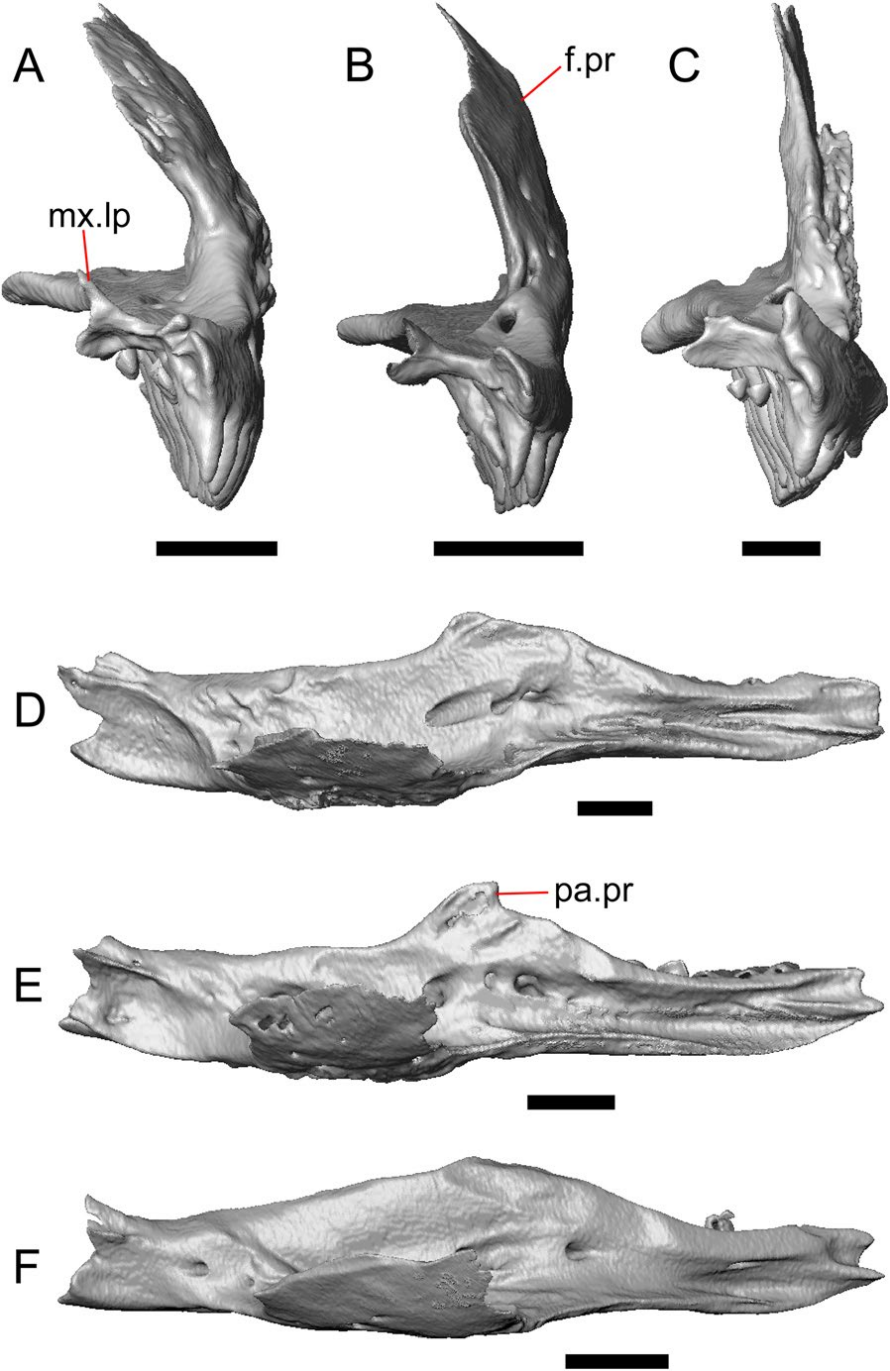
Left maxillae. Scale bars = 1 mm. **a** *A. campbelli* UTA 35945 in anterior view. **b** *E. cedrosensis* SDNHM 27702 in anterior view. **c** *B. levicollis* MVZ 68783 in anterior view. **d** *E. multicarinata* TNHC 35666 in dorsal view. **e** *A. campbelli* UTA 35945 in dorsal view. **f** *G. lugoi* LACM 116254 in dorsal view. *f.pr* facial process, *mx.lp* maxillary lappett, *pa.pr* palatine process

19. Medial inflection of the facial process (new): 0 = present (Fig. 24a, b); 1 = absent; the facial process faces almost straight dorsally (Fig. 24c).

#### Remarks.

State 0 accommodates a wide range of variation, especially among *Abronia* and *Elgaria*. Some species have a distinct inflection point creating a dorsal lamina (e.g., *A. campbelli*), while others have a relatively uniform curvature (e.g., *E. cedrosensis*). State 1 is restricted to *Barisia levicollis*.

20. Large posterior projection on the posterior edge of the facial process of the maxilla between the posterodorsal apex and the posteroventral margin of the process; the latter is defined by the anterior margin of the lacrimal foramen in lateral view (modified from Good 1987 [12], character 16): 0 = absent (Fig. 22a); 1 = present (Fig. 22b).

#### Remarks.

A projection ventral to the apex of the dorsal lamina is present in *A. mixteca* and *G. ophiurus* TCWC 35604. This character accommodates the morphology of the maxilla-prefrontal suture that was previously described by Good [12] as a “lop-sided W pattern.” We did not examine the other species of *Abronia* that were described as having that pattern (*A. oaxacae* and *A. deppii*).

21. Morphology of the palatine process of the maxilla (modified from Good 1987 [12], character 22): 0 = palatine process has a well-developed medial projection (Fig. 24d); 1 = palatine process has a well-developed medial projection that faces posteriorly (Fig. 24e); 2 = palatine process lacks a discrete medial projection (Fig. 24f).

#### Remarks.

*Gerrhonotus lugoi* is distinctive in lacking a discrete projection of the palatine process of the maxilla. A posterior-facing projection from the palatine process is present in *A. campbelli*, *A. monticola* TNHC 32083, and *E. velazquezi* SDNHM 68678.

22. Contact of the maxilla and the frontal (Good 1987 [12], character 13): 0 = absent (Fig. 22c); 1 = present (Fig. 22d).

#### Remarks.

Osteoderms fused to the skull may prevent accurate scoring of this character on traditionally prepared skulls. Contact is best observed from CT scans that have been digitally disarticulated to show only the maxilla and the frontal. Contact was observed in some species of *Abronia* (e.g., *A. lythrochila* TNHC 112900, *A. campbelli*) and *Gerrhonotus* (e.g., *G. infernalis*, *G. lugoi*).

23. Maxillary lappet located between the premaxilla and the vomer (*Invariant*; modified from Good 1987 [12], character 21): 0 = present (Fig. 22a), 1 = absent.

#### Remarks.

The maxillary lappet is present in all anguids, but is somewhat shorter compared to other gerrhonotines in *Barisia*, *A. mixteca*, *G. infernalis*, *G. lugoi*, and *G. ophiurus* TCWC 35604 [14].

### Lacrimal

**Fig. 25:**
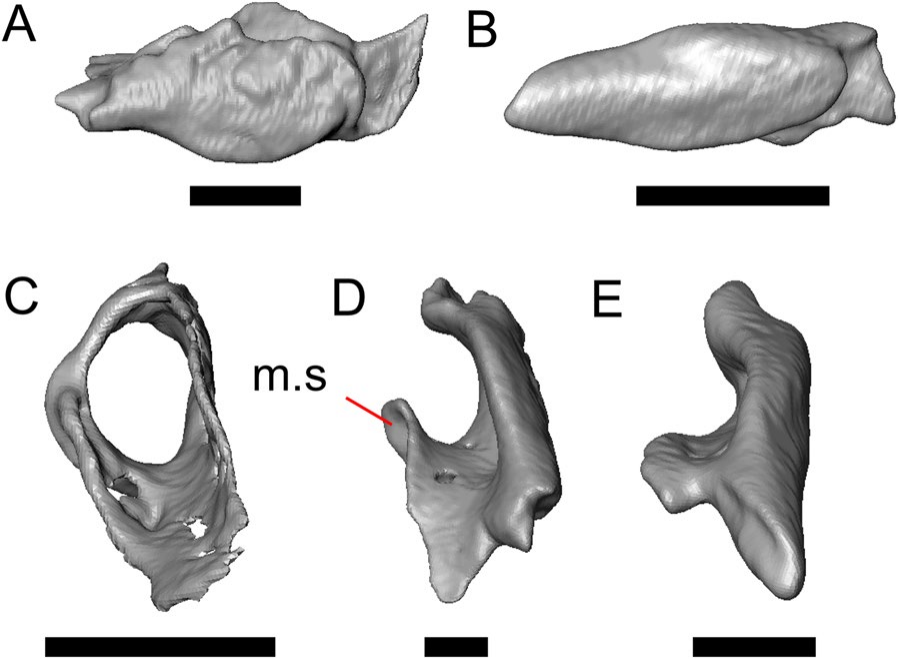
Right lacrimals. For **a**, **b** scale bars = 1 mm, for **c**–**e** scale bars = 0.5 mm. **a** *B. levicollis* MVZ 68783 in lateral view. **b** *E. paucicarinata* SDNHM 45100 in lateral view. **c** *G. parvus* SRSU 5537 in posterior view. **d** *B. levicollis* MVZ 68783 in posterior view. **e** *E. paucicarinata* SDNHM 45100 in posterior view. *m.s* medial shelf

24. Sculpturing on the lateral surface of the lacrimal (modified from Bhullar 2011 [60], character 116): 0 = absent (Fig. 25b); 1 = present (Fig. 25a).

#### Remarks.

Sculpturing on the lateral surface of the lacrimal is present in *A. campbelli* UTA 35945, *A. mixteca* UTA 30324, *A. lythrochila* TNHC 112900, *A. taeniata*, *Barisia*, *G. infernalis* TNHC 18988, *G. ophiurus* TCWC 35604, and *E. panamintina* MVZ 75918.

25. Contribution of the medial shelf of the lacrimal to the lacrimal foramen (Ledesma et al. [14]): 0 = the shelf faces medially, contributing ventrally to the lacrimal foramen (Fig. 25e); 1 = the shelf curves dorsomedially, increasing the medial contribution of the lacrimal to the lacrimal foramen (Fig. 25d); 2 = the medial shelf and dorsal face of the lacrimal are connected, completely enclosing the lacrimal foramen (Fig. 25c).

#### Remarks.

The medial shelf of the lacrimal contributes both medially and ventrally to the lacrimal foramen in most gerrhonotines. In *G. parvus* SRSU 5537, the medial shelf extends dorsally to fuse with the lateral wall of the lacrimal, completely enclosing the lacrimal foramen. The medial shelf contributes only ventrally to the lacrimal foramen in *E. paucicarinata*, *G. infernalis* TNHC 18988, *G. lugoi* LACM 116254, and *A. monticola*.

### Prefrontal

**Fig. 26:**
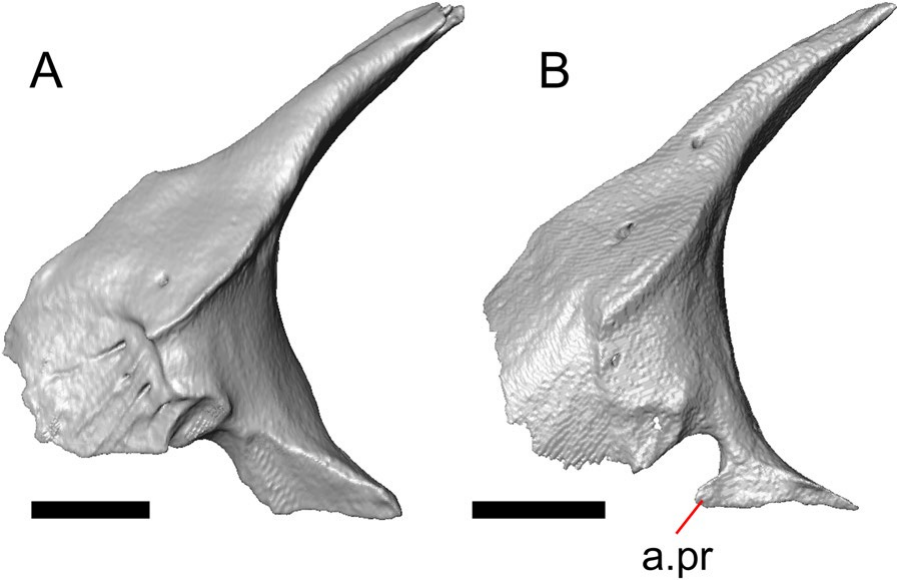
Left prefrontals in lateral view. Scale bars = 1 mm. **a** *E. coerulea* TNHC 14643. **b.** *G. lugoi* LACM 116254. *a.pr* anterior process

26. Anterior projection of the posteroventral process of the prefrontal (Ledesma et al. 2021 [14]): 0 = present (Fig. 26b); 1 = absent (Fig. 26a).

#### Remarks.

A distinct anterior projection of the posteroventral process of the prefrontal is present in *A. campbelli*, *A. lythrochila* TNHC 112900, *A. mixteca* UTA 30324, *A. ornelasi* UTA R-6220, *A. gadovii* TCWC 9907, and *Gerrhonotus* (except in *G. ophiurus* TCWC 35604). The projection is absent in *Barisia* and in most species of *Elgaria*, but is present in both specimens of *E. cedrosensis* and *E. paucicarinata*. Several specimens have a projection on one side but not the other (e.g., *A. lythrochila* TNHC 112900, *E. kingii* SDNHM 27895).

### Jugal

**Fig. 27:**
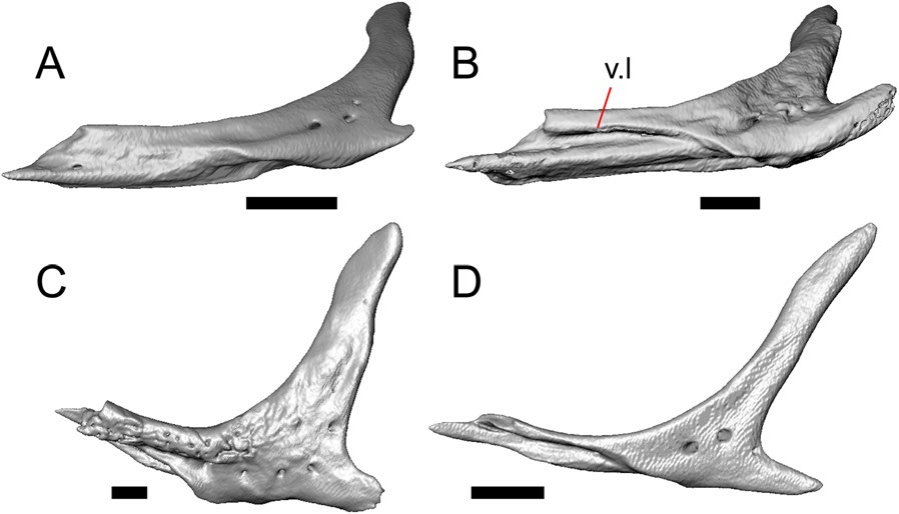
Left jugals. Scale bars = 1 mm. **a** *E. cedrosensis* SDNHM 27702 in ventrolateral view. **b** *A. lythrochila* TNHC 112900 in ventrolateral view. **c** *B. levicollis* MVZ 68782 in lateral view. **d** *A. ornelasi* UTA 6220 in lateral view. *v.l* ventral lamina

27. Sculpturing on the lateral surface of the jugal (modified from Conrad et al. 2011 [18], character 50): 0 = absent (Fig. 27b); 1 = present (Fig. 27a).

#### Remarks.

Most gerrhonotines lack sculpturing on the lateral surface of the jugal, but sculpturing is present in *B. levicollis* and *B. ciliaris* FMNH 30707.

28. Ventral lamina from the lateral surface of the jugal for articulation with the orbital process of the maxilla (new): 0 = absent (Fig. 27a); 1 = present (Fig. 27b).

#### Remarks.

An overhanging ventral lamina is present in many species of *Abronia*, *B. ciliaris* FMNH 30707, *B. levicollis* MVZ 68782, *E. paucicarinata* SDNHM 45106, *E. velazquezi* SDNHM 68677, *G. liocephalus* TCWC 8585, and *G. lugoi* LACM 116254.

29. Jugal spur (quadratojugal process) (Gauthier et al. 1988 [57], character 11): 0 = absent; 1 = present (Fig. 27).

#### Remarks.

A jugal spur is present on the jugals of almost all anguids, but is absent on one of the jugals of *E. nana* SDNHM 52886, *E. panamintina* MVZ 191076, and *O. mimicus* NCSM 25699.

### Frontal

**Fig. 28:**
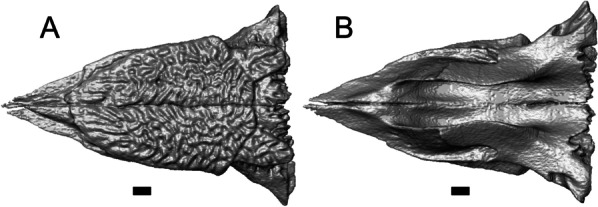
Frontal of *P. apodus* YPM 12870. Scale bars = 1 mm. **a** Dorsal view. **b** Ventral view

**Fig. 29:**
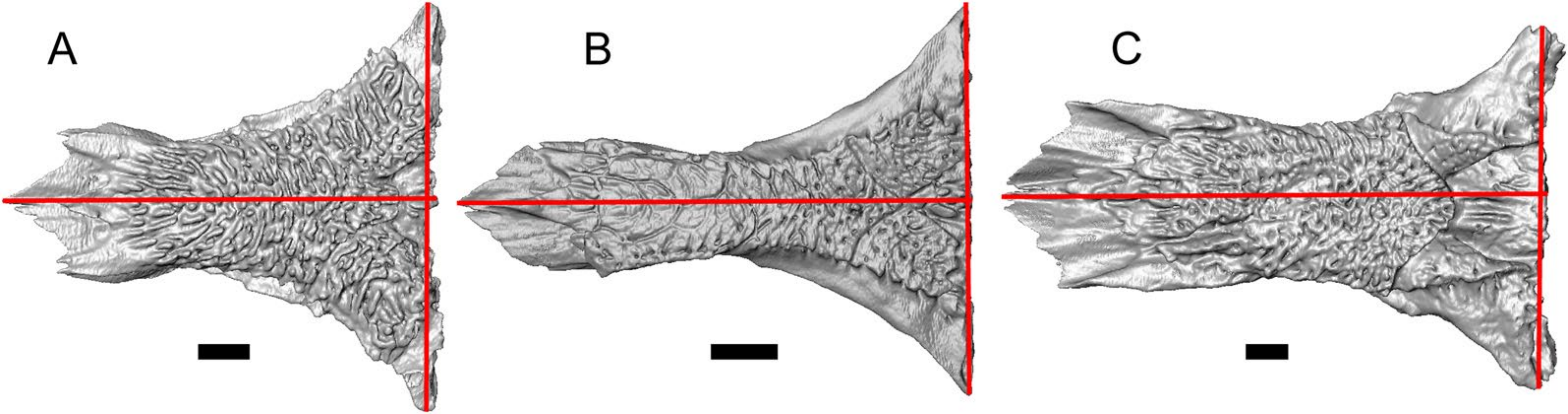
Frontals in dorsal view illustrating length and width measurements (red lines). Scale bars = 1 mm. **a** *A. campbelli* UTA 35945. **b** *E. cedrosensis* SDNHM 27702. **c** *B. levicollis* MVZ 68782

30. Postnatal fusion of the frontal bones (Gauthier 1982 [28], character 91): 0 = frontal bones unfused and present as two separate elements (Fig. 28); 1 = frontal bones fused into a single element (Fig. 29).

#### Remarks.

The frontals are fused early in postnatal development in gerrhonotines [19], but remain unfused throughout ontogeny in diploglossines and all extant anguines.

31. Frontal proportions, ratio of maximum length (anterior tip to posterior margin) to maximum width (at the posterior end of the element) (adapted from Wilson 1968 [3]): 0 =  < 1.22 (relatively broad; Fig. 29a); 1 = 1.22–1.39 (intermediate; Fig. 29b); 2 =  > 1.39 (relatively narrow; Fig. 29c).

#### Remarks.

*Abronia* (excluding *Mesaspis*) was previously described as possessing a less elongate frontal compared to other gerrhonotines [3]. We standardized that qualitative observation by taking measurements that we then binned into discrete morphological character states.

We took continuous measurements of the maximum width and the maximum length of the frontal and then divided the length by the width for each specimen to assign a single value to address frontal proportions. The data were binned and assigned to three states of equal length using the function ‘bin’ with the ‘length’ method, as in Character 3 and Character 18. We again emphasize that the values described here are specific to this analysis and the character states would need to be re-assessed should new specimens be scored for this matrix.

Based on the binning results, several *Abronia* (e.g., *A. campbelli*, *A. mixteca*) have wide frontals relative to other gerrhonotines. The frontals of other *Abronia* (*A. taeniata* TCWC 4911, *A. gadovii*, *A. moreletti* TNHC 29675, *A. monticola* TNHC 32083), most *Elgaria*, and most *Gerrhonotus* have intermediate ratios. The frontals of *Barisia*, *G. lugoi* LACM 116254, *G. infernalis* TNHC 18988, *E. velazquezi*, *E. paucicarinata* SDNHM 45100, and the anguid outgroups are relatively narrow compared to their length.

32. Interorbital constriction relative to both the anterior and posterior portions of the bone (modified from Conrad et al. 2011 [18], character 58; Gauthier 1982 [28], characters 21 and 91; Estes et al. 1988 [59], character 7): 0 = absent (Fig. 29); 1 = present (Fig. 28b).

#### Remarks.

The frontals of gerrhonotines were previously characterized as ‘hour-glass’ shaped due to constriction between the orbits [28, 66]. Interorbital constriction relative to both the anterior and posterior sections of the bone is present in gerrhonotines as previously reported, but we note that marked interorbital constriction occurs in members of many squamate clades, including varanids, xenosaurids, iguanians, and scincomorphs [19]. There is substantial variation in the degree of interorbital constriction among gerrhonotines (Fig. 29). The interorbital region is constricted in *C. enneagrammus* FMNH 108860 to a degree comparable to some gerrhonotines (e.g., *Barisia*). The interorbital region of examined anguines is constricted relative to the posterior face of the bone only, but the anterior portion of the bone tapers to a point; *P. apodus* YPM 12870 and *O. mimicus* NCSM 25699 were scored as state 0.

### Parietal

**Fig. 30:**
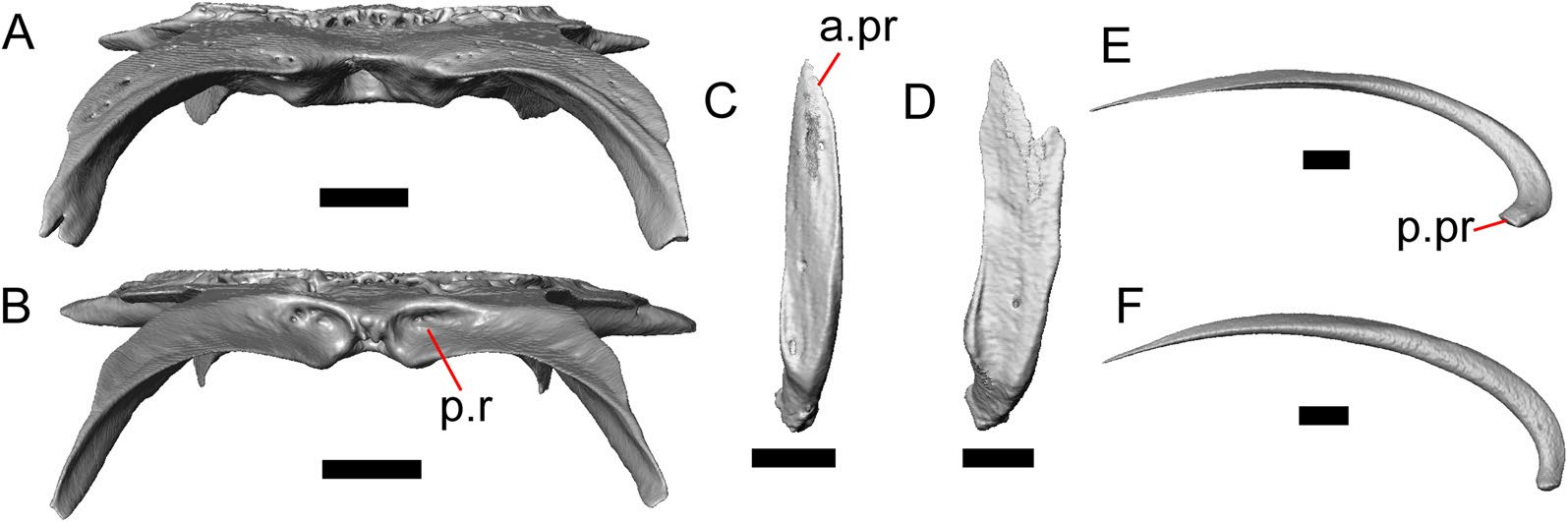
Parietals, supratemporals, and squamosals. Scale bars = 1 mm. **a** Parietal of *E. paucicarinata* SDNHM 45100 in posterior view. **b** Parietal of *E. cedrosensis* SDNHM 30296 in posterior view. **c** Right supratemporal of *E. velazquezi* SDNHM 68678 in dorsal view. **d** Right supratemporal of *A. campbelli* UTA 35945 in dorsal view. **e** Left squamosal of *E. multicarinata* TNHC 35666 in lateral view. **f** Left squamosal of *A. mixteca* UTA 30324 in lateral view. *a.pr* anterior process, *p.pr* posterior process, *p.r* posterior recess

33. Bilateral recess located on the posterior face of the parietal, medial to where the posterior surface meets the postparietal processes (Ledesma et al. 2021 [14]): 0 = absent (Fig. 30a); 1 = present (Fig. 30b).

#### Remarks.

The recess is present in *E. cedrosensis*, *E. velazquezi* SDNHM 68677, *E. multicarinata* TNHC 4478, *G. lugoi*, and the right side of the parietal of *G. infernalis* TNHC 92262. Juvenile *E. multicarinata* always lack a bilateral recess [14].

### Postfrontal and postorbital

34. Condition of the postfrontal and postorbital elements (*Invariant*; Conrad et al. 2011 [18], character 94; Evans 2008 [63]): 0 = separate postorbital and postfrontal bones are present; 1 = a single element (usually referred to as the postorbitofrontal) is present.

#### Remarks.

All gerrhonotines have separate postorbital and postfrontal elements in the posterior orbital region, but some diploglossines were reported previously to have a single element called the postorbitofrontal [63]. In squamates it is generally assumed that the postorbitofrontal represents a fusion between the postorbital and postfrontal, but we do not discount the possibility that some taxa may simply lack either the postorbital or the postfrontal, and so the single element filling that portion of the orbital region does not represent a fusion. Examined CT-scanned anguines and *C. enneagrammus* FMNH 108860 have separate postorbital and postfrontal elements.

### Squamosal

35. Morphology of the posteroventral end of the squamosal (modified from Good 1987 [12]): 0 = posterior process of the squamosal curves anteriorly so that the posterior end of the bone faces anteriorly (Fig. 30e); 1 = posterior process of the squamosal does not curve, and the posterior end of the bone faces ventrally (Fig. 30f).

#### Remarks.

In almost all gerrhonotines, the posteroventral end of the squamosal curves anteriorly such that the terminus of the bone faces anteriorly. In *A. mixteca*, the posteroventral end of the bone faces ventrally (contra Good [12]).

36. Posterior mediolateral expansion of the squamosal (*Invariant*; Bhullar 2011 [60], character 164): 0 = present; 1 = absent (Fig. 30e, f).

#### Remarks.

A posterior mediolateral expansion of the squamosal is present in *Xenosaurus* and is absent in Anguidae.

### Supratemporal

37. Bifurcation of the anterior process of the supratemporal (new): 0 = absent (Fig. 30c); 1 = present (Fig. 30d).

#### Remarks.

The supratemporal has two anterior projections in *A. campbelli* and has a single projection in other gerrhonotines and in other examined anguids.

### Vomer

**Fig. 31:**
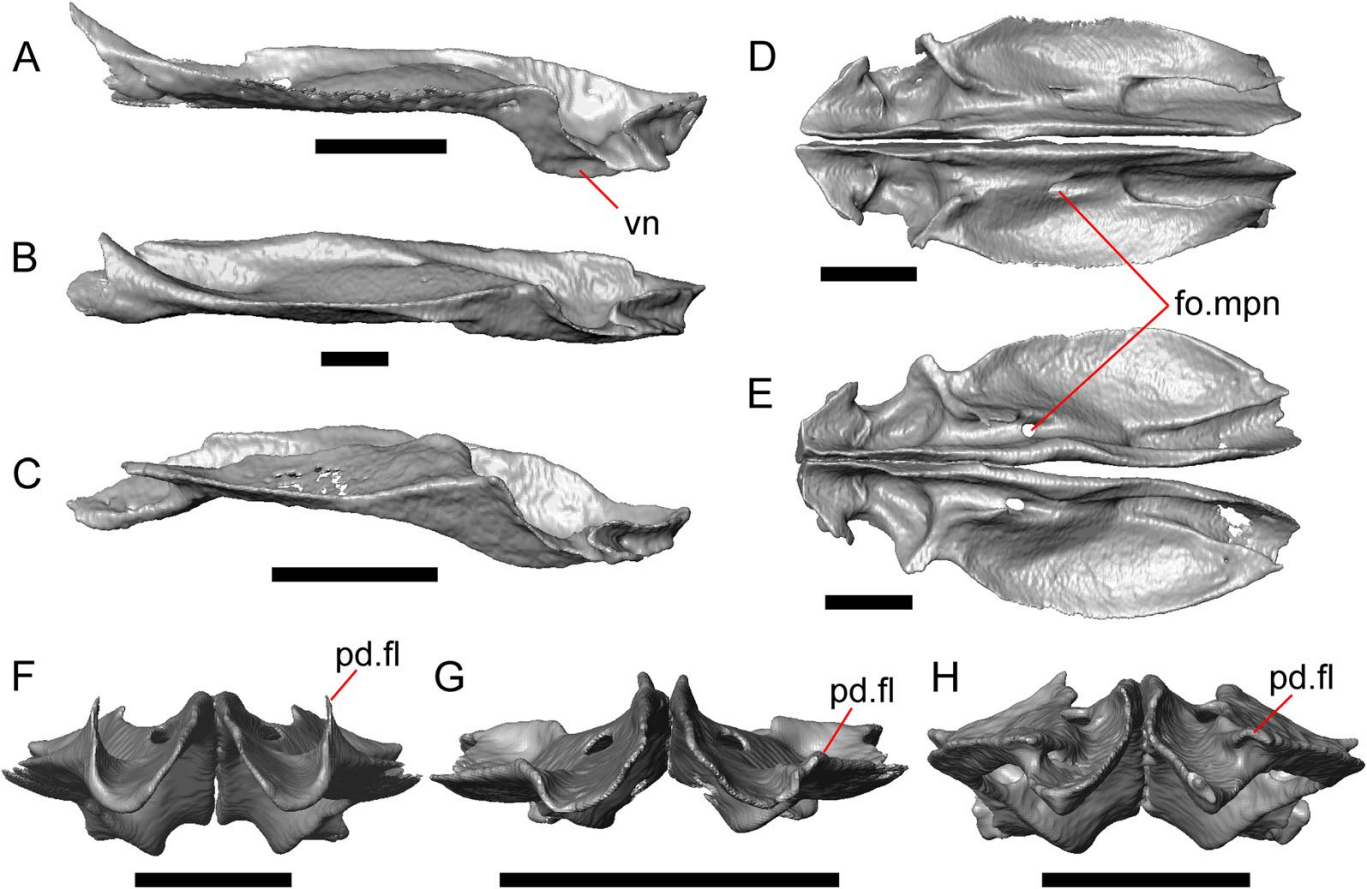
Vomers. Scale bars = 1 mm. **a** Right vomer of *A. campbelli* UTA 35945 in lateral view. **b** Right vomer of *G. infernalis* TNHC 18988 in lateral view. **c** Right vomer of *A. monticola* TNHC 32083 in lateral view. **d** Vomers of *E. paucicarinata* SDNHM 45100 in dorsal view. **e** Vomers of *A. taeniata* TCWC 4911 in dorsal view. **f** Vomers of *E. velazquezi* SDNHM 68678 in posterior view. **g** Vomers of *G. parvus* SRSU 5538 in posterior view. **h** Vomers of *A. monticola* TNHC 32083 in posterior view. *fo.mpn* foramen for the medial palatine nerve, *pd.fl* posterodorsal flange, *vn* vomeronasal region

38. Lamina on the posterodorsal surface of the vomer (Ledesma et al. 2021 [14]): 0 = high dorsal extent (Fig. 31f); 1 = low dorsal extent (Fig. 31g, h).

#### Remarks.

Most gerrhonotines possess a prominent posterodorsal lamina of the vomer. Some specimens (e.g., *A. mixteca* UTA 5790) have a slightly lower lamina that is scored as state 0*.* A low, posteriorly-facing lamina was observed in *A. monticola* TNHC 32083 (see character 44), which was scored as state 1. A lamina with a low dorsal extent is also present in *G. parvus* and *A. gadovii*.

39. Ventral extent of the vomeronasal region of the vomer relative to the posterior nasal region (new): 0 = vomeronasal region and posterior nasal region are at approximately the same dorsoventral level (Fig. 31b, c); 1 = vomeronasal region is ventral to the posterior nasal region (Fig. 31a).

#### Remarks.

This character is observed in lateral view and with respect to the horizontal axis of the isolated vomer, instead of with respect to the horizontal axis of the skull. In *A. campbelli*, *A. graminea*, *A. lythrochila* TNHC 112900, and *A. gadovii* TCWC 9907, the anterior vomeronasal region of the vomer is ventrally displaced from the posterior nasal portion of the element.

40. Orientation of the foramen for the medial palatine nerve on the vomer (Good 1987 [12], character 28): 0 = foramen penetrates the bone from a posterior angle and opens anteriorly on the ventral surface of the vomer (Fig. 31d); 1 = foramen penetrates the bone dorsally and exits ventrally (Fig. 31e).

#### Remarks.

The foramen for the medial palatine nerve was reported to penetrate the vomer dorsally in *Mesaspis* [12]. We also observed that morphology in species previously referred to *Mesaspis*, but it is also present in *A. campbelli*, *A. graminea*, *A. ornelasi* UTA R-6220, *A. taeniata*, *B. imbricata* TNHC 76894, *E. nana* SDNHM 17102, and *G. ophiurus* TCWC 35604. Several of those specimens have one state on one vomer and the other state on the other vomer. Those specimens, including both specimens of *A. campbelli*, *A. taeniata* TCWC 30660, *E. nana* SDNHM 17102, and *G. ophiurus* TCWC 35604, were scored as polymorphic.

### Palatine

**Fig. 32:**
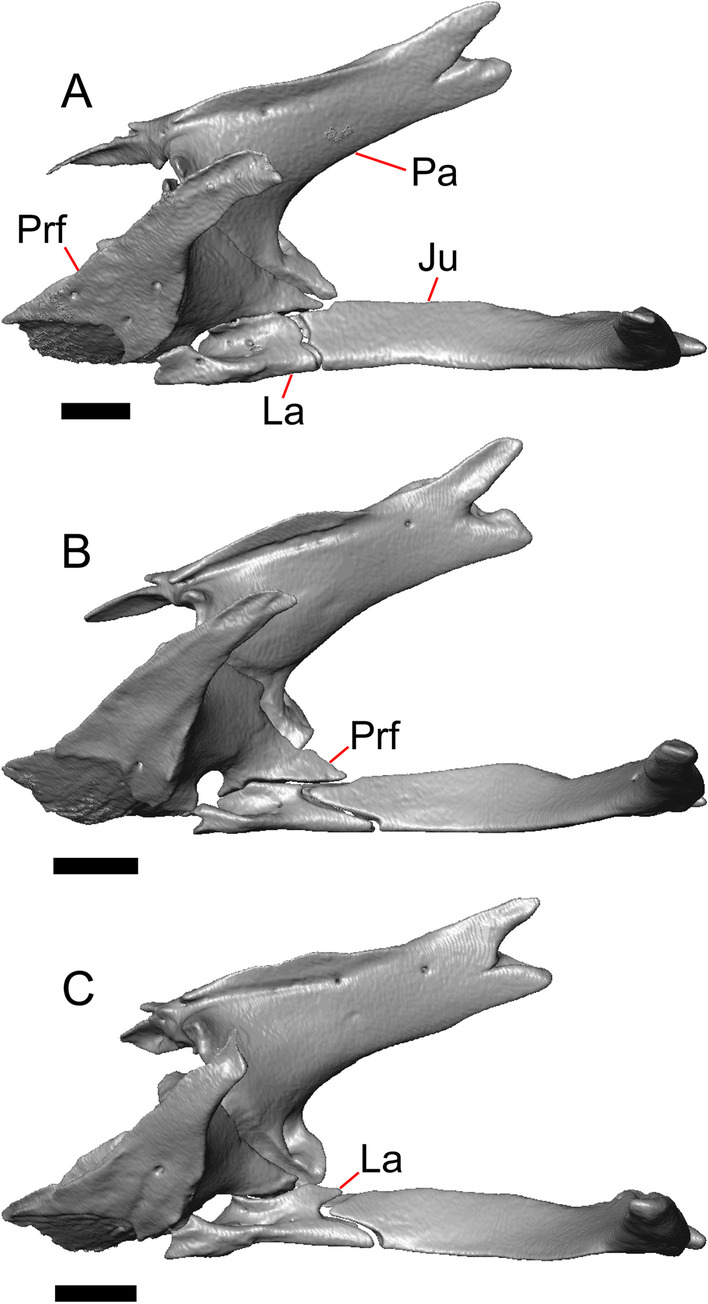
Palatine, lacrimal, jugal, and prefrontal in articulation and in dorsal view. Scale bars = 1 mm. **a** *E. multicarinata* TNHC 35666. **b** *E. paucicarinata* SDNHM 45106. **c** *E. kingii* SDNHM 24252. *Ju* jugal, *La* lacrimal, *Pa* Palatine, *Prf* prefrontal

41. Contact between the palatine and the jugal (Good 1987 [12], character 35): 0 = present (Fig. 32a); 1 = absent; excluded by the prefrontal (Fig. 32b); 2 = absent; excluded by the lacrimal (Fig. 32c).

#### Remarks.

In *E. kingii* SDNHM 24252, *C. enneagrammus*, and the left side of the skull of *E. coerulea* TNHC 58792, the lacrimal excludes contact between the palatine and the jugal. In other specimens in which the palatine and jugal do not contact, contact is excluded by the prefrontal. Among *Elgaria*, jugal-palatine contact is present in *E. multicarinata* and in *E. panamintina*. Contact is also present in *G. parvus*, *A. lythrochila*, *A. mixteca*, *A. ornelasi*, *B. levicollis*, and in *G. infernalis* TNHC 92262.

**Fig. 33:**
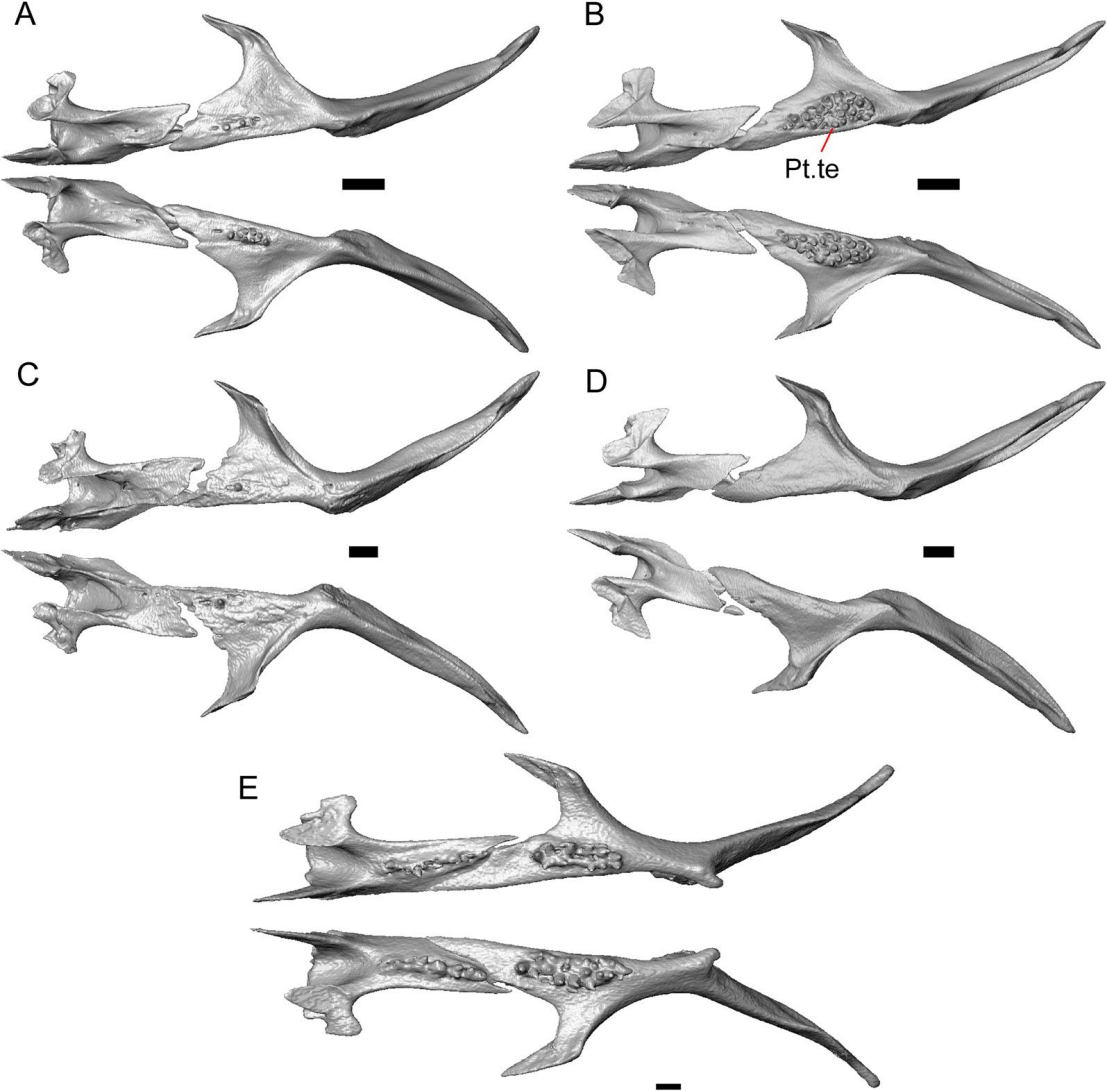
Palatines and pterygoids in articulation in ventral view. Scale bars = 1 mm. **a** *E. cedrosensis* SDNHM 27702. **b** *G. lugoi* LACM 116254. **c** *A. campbelli* UTA 35952. **d** *A. mixteca* UTA 30324. **e** *P. apodus* YPM 12870

42. Palatine teeth (Conrad et al. 2011 [18], character 115): 0 = absent (Fig. 33a–d); 1 = present (Fig. 33e).

#### Remarks.

Teeth on the ventral surface of the palatine are absent in all examined gerrhonotines and diploglossines, but are present in examined anguines.

**Fig. 34:**
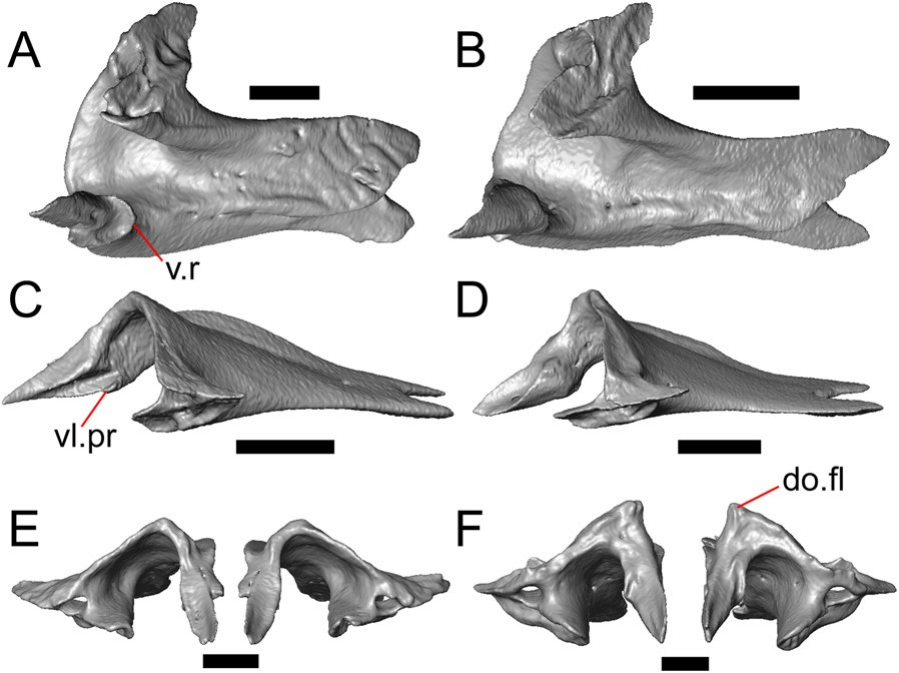
Palatines. Scale bars = 1 mm. **a** Left palatine of *E. panamintina* MVZ 75918 in anteroventral view (but figure oriented such that anterior is facing left). **b** Left palatine of *A. ornelasi* UTA 6220 in anteroventral view (but figure oriented such that anterior is facing left). **c** Left palatine of *A. monticola* TNHC 32083 in lateral view. **d** Left palatine of *A. ornelasi* UTA 6220 in lateral view. **e** Palatines of *E. panamintina* MVZ 75918 in anterior view. **f** Palatines of *B. levicollis* MVZ 68782 in anterior view. *do.fl* dorsal flange, *vl.pr* ventrolateral plate, *v.r* ventral ridge

43. Posteroventral ossification on the vomerine process of the palatine defining the posterior end of the articulation facet for the medial surface of the palatine process of the vomer (new): 0 = present (Fig. 34a); 1 = absent (Fig. 34b).

#### Remarks.

A posteroventral ossification marks the posterior end of the articulation facet for the vomer in almost all *Elgaria* and in *G. lugoi*, *G. parvus*, *G. ophiurus* TCWC 35604, *A. mixteca*, *A. campbelli* UTA 35952, and *A. gadovii*. The ridge is absent in *A. campbelli* UTA 35945, *A. graminea*, *A. taeniata*, *A. lythrochila* TNHC 112900, *A. ornelasi* UTA R-6220, *A. monticola* TNHC 32083, *A. moreletti*, *G. infernalis*, *G. liocephalus* TCWC 8585, and *E. panamintina* MVZ 191076.

44. Ventrolateral plate on the vomerine process of the palatine forming a concave articulation surface for the lateral surface of the palatine process of the vomer (new): 0 = absent (Fig. 34c); 1 = present (Fig. 34d).

#### Remarks.

In *A. moreletti* TNHC 29675 and *A. monticola* TNHC 32083, a ventrolateral plate creates a lateral canal on the vomerine process of the palatine. *Elgaria velazquezi* SDNHM 68677 and *E. panamintina* MVZ 191076 have a small lateral projection from the vomerine process that does not form a concave articulation surface; those species are scored as state 0. In *A. monticola* TNHC 32083, the posterior projection of the vomer faces posteriorly to insert into a concave articulation surface on the palatine.

45. Triangular dorsal flange of the palatine, located dorsal to the anterior margin of the choana (new). 0 = absent (Fig. 34e); 1 = present (Fig. 34f).

#### Remarks.

In most gerrhonotines there is a slight to a moderate thickening of the palatine dorsal to the anterodorsal portion of the choana. In several species, there is also a triangular flange above the choana. The flange is present and particularly large in *Barisia*, but is also present in *G. infernalis* TNHC 92262, *G. liocephalus* TCWC 8585, *E. cedrosensis* SDNHM 27702, and *E. paucicarinata* SDNHM 46106.

### Pterygoid

46. Pterygoid teeth (modified from Good 1987 [12], characters 91 and 92): 0 = completely absent (Fig. 33d); 1 = single row of teeth or tooth positions, including a highly reduced number of teeth (i.e., 1–3 teeth) (Fig. 33a, c); 2 = multiple rows of teeth or tooth positions (Fig. 33a, b, e).

#### Remarks.

Pterygoid teeth on the ventral surface of the palatal plate are present in *Elgaria* and *Gerrhonotus*, are absent in most *Barisia*, and are absent or reduced to 1–3 teeth in Abronia. Among *Abronia*, a few teeth are present on the pterygoids of *A. campbelli* UTA 95952, *A. lythrochila*, *A. gadovii*, *A. monticola*, and *A. moreletii*. The reduced pterygoid tooth count of those species of *Abronia* and *B. ciliaris* FMNH 30707 is similar to that of *E. cedrosensis* and *E. coerulea*, both of which have a single row of pterygoid teeth (although only on the left pterygoid of *E. cedrosensis* SDNHM 27702; see Fig. 33a). Most other *Elgaria* have multiple rows of teeth. *Elgaria panamintina* MVZ 75918 possesses multiple rows of unfilled tooth positions (i.e., all teeth either fell out or were in the process of being replaced) on the ventral surface of both pterygoids (state 2), and *G. ophiurus* TCWC 35604 has a single row of unfilled positions on the left pterygoid (state 1). The keeled-scale *Gerrhonotus* and *G. parvus* have a single row of pterygoid teeth, while *G. lugoi* has multiple rows of teeth.

**Fig. 35:**
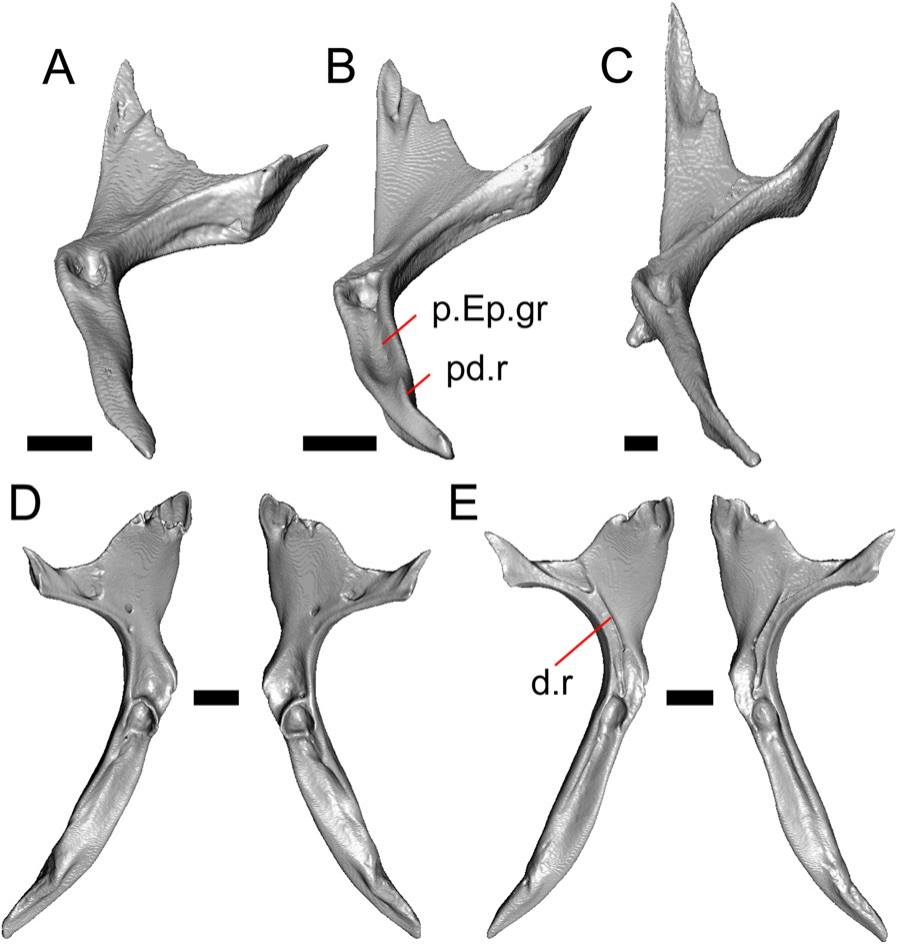
Pterygoids. Scale bars = 1 mm. **a** Right pterygoid of *A. campbelli* UTA 35945 in posterodorsal view. **b** Right pterygoid of *E. cedrosensis* SDNHM 27702 in posterodorsal view. **c** Right pterygoid of *P. apodus* YPM 12870 in posterodorsal view. **d** Pterygoids of *E. kingii* SDNHM 24252 in dorsal view (anterior is facing down). **e** Pterygoids of *E. paucicarinata* SDNHM 45106 in dorsal view (anterior is facing down). *d.r* dorsal ridge, *p.Ep.gr* postepipterygoid groove, *pd.*r posterodorsal ridge

47. Elongate, thin extension of the palatine process (palatal plate) of the pterygoid (new): 0 = absent (Fig. 35a, b); 1 = present (Fig. 35c).

#### Remarks.

An extension of the palatal process of the pterygoid is present in anguines and is absent in diploglossines and gerrhonotines.

48. Dorsal ridge on the pterygoid that is distinct at both its lateral and medial margins, located between the lateral-most edge of the pterygoid and the flattened palatal plate, beginning anterior to the fossa columella and running along the pterygoid flange to terminate at or slightly posterior to the ectopterygoid facet (Ledesma et al. 2021 [14]): 0 = absent (Fig. 35d); 1 = present (Fig. 35e).

#### Remarks.

A dorsal ridge between the pterygoid flange and the fossa columella is present in *E. panamintina*, *E. paucicarinata*, *E. velazquezi* SDNHM 68678, the left pterygoid of *E. kingii* SDNHM 27895, *G. liocephalus* TCWC 8585, and *A. lythrochila* TNHC 112900.

49. Postepipterygoid groove on the dorsal surface of the pterygoid (Good 1987 [12], character 40): 0 = deep (Fig. 35b); 1 = shallow (Fig. 35a); 2 = absent (Fig. 35c).

#### Remarks.

A shallow postepipterygoid groove is present in *A. campbelli*, *A. taeniata* TCWC 4911, and *A. moreletti* TNHC 29675. The groove is absent in *P. apodus* (both YPM 12870 and examined dry skeletal specimens) and examined dry skeletal specimens of *Anguis fragilis*. In the dry skeletal specimen *Ophisaurus ventralis* CAS 74926, the left pterygoid lacks a groove while the right side possesses a relatively deep groove.

50. Anterior–posterior ridge on the posterodorsal end of the quadrate process of the pterygoid (new): 0 = prominent (Fig. 35b); 1 = reduced (Fig. 35a).

#### Remarks.

A posterodorsal ridge on the quadrate process of the pterygoid is present in all gerrhonotines but is reduced in height (to the point of being nearly absent) in *A. campbelli* and in *A. taeniata*.

### Quadrate

**Fig. 36:**
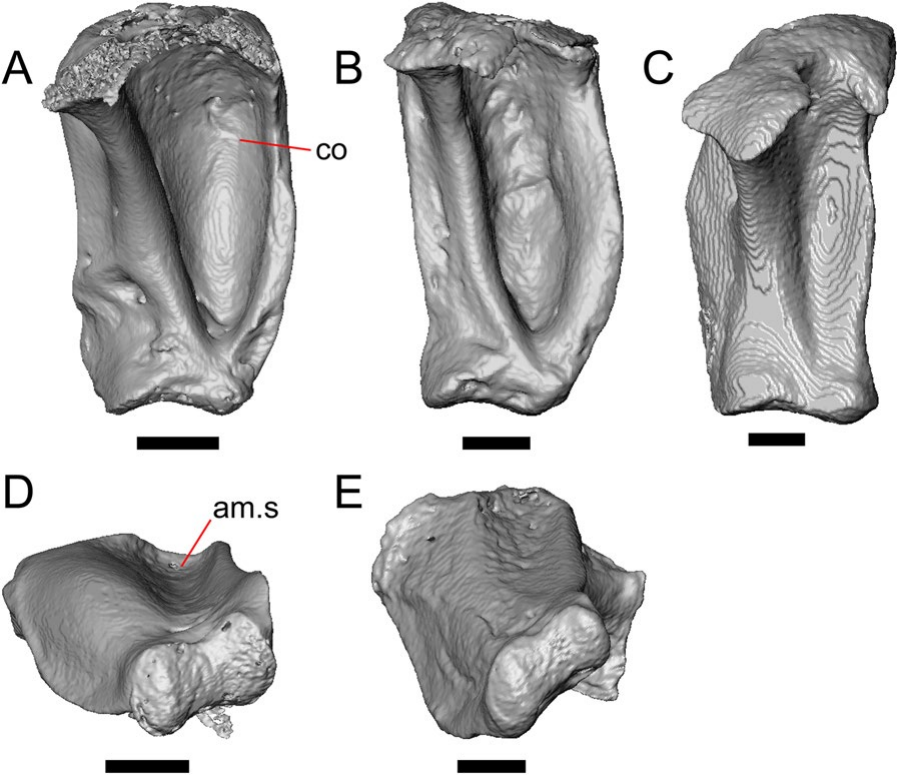
Right quadrates. Scale bars = 1 mm. **a** *E. paucicarinata* SDNHM 45100 in posterior view. **b** *A. campbelli* UTA 35952 in posterior view. **c** *P. apodus* YPM 12870 in posterior view. **d** *E. paucicarinata* SDNHM 45100 in ventral view. **e** *A. campbelli* UTA 35952 in ventral view. *am.s* anteromedial surface, *co* conch

51. Width of the conch of the quadrate in posterior view (new): 0 = dorsal portion of the conch is substantially wider than the ventral portion (Fig. 36a); 1 = dorsal portion of the conch is roughly the same width as the ventral portion (Fig. 36b).

#### Remarks.

The conch of the quadrate is markedly wider dorsally than ventrally in most examined gerrhonotines. The conch does not markedly widen dorsally relative to the ventral portion of the conch in *A. campbelli*, *A. lythrochila* TNHC 112900, *A. gadovii*, *B. ciliaris* FMNH 30707, and *B. levicollis* MVZ 68782. The conch of *P. apodus* YPM 12870 is poorly developed, so that specimen was scored as ‘?’ (Fig. 36c).

52. Anteromedial surface of the quadrate (new): 0 = anteromedial surface is distinctly concave (Fig. 36d); 1 = anteromedial surface is nearly flat (Fig. 36e).

#### Remarks.

The anteromedial surface of the quadrate is concave, especially dorsally, in most gerrhonotines and in other anguids. That surface is nearly flat in *A. campbelli* and *A. graminea* UTA 38831.

53. Dorsomedial narrowing of the quadrate (new): 0 = absent (Fig. 36a, b); 1 = present (Fig. 36c).

#### Remarks.

In Anguinae, the dorsomedial margin of the quadrate abruptly narrows such that the dorsal portion of the quadrate is considerably narrower in anterior and posterior view relative to the ventral and middle portions of the element.

### Sphenoid

**Fig. 37:**
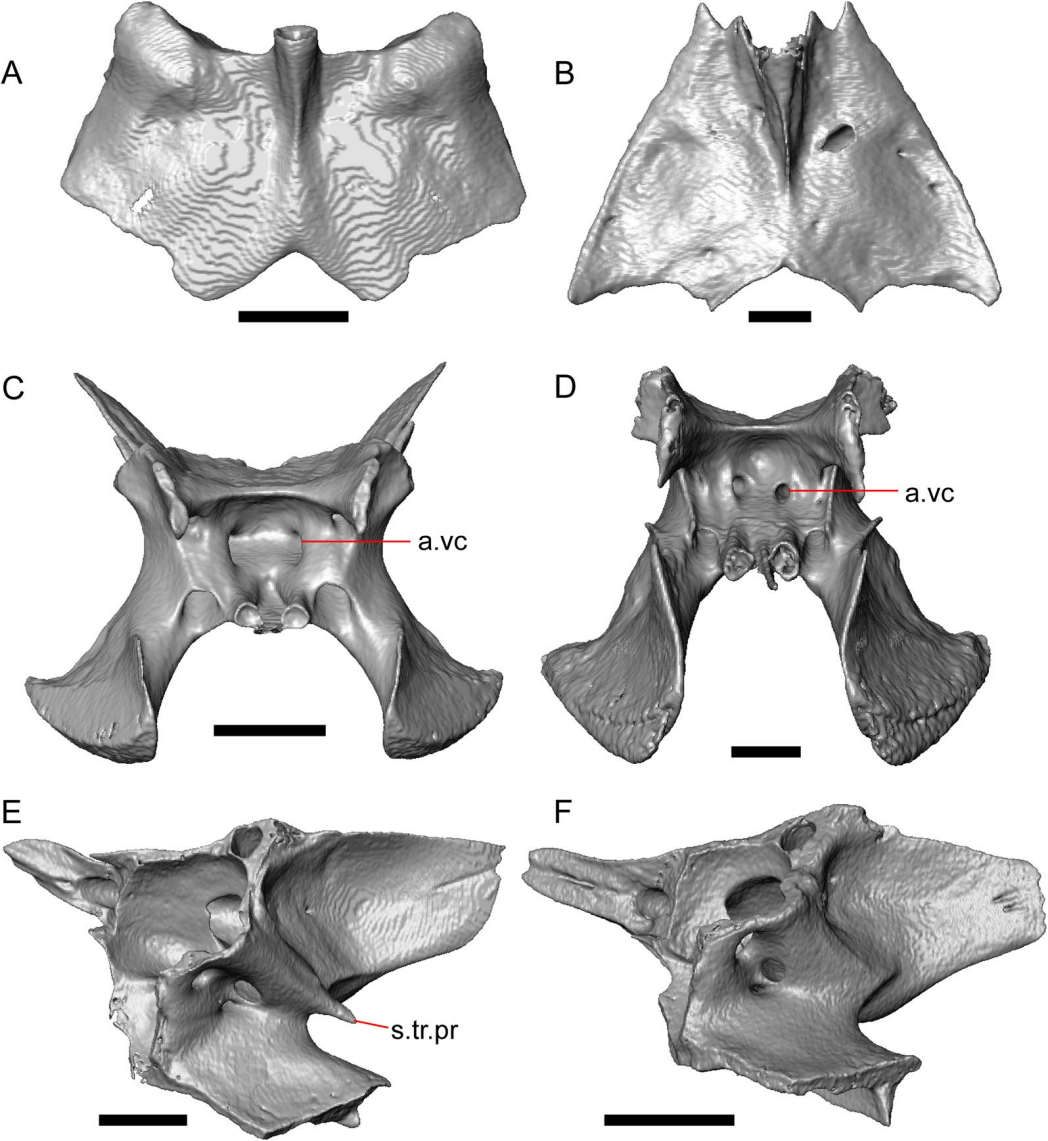
Supraoccipitals, sphenoids, and left prootics (individual braincase elements). Scale bars = 1 mm. **a** Supraoccipital of *E. coerulea* TNHC 14643 in dorsal view. **b** Supraoccipital of *G. infernalis* TNHC 18988 in dorsal view. **c** Sphenoid of *E. coerulea* TNHC 14643 in anterior view. **d** Sphenoid of *G. infernalis* TNHC 18988 in anterior view. **e.** Left prootic of *E. coerulea* TNHC 14643 in medial view. **f.** Left prootic of *A. graminea* UTA 38831 in medial view. *a.vc* anterior vidian canal, *s.tr.pr* supratrigeminal process of the prootic

54. Anterior opening of the internal carotid foramen (Ledesma et al. 2021 [14]): 0 = is directed anteromedially (Fig. 37c); 1 = is directed anteriorly (Fig. 37d).

#### Remarks.

The anterior opening of the internal carotid foramen is directed anteromedially in most gerrhonotines. In *G. infernalis* and *B. levicollis* MVZ 68782, the opening is directed anteriorly.

### Supraoccipital

55. Location of the lateral corner of the supraoccipital where it meets the otoocipital and the prootic, in dorsal view (Ledesma et al. 2021 [14]): 0 = lateral corner of the supraoccipital is positioned anterior to the posterior-most extent of the supraoccipital and the anterior face of the supraoccipital is wider than or a similar width to the posterior face (a roughly hexagonal supraoccipital in dorsal view) (Fig. 37a); 1 = lateral corner is positioned level or nearly level to the posterior-most extent of the supraoccipital where the bone forms a portion of the border of the foramen magnum, and the anterior portion of the dorsal surface of the supraoccipital is much narrower than the posterior portion (a roughly trapezoidal supraoccipital in dorsal view) (Fig. 37b).

#### Remarks.

Most gerrhonotines exhibit state 0. State 1 is present in *G. infernalis* and *B. levicollis*, although some skeletally mature dry specimens of *G. infernalis* exhibit state 0 [14]. In *A. taeniata* TCWC 4911, *A. gadovii* TCWC 11384, *G. liocephalus* TCWC 8585, and *G. ophiurus* TCWC 35604, the lateral corner is positioned closer to the posterior margin of the element than in most other examined gerrhonotines, but the supraoccipital still has a hexagonal shape and those specimens were scored as state 0.

### Prootic

56. Supratrigeminal process of the prootic (Estes et al. 1988 [59], character 50; Evans 2008 [63]): 0 = present (Fig. 37e); 1 = absent (Fig. 37f).

#### Remarks.

The supratrigeminal process is located on the medial surface of the prootic, and often bisects the incisura prootica in lateral view. Bilateral asymmetry is present in some specimens (e.g., *E. multicarinata* TNHC 35666) in which the process is moderately developed on one prootic but nearly undetectable on the other prootic. The supratrigeminal process is absent in *A. graminea*, *A. mixteca*, *Barisia*, *G. lugoi*, *G. infernalis*, and *G. parvus*.

### Otooccipital

**Fig. 38:**
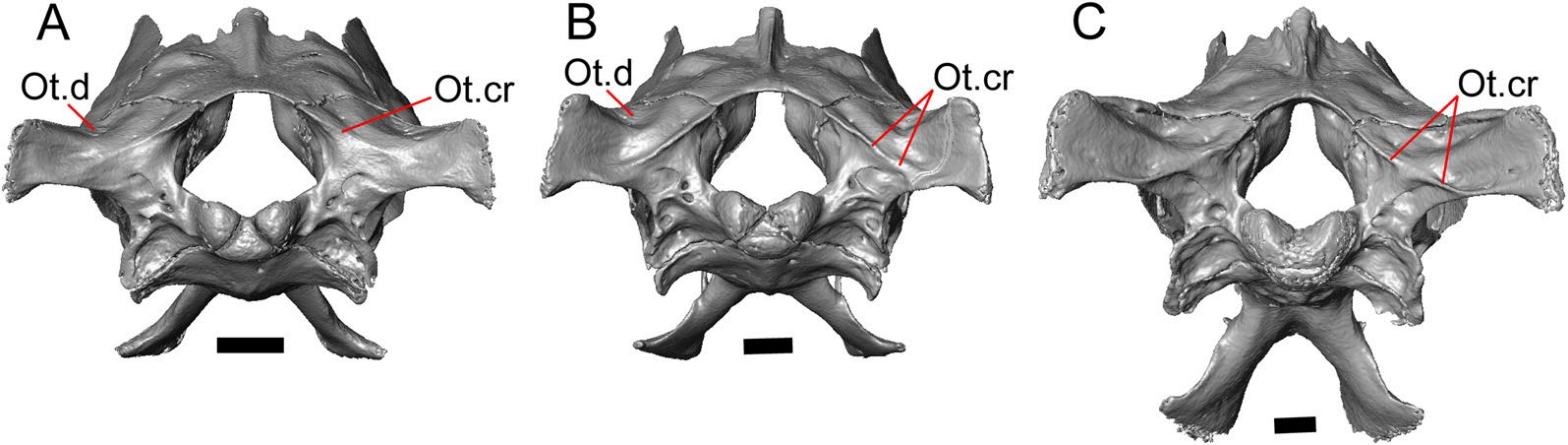
Braincases in posterior view. Scale bars = 1 mm. **a** *A. ornelasi* UTA 6220. **b** *E. panamintina* MVZ 191076. **c** *G. infernalis* TNHC 18988. *Ot.cr* otooccipital crest, *Ot.d* otooccipital depression

57. Excavation on the dorsal surface of the otooccipital anterior to the paroccipital process (Good 1987 [12], character 73): 0 = deep (Fig. 38b, c); 1 = shallow (Fig. 38a).

#### Remarks.

The dorsal excavation on the otooccipital is present on both otooccipitals in all specimens. *Abronia graminea*, *A. mixteca*, and *A. ornelasi* UTA R-6220 exhibit a shallow depression.

58. Crest extending from the posterior edge of the supraoccipital onto the posterior surface of the otooccipital (new): 0 = extends to the base of the paroccipital process (Fig. 38a); 1 = extends well onto the paroccipital process (Fig. 38c).

#### Remarks.

The posterior crest of the otoocipital extends well onto the paroccipital process in *G. infernalis*. On *B. levicollis* MVZ 68,782 and *E. panamintina* MVZ 191076 (Fig. 38b), the crest extends on to the paroccipital process on the left side and right side only, respectively. Those specimens are scored as polymorphic.

### Dentary

**Fig. 39:**
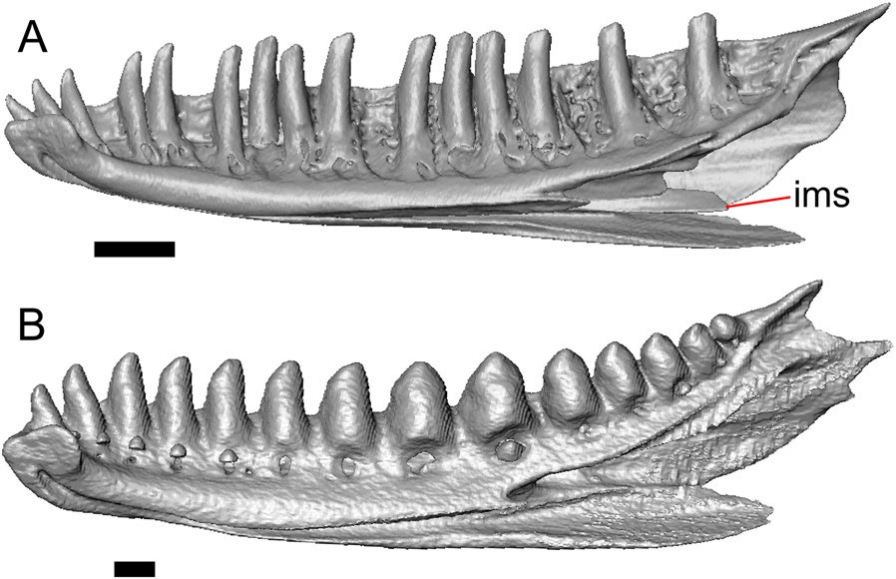
Right dentaries in medial view. Scale bars = 1 mm. **a** *E. paucicarinata* SDNHM 45100. **b** *P. apodus* YPM 12870. *ims* intramandibular septum

59. Free posteroventral margin of the intramandibular septum (Gauthier 1982 [28]; Meszoely 1970 [15]; Conrad et al. 2011 [18], character 171): 0 = absent (Fig. 39b); 1 = present (Fig. 39a).

#### Remarks.

A free posteroventral margin of the intramandibular septum was previously considered to be an apomorphy of all Anguidae [28] or Anguidae exclusive of *Diploglossus bilobatus* [18]. The free margin is present in all gerrhonotines and in *C. enneagrammus* FMNH 108860, but is absent in *O. mimicus* NCSM 25699 and *P. apodus* YPM 12870.

**Fig. 40:**
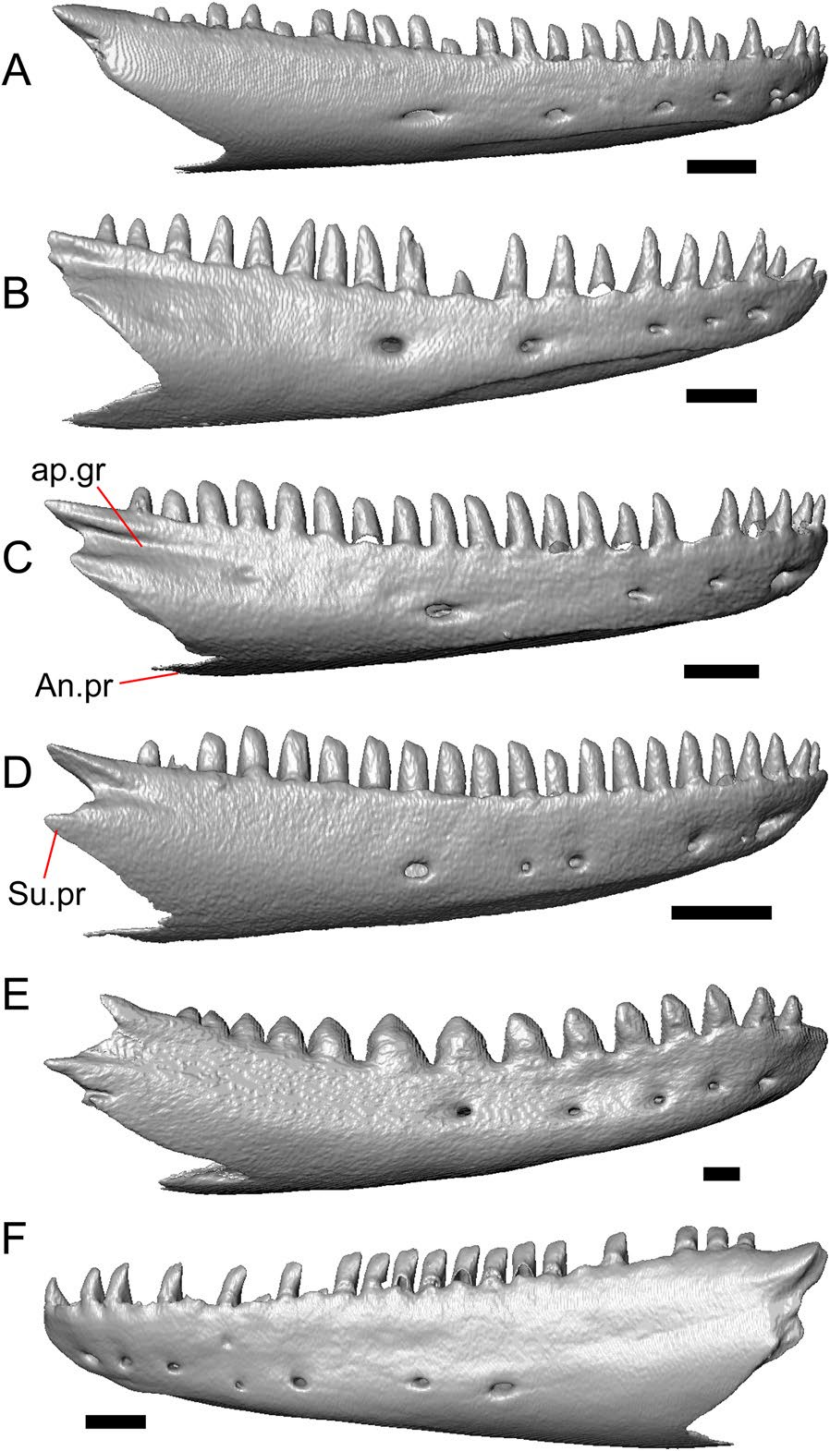
Right dentaries (except for **f**) in lateral view. Scale bars = 1 mm. **a** *E. velazquezi* SDNHM 68678. **b** *A. mixteca* UTA 5790. **c** *A. campbelli* UTA 35945. **d** *A. monticola* TNHC 32083. **e** *P. apodus* YPM 12870. **f** Left dentary of *E. velazquezi* SDNHM 68677. *An.pr* angular process, *ap.g*r anteroposterior groove, *Su.pr* surangular process

60. Position of the posterior end of the angular process of the dentary relative to the coronoid process of the dentary (modified from Gauthier 1982 [28]; Conrad et al. 2011 [18], character 186): 0 = the angular process of the dentary terminates anterior to the coronoid process of the dentary (Fig. 40a, c–f); 1 = the angular process of the dentary extends at or posterior to the coronoid process of the dentary (Fig. 40b).

#### Remarks.

This character is best scored on a disarticulated dentary. The angular process extends posterior to the coronoid process in *A. mixteca*, *A. gadovii* TCWC 9907, and *B. levicollis*.

The posterior extent of the angular process was framed relative to the extent of the surangular process by Gauthier [28] and Conrad et al. [18], and it was noted by Gauthier [28] that the surangular process is well-developed plesiomorphically in anguimorphs. Relatively few species of gerrhonotine have a distinct and well-developed surangular process (see character 61). We frame the relative length of the angular process relative to the coronoid process of the dentary, because the coronoid process is always present and well-developed in gerrhonotines.

61. Distinct surangular process of the dentary (modified from Gauthier 1982 [28]): 0 = present (Fig. 40c–f); 1 = absent (Fig. 40a, b).

#### Remarks.

A distinct suranguar process is absent in many gerrhonotines, but is present in *A. campbelli* UTA 35945, *A. graminea* UTA 38831, *A. taeniata*, *A. monticola* TNHC 32083, the right dentary of *A. gadovii* TCWC 9907, *B. imbricata* TNHC 76984, and *B. ciliaris* FMNH 30707. *Barisia levicollis*, most *Gerrhonotus*, and most *Elgaria* lack a distinct surangular process. Some specimens of *Elgaria* possess a small posterior strut of bone that articulates with the surangular (scored as state 1). The left dentaries of *E. panamintina* MVZ 191076 and *E. velazquezi* SDNHM 68677 (Fig. 40f) and the right dentary of *G. infernalis* TNHC 18988 possess a distinct process, and those specimens are scored as polymorphic (01). Anguines have a well-developed surangular process.

62. Anteroposterior groove (sublabial longitudinal groove) located ventral to the parapet on the posterolateral surface of the dentary (modified from Good 1987 [12], character 87): 0 = absent (Fig. 40a, d–f); 1 = present (Fig. 40b, c).

#### Remarks.

The presence of an anteroposterior groove was previously used to differentiate *Abronia* from other gerrhonotines (Good 1987). We observed the groove in many *Abronia* (exclusive of species previously assigned to *Mesaspis* and one dentary of *A. graminea* UTA 38831), *B. ciliaris* FMNH 30707, *B. imbricata* TNHC 76984, and *B. levicollis* MVZ 68783.

### Splenial

**Fig. 41:**
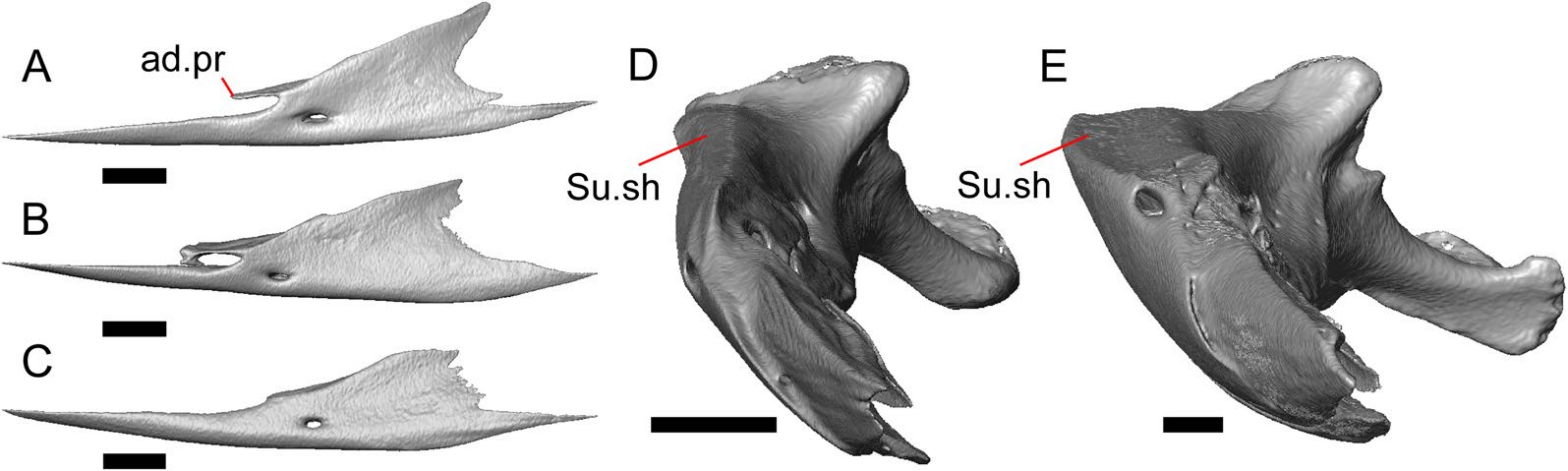
Right splenials (in medial view) and right surangular (in anterior view). Scale bars = 1 mm. **a** Splenial of *E. paucicarinata* SDNHM 45100. **b** Splenial of *E. kingii* SDNHM 27985. **c** *A. graminea* UTA 38831. **d** Surangular of *E. cedrosensis* SDNHM 27702. **e** *G. infernalis* TNHC 18988. *ad.pr* anterodorsal process, *Su.sh* surangular shelf

63. Anterodorsal projection of the splenial above the anterior inferior alveolar foramen (modified from Ledesma et al. 2021 [14]): 0 = present (Fig. 41a); 1 = present and ossifies ventrally to enclose the anterior inferior alveolar foramen (Fig. 41b); 2 = absent (Fig. 41c).

#### Remarks.

All gerrhonotines except for *A. graminea* have an anterodorsal projection of the splenial. The projection is also absent in anguines. The projection is present but relatively reduced in length in *B. imbricata* TNHC 76984 and *A. mixteca*. In *E. velazquezi* SDNHM 68678 and *E. kingii*, the projection extends ventrally to enclose the anterior inferior alveolar foramen, although only on the left splenial of *E. kingii* SDNHM 24252.

### Coronoid

**Fig. 42:**
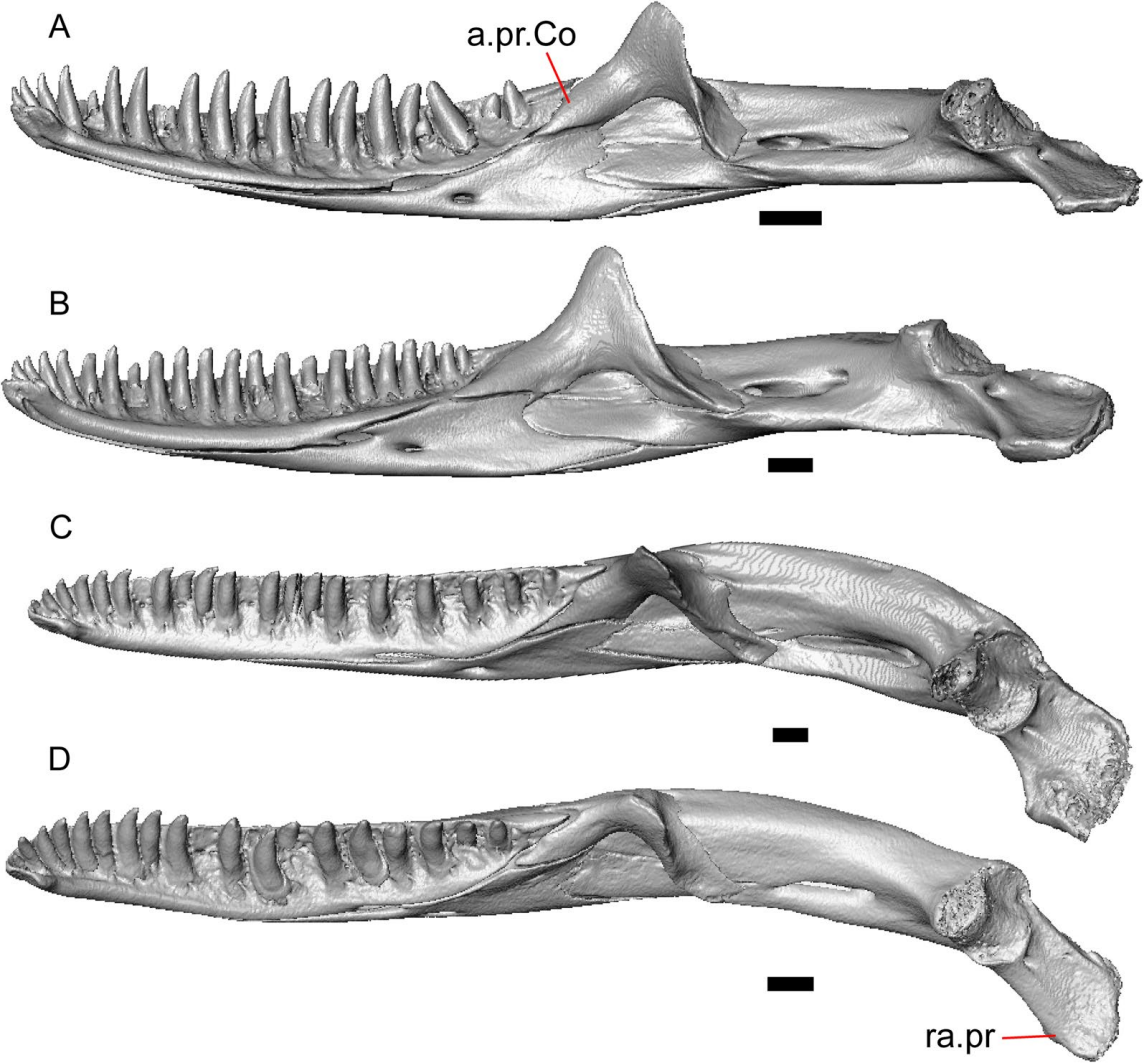
Right mandibles in medial view (**a**, **b**) and dorsomedial view (**c**, **d**). Scale bars = 1 mm. **a** *A. ornelasi* UTA 6220. **b** *E. nana* SDNHM 17102. **c** *G. infernalis* TNHC 92262. **d** *A. mixteca* UTA 30324. *a.pr.Co* anterior medial process of the coronoid, *ra.pr* retroarticular process

64. Extension of the medial exposure of the anterior medial process of the coronoid relative to the last tooth position on the dentary. This character is scored when the splenial is in articulation with the dentary (Good 1987 [12], character 86): 0 = process does not extend anterior to the last tooth position (Fig. 42a); 1 = process extends anterior to the last tooth position (Fig. 42b).

#### Remarks.

We also examined this character when the splenial was not in articulation, and found that without exception the anterior medial process of the coronoid extends far anterior to the last tooth position. In articulation, however, the medial exposure of the anterior medial process failed to extend past the last tooth position in *A. graminea*, *A. taeniata* TCWC 30660, *A. mixteca*, *A. ornelasi* UTA R-6220, and *A. gadovii*, paralleling the results of Good [12]. Additionally, the medial exposure of the anterior medial process failed to extend past the last tooth position in *G. parvus*, in which the process extended to, but not past, the last tooth position.

This character was somewhat ambiguous in *E. paucicarinata* SDNHM 45100 and *E. multicarinata* TNHC 4478. In those two specimens, one (but not both) of the dentaries lacked a distinct posteriormost tooth position where a position was present at the analogous location on the other dentary of those specimens. If a tooth position is truly absent, these specimens would be bilaterally asymmetric, but we scored the specimens as character state 1. *Elgaria nana* SDNHM 52886 is clearly bilaterally asymmetric; the left anterior medial process is close to, but does not extend past, the last tooth position. That specimen is scored as polymorphic (01).

### Surangular

**Fig. 43:**
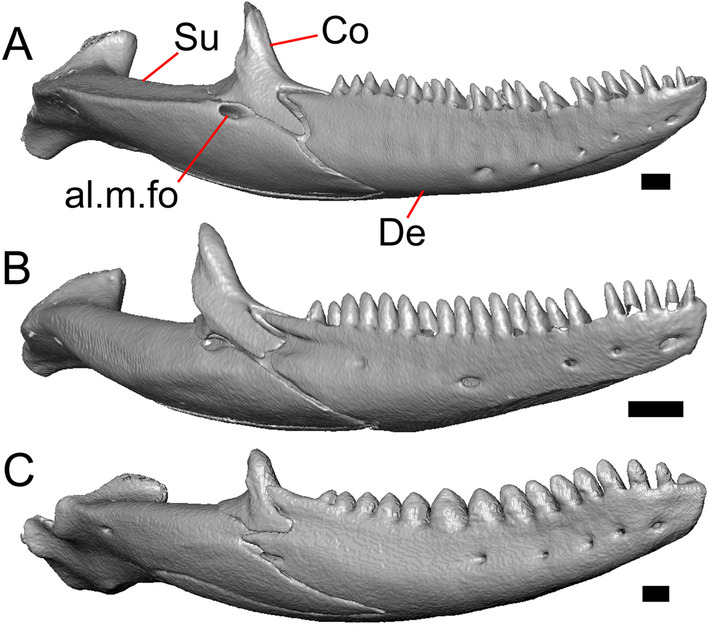
Right mandibles in anterolateral view. Scale bars = 1 mm. **a** *G. infernalis* TNHC 18988. **b** *A. campbelli* UTA 35945. **c** *P. apodus* YPM 12870. *al.m.fo* anterolateral mandibular foramen, *Co* coronoid, *De* dentary, *Su* surangular

65. Elements that contribute to the anterior margin of the anterolateral mandibular foramen (anterior surangular foramen) (modified from Conrad et al. 2011 [18], characters 172 and 173): 0 = the foramen is bordered only by the surangular (Fig. 43a); 1 = the lateral process of the coronoid contributes to the anterior margin of the foramen (Fig. 43b); 2 = the surangular process of the dentary contributes to the anterior margin of the foramen (Fig. 43c).

#### Remarks.

A coronoid contribution to the anterior border of the anterolateral mandibular foramen is present in some species of *Abronia* (e.g., *A. campbelli*, *A. mixteca*, *A. gadovii*, *A. monticola*), *B. ciliaris* FMNH 30707, *E. panamintina* MVZ 191076, *E. velazquezi* SDNHM 68677, and the right mandibles of *E. paucicarinata* SDNHM 45106 and *E. kingii* SDNHM 24252. There is a dentary contribution to the foramen in *P. apodus* YPM 12870.

66. Shape of the surangular shelf (modified from Good 1987 [12], character 82; see Ledesma et al. 2021 [14]): 0 = dorsal surface of the surangular shelf is raised and slopes to face medially and laterally (Fig. 41d); 1 = dorsal surface of the surangular shelf is flat (Fig. 41e).

#### Remarks.

A flat surangular shelf is present in *G. infernalis*, *G. ophiurus* TCWC 35604, and *B. ciliaris* FMNH 30707. The dorsal surface of the surangular is not flat in all adult specimens of *G. infernalis* [14]. The left surangular of *G. liocephalus* TCWC 8585 is slightly rounded, but the specimen is scored as state 1.

**Fig. 44:**
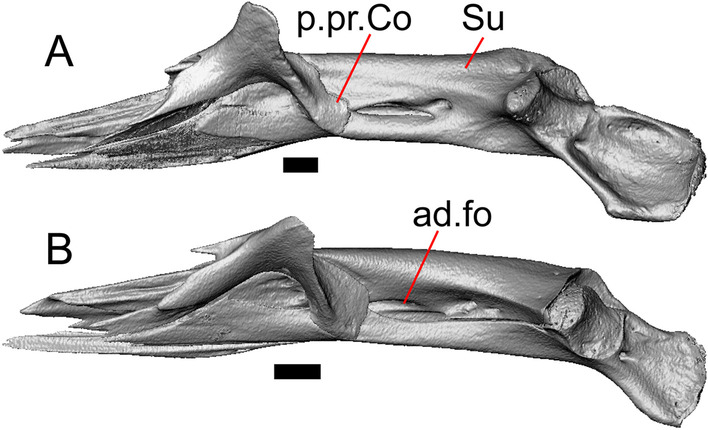
Right mandibles (excluding the dentary) in dorsomedial view. Scale bars = 1 mm. **a** *E. multicarinata* TNHC 35666. **b** *A. graminea* UTA 38831. *ad.fo* adductor fossa, *p.pr.Co* posterior process of the coronoid, *Su* surangular

67. Coronoid contribution to the adductor fossa (new): 0 = absent (Fig. 44a); 1 = present (Fig. 44b).

#### Remarks.

The posterior process of the coronoid has a small but distinct contribution to the anterior margin of the adductor fossa in *A. graminea*, *E. coerulea* TNHC 58792, and in anguines.

### Articular

68. Shape of the retroarticular process (new): 0 = spatulate, posteriorly expanded in width (Fig. 42c); 1 = subrectangular, not expanded in width posteriorly (Fig. 42d).

#### Remarks.

A relatively narrow and subrectangular retroarticular process is restricted to *A. mixteca* and *A. moreletti* TNHC 29675 among gerrhonotines, and is also present in *C. enneagrammus* FMNH 108860.

### Dentition

69. Tooth morphology of maxillary teeth (adapted from Gauthier 1982 [28]): 0 = most teeth are blunt or near bicuspid (“chisel-shaped”); 1 = heterodont; most mesial teeth are unicuspid with relatively pointed crowns, and most distal teeth are blunt to near bicuspid (Fig. 22a); 2 = most teeth are unicuspid with relatively pointed crowns (Fig. 22b); 3 = most teeth are exceedingly broad and blunt (molarized) (maxilla not pictured but see dentary on Fig. 40e).

#### Remarks.

Almost all of the maxillary teeth of *A. mixteca*, *A. graminea*, *G. parvus*, and *O. mimicus* NCSM 25699 are unicuspid and the crowns are pointed. The teeth of *B. levicollis* and *E. velazquezi* SDNHM 68677 are blunt to bicuspid. Other examined gerrhonotines exhibit heterodonty, possessing sharp and unicuspid mesial teeth and blunt, sometimes bicuspid distal teeth. All but the mesialmost teeth are molarized in *P. apodus* YPM 12870.

### Osteoderms

*General remarks on osteoderm characters.* Besides sculpturing texture and imbrication morphology, osteoderms were not extensively used for systematic purposes in gerrhonotines [12, 28]. We identified and labelled osteoderms with spatial reference to overlying scales figured by Campbell and Frost [8] and Waddick and Smith [101]. Although many cranial osteoderms are similar to overlying scales, we note that osteoderms do not always recapitulate the shape, contacts, and fusions of the overlying scales, and some scales are not represented by underlying osteoderms (e.g., the presence of a rostral osteoderm is highly variable among examined specimens). Thus, these characters should not be viewed as homologous to scale characters previously used to differentiate gerrhonotines from each other.

70. Laterally imbricating osteoderms with a distinct gliding surface (*Invariant*, Gauthier 1982 [28]): 0 = absent; 1 = present.

#### Remarks.

The presence of laterally imbricating osteoderms is an apomorphy of anguids with respect to other anguimorphs.

**Fig. 45:**
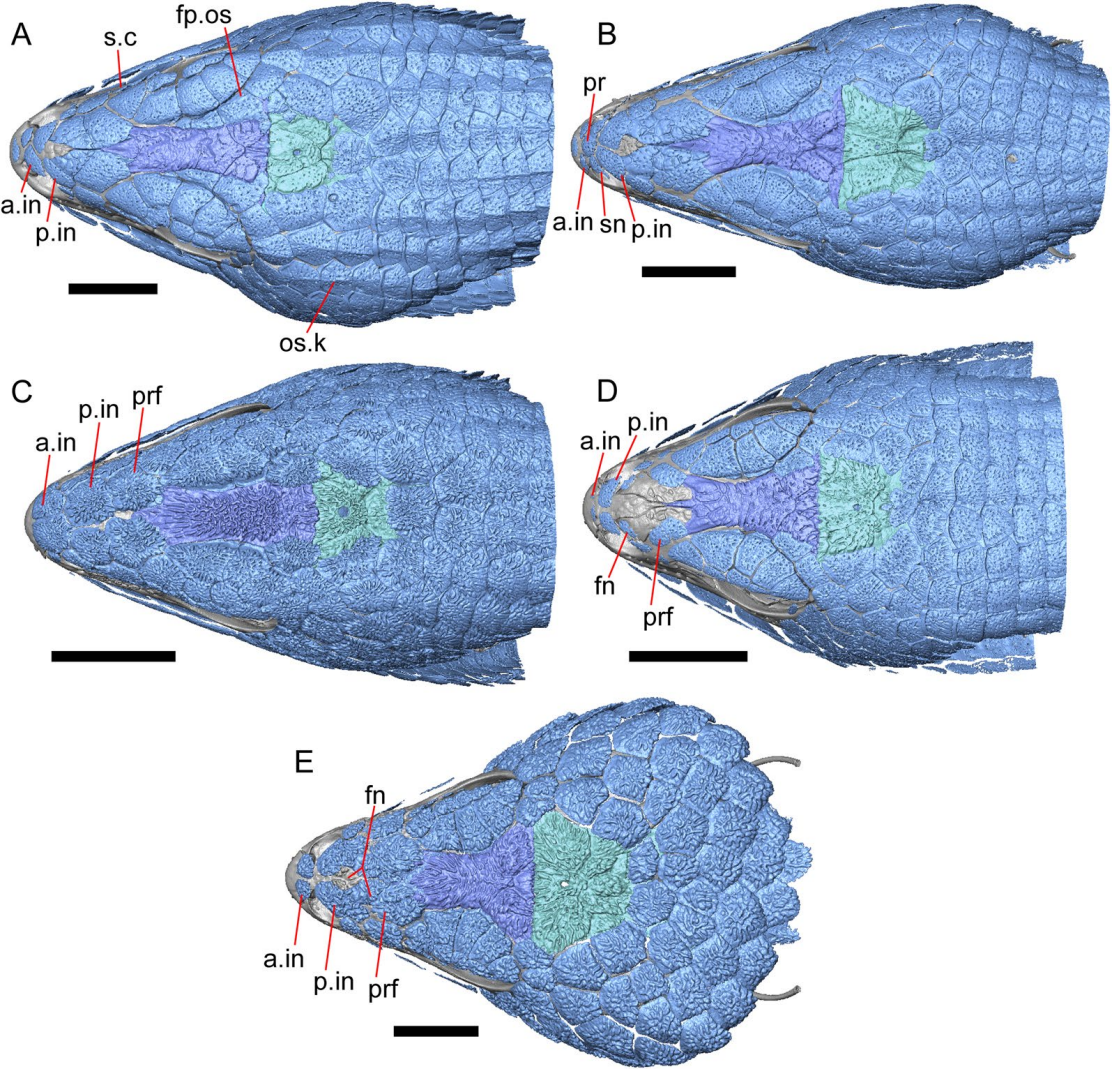
Skulls in dorsal view. Osteoderms are in light blue, frontals are in dark blue, and parietals are in teal. Scale bars = 5 mm. **a** *E. nana* SDNHM 17102. **b** *G. ophiurus* TCWC 35604. **c** *B. imbricata* TNHC 76984. **d** *A. gadovii* TCWC 9907. **e** *A. mixteca* UTA 30324. *a.in* anterior internasal osteoderm, *fn* frontonasal osteoderm(s), *fp.os* frontoparietal osteoderm, *os.k* osteoderm keel, *p.in* posterior internasal osteoderm, *pr* postrostral osteoderm, *prf* prefrontal osteoderm, *s.c* supraciliary osteoderm, *sn* supranasal osteoderm

71. Dorsal and lateral osteoderm texture (modified from Good 1987 [12], character 97; Norell, 1989 [33]): 0 = linear and pitted patterning with low to moderate relief (lightly to moderately sculptured) (Fig. 45a–c); 1 = vermiculate patterning with high relief (heavily sculptured) (Fig. 45e).

#### Remarks.

We observed lightly to moderately sculptured osteoderms in all examined specimens of *Elgaria*, *Gerrhonotus*, and *B. levicollis*, as well in *A. gadovii*, *A. monticola* TNHC 32083, and *A. moreletti* TNHC 29675 (i.e., species previously referred to *Mesaspis*). We observed vermiculate sculpturing with high relief in other species of *Abronia*. In *B. imbricata* TNHC 76984 (Fig. 45c) and *B. ciliaris* FMNH 30707, heavy sculpturing is present on some dorsal osteoderms, especially the anteriormost cranial osteoderms. The texture of the osteoderms in those species of *Barisia* is less vermiculate than in *Abronia*, especially posteriorly, and those specimens are scored as polymorphic (01). In *Abronia campbelli* UTA 35952 the sculpturing has almost no relief, but because the texture is the same as in other *Abronia*, the specimen was scored as polymorphic. Heavily sculptured osteoderms were considered a derived condition of *Abronia*, *Barisia*, and *Mesaspis* by Good [12].

72. Keels on any dorsal cranial osteoderms (osteoderms anterior to the nuchal osteoderms) (modified from Good 1988 [4]; Mead et al. 1999 [32]): 0 = absent (Fig. 45b–e); 1 = present (Fig. 45a).

#### Remarks.

The dorsal cranial osteoderms are the osteoderms anterior to the nuchal osteoderms. Keeled cranial osteoderms are present in *E. multicarinata* and *E. nana* on some but not all dorsal osteoderms, and are particularly prominent posteriorly.

**Fig. 46:**
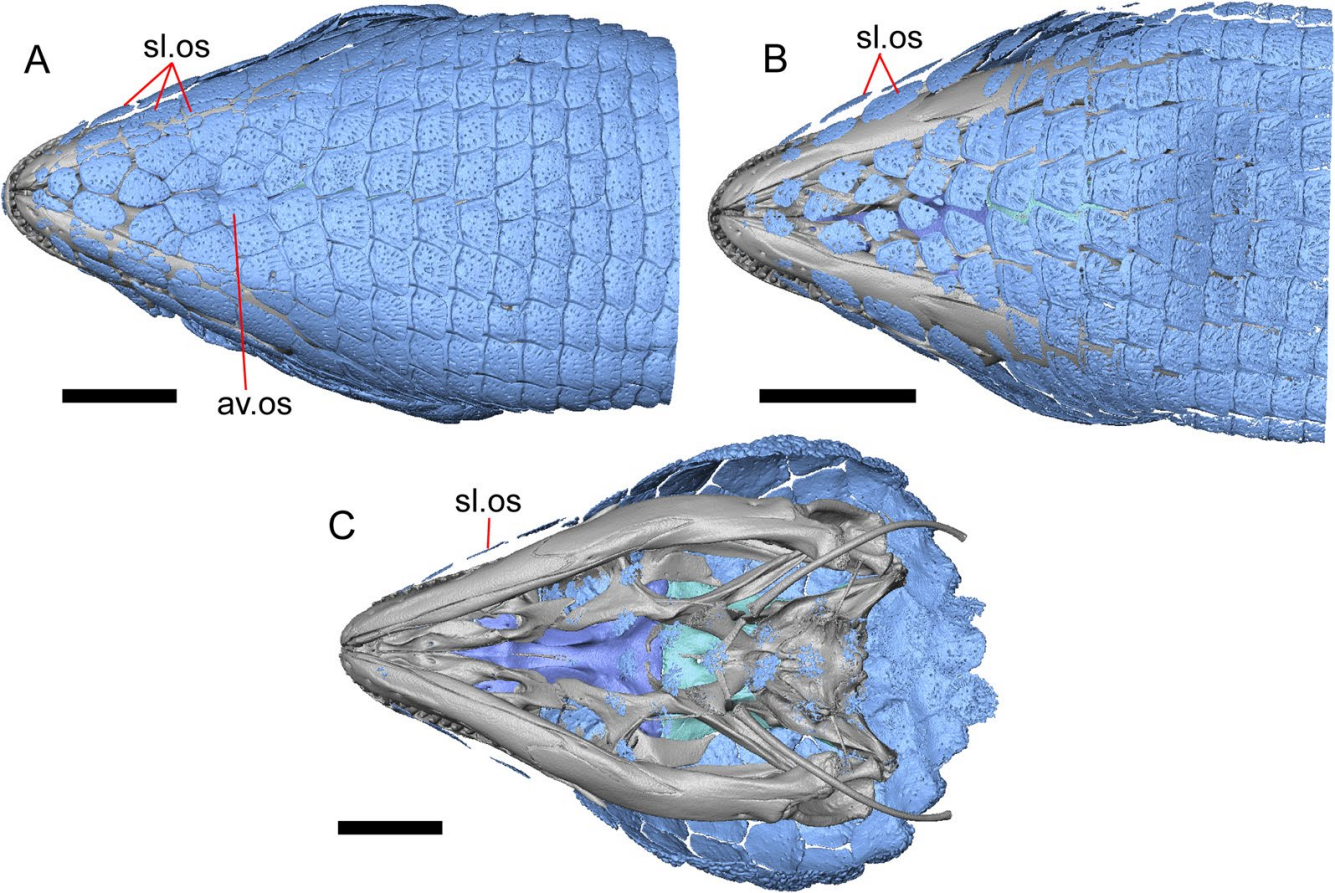
Skulls in ventral view. Osteoderms are in light blue, frontals are in dark blue, and parietals are in teal. Scale bars = 5 mm. **a** *E. nana* SDNHM 17102. **b** *A. gadovii* TCWC 9907. **c** *A. mixteca* UTA 30324. *av.os* anteroventral osteoderms, *sl.os* sublabial osteoderms

73. Anteroventral cranial osteoderms (new): 0 = well-ossified (Fig. 46a, b); 1 = poorly ossified or absent (Fig. 46c).

#### Remarks.

Well-ossified anteroventral osteoderms are present in *Barisia*, *Elgaria*, *Gerrhonotus*, *A. gadovii*, *A. moreletti* TNHC 29675, *A. monticola*, and *A. ornelasi* UTA R-6220. In *Abronia ornelasi* UTA R-6220, the anteroventral osteoderms are not as closely spaced as they are in other taxa, but they are still well-ossified, so the specimen was scored as state 0. Poorly-ossified anteroventral osteoderms are present in other examined specimens of *Abronia*, and in some specimens (e.g., *A. graminea* UTA 38831, *A. mixteca*) those osteoderms are absent. This character does not address the sublabial osteoderms (see character 74).

74. Enclosure of the dentary by the sublabial osteoderms (new, but with reference to Campbell and Frost 1993 [8] and Waddick and Smith 1974 [101]): 0 = enclosed both ventrally and laterally (three or more rows of sublabials) (Fig. 46a); 1 = enclosed laterally and unenclosed ventrally (one or two rows of sublabials) (Fig. 46b, c).

#### Remarks.

We interpret the row(s) of lateral and ventrolateral osteoderms between the infralabial region and the anteroventral osteoderms as sublabial osteoderms. In *B. levicollis*, *B. ciliaris* FMNH 30707, most *Elgaria*, most *Gerrhonotus*, and *A. gadovii* TCWC 11384, sublabial osteoderms surround the dentary both laterally and ventrally (excluding the anteriormost portion of the dentary near the ramus). In other specimens of *Abronia*, *B. imbricata* TNHC 76984, and *G. parvus* SRSU 5538, sublabials occur lateral to the dentary, but are absent ventral to the dentary. *Gerrhonotus parvus* SRSU 5537 could not be scored for this character. The ventral sublabials are almost absent in *E. coerulea* TNHC 14643 and *E. paucicarinata* SDNHM 45100, and those specimens were scored as state 1.

**Fig. 47:**
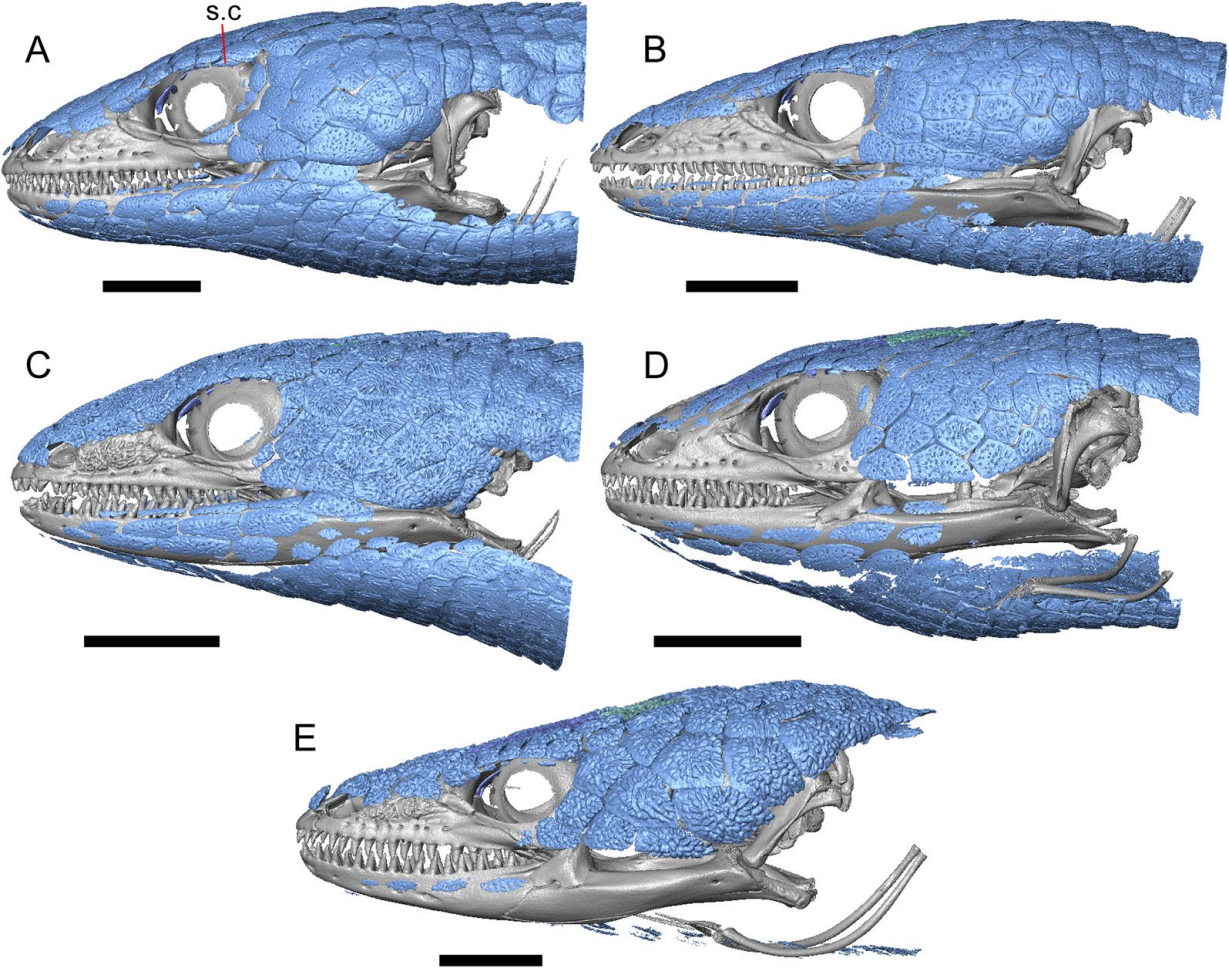
Skulls in left lateral view. Osteoderms are in light blue, frontals are in dark blue, and parietals are in teal. Scale bars = 5 mm. **a** *E. nana* SDNHM 17102. **b** *G. ophiurus* TCWC 35604. **c** *B. imbricata* TNHC 76,984. **d** *A. gadovii* TCWC 9907. **e** *A. mixteca* UTA 30324. *s.c* supraciliary osteoderm

75. Supraciliary osteoderm series (new, but with reference to Campbell and Frost 1993 [8]): 0 = spans most of the dorsal margin of the orbit (Fig. 47a–c, e); 1 = spans some of the dorsal margin of the orbit; 2 = absent (Fig. 47d).

#### Remarks.

The supraciliary osteoderms span most or all of the dorsal margin of the orbit in *Elgaria* and *Gerrhonotus*. There is a large gap between the posterior supraciliary and the median supraciliaries on the right side of the skull of *E. kingii* SDNHM 24252 and the posterior supraciliaries are absent on the left side of the skull *Elgaria nana* SDNHM 52886; those specimens are scored as polymorphic (01). In *Elgaria panamintina* MVZ 191076, the left supraciliaries are strongly developed and span the entire orbit, but there are no supraciliaries on the right side of the skull. That specimen is scored as polymorphic (02). The supraciliaries are especially well-ossified in *B. imbricata* TNHC 76984 (Fig. 47c) and *B. ciliaris* FMNH 30707. The supraciliary osteoderms may be fused to the osteoderms in the supraorbital semicircle in *Barisia levicollis*; those specimens are scored as ‘?.’

In most specimens of *Abronia*, the supraciliary osteoderms span a lesser portion of the orbit compared to *Elgaria* and *Gerrhonotus*; there are one or two anterior supraciliaries, but the posterior supraciliaries are weakly-ossified and far-spaced or are absent. The supraciliaries span most of the orbit in *A. mixteca* UTA 30324, and that specimen is scored as state 0. Supraciliary osteoderms are absent in the anguine and diploglossine outgroups and *A. gadovii* TCWC 9907.

76. Osteoderm overlying the frontoparietal shield (new): 0 = present (Fig. 45a–d); 1 = absent (Fig. 45e).

#### Remarks.

There is an osteoderm overlying some or most of each frontoparietal shield in *Elgaria*, *Gerrhonotus*, *Barisia*, *A. gadovii*, *A. monticola* TNHC 32083, and *A. moreletti* TNHC 29675. An osteoderm overlies the left side of the frontal of *A. taeniata* TCWC 30660 but there is no frontoparietal osteoderm on the other side of the skull; the specimen is scored as polymorphic. The frontoparietal osteoderm is absent in other specimens of *Abronia*. The frontoparietal shield is itself indistinct in *B. imbricata* TNHC 76984 and *A. monticola* TNHC 32083, but an osteoderm overlying the frontoparietal region is clearly present.

77. Frontonasal osteoderm region (new; inspired by Campbell and Frost 1993 [8]): 0 = absent (Fig. 45c); 1 = present (Fig. 45a, b, d–e).

#### Remarks.

In *Barisia*, the posterior internasal and prefrontal osteoderms possess medial contact along their length, excluding the presence of a frontonasal osteoderm. The presence and morphology of the frontonasal are variable among other gerrhonotines, particularly *Elgaria*, but the frontonasal region is open in all gerrhonotines besides *Barisia*. Frontonasal region morphology ranges from a relatively small, single, but distinct osteoderm in *A. graminea* and *A. taeniata*, a large, multi-osteoderm region in the keeled-scale *Gerrhonotus* and many specimens of *Elgaria*, to two reduced and separated osteoderms in several specimens of *Elgaria* and in *A. gadovii* TCWC 9907.

78. Postrostral osteoderm (new; inspired by Waddick and Smith 1974 [101]): 0 = absent (Fig. 45a, c–e); 1 = present (Fig. 45b).

#### Remarks.

A postrostral osteoderm is present in the keeled-scale *Gerrhonotus*, *B. ciliaris* FMNH 30707, and *A. gadovii* TCWC 11384.

79. Contact between the posterior internasal and the prefrontal osteoderms (new; inspired by Campbell and Frost 1993 [8]): 0 = absent (Fig. 45b, d); 1 = present on both sides (Fig. 45c, e); 2 = present on one side only due to posterior displacement of one of the posterior internasals (see below) (Fig. 45a).

#### Remarks.

Contact between the posterior internasal and the prefrontal osteoderms on both sides of the skull is present in *Abronia* (exclusive of species previously referred to *Mesaspis* and *A. ornelasi* UTA R-6220) and in *Barisia*. *Elgaria nana* SDNMH 17102 has a unique condition among examined specimens, in which the left posterior internasal is posteriorly offset from its contralateral element, and is also posteriorly elongated (or potentially fused to a piece of another osteoderm) to narrowly contact the left prefrontal osteoderm.

80. Separation of the anterior and posterior internasals by the supranasal (new; inspired by Campbell and Frost 1993 [8]): 0 = absent (Fig. 45a, c–e); 1 = present (Fig. 45b).

#### Remarks.

Separation of the internasals by the supranasal osteoderm is present in *A. campbelli*, *A. lythrochila* TNHC 112900, *A. gadovii* TCWC 11384, and the keeled-scale *Gerrhonotus*.

### Excluded characters

E1. Small foramen or foramina lateral to the medial ethmoidal foramen on the premaxilla (modified from Good 1987 [12], character 4): 0 = absent; 1 = present.

#### Remarks.

The presence of a small foramen or foramina lateral to the medial ethmoidal foramen was reported to be an apomorphy of *Barisia* [12]. There can be a variety of small openings in the bony tissue connecting the alveolar plate and the nasal process in most gerrhonotines in which a premaxillary bridge is present (character 2 of this study), not just in *Barisia* as reported by Good [12]. We did not score this character because there are multiple foramina to which this character could refer and the homology between them is not clear.

E2. Distinct posterodorsal extension of the dorsal lamina of the facial process of the maxilla (Ledesma et al. 2021 [14]): 0 = absent; 1 = present.

#### Remarks.

The posterodorsal extension of the dorsal lamina is present in many gerrhonotines. Among *Elgaria*, the extension is variably present in *E. kingii*, *E. nana*, *E. panamintina*, and *E. cedrosensis*, and is absent in *E. multicarinata*, *E. paucicarinata*, and *E. velazquezi* [14]. The extension is absent in most *Gerrhonotus*, but we observed a particularly exaggerated extension of the dorsal lamina in *G. parvus*. The extension is clearly absent in *Barisia* and several species of *Abronia* (e.g., *A. lythrochila* TNHC 112900, *A. campbelli* UTA 35952, *A. gadovii*). We chose to exclude this character because the perception of the ‘present’ or ‘absent’ states is overly influenced by the presence or absence of notches on the posterior surface of the facial process [14].

E3. Anterior–posterior (supralabial) groove on the lateral surface of the orbital process of the maxilla just ventral to the articulation with the jugal (modified from Good 1987 [12], character 18): 0 = absent; 1 = present.

#### Remarks.

A ‘supralabial’ groove on the lateral surface of the orbital process of the maxilla was described as a derived feature of *Abronia* [12] and was reported to be present in LACM 10601 [33]. We were unable to detect this morphology as illustrated by Good [12] in the two specimens of *A. mixteca* that we examined (a species also examined by Good [12]), or in any of the other *Abronia* in our sample. We did observe a shallow depression matching the character description by Norell [33] on the left maxilla of LACM 10601 (see “Description”).

E4. Sculpturing on the lateral surface of the maxilla (Conrad et al. 2011 [18], character 8): 0 = absent, 1 = present.

#### Remarks.

The absence of sculpturing on the maxilla was reported to be an unambiguous apomorphy of Anguidae exclusive of *Diploglossus bilobatus* [18]. We found that sculpturing that is distinct from fused osteoderms is present on the lateral surface of the maxilla in almost all gerrhonotines. We excluded this character because sculpturing is only absent in species with a relatively small adult body size (*G. parvus* and *A. moreletti*) and the slight sculpturing of several species of *Elgaria* (e.g., *E. coerulea* and *E. cedrosensis*) was difficult to assign to either character state.

E5. Length of the posterolateral process of the lacrimal (Ledesma et al. 2021 [14]): 0 = relatively long; 1 = relatively short.

#### Remarks.

We excluded this character because we were unable to consistently differentiate the anterior extent of the posterolateral process of the lacrimal from the rest of the element. A relatively short posterolateral process of the lacrimal, giving the entire lacrimal a short appearance, is most obvious in *A. mixteca*, *E. panamintina*, and *E. multicarinata* TNHC 35666.

E6. Bifurcation of the posterior process of the lacrimal (modified from Ledesma et al. 2021 [14]): 0 = posterior process of the lacrimal is not bifurcated; 1 = posterior process of the lacrimal is distinctly bifurcated, possessing two extended projections.

#### Remarks.

Similar to character E5, the point of distinction between the medial shelf of the lacrimal and the posterior process was not always evident. For many specimens, we were unable to determine whether the posterior process was actually bifurcated, or whether the medial shelf of the lacrimal extended relatively far posteriorly.

E7. Position of the jugal process of the postorbital relative to the postorbital process (temporal process or temporal ramus) of the jugal (Good 1987 [12], character 4): 0 = medial or posterior; 1 = anterior.

#### Remarks.

We excluded this character because the jugal-postorbital articulation is kinetic, and thus the contact is variable depending on specimen preparation [14, 19].

E8. Shape of the anterior end of the isolated frontal (new): 0 = distinctly triradiate with a large central process and two smaller lateral projections; 1 = one prominent process, with indistinct anterolateral projections.

#### Remarks.

The morphology of the anterior end of the frontal is variable in gerrhonotines. In most specimens, there is a central anteriormost process, and two smaller lateral processes that flank the nasal facets. In some specimens, the smaller lateral processes are relatively short (e.g., *A. taeniata* TCWC 4911, *E. cedrosensis* SDNHM 27702, *E. multicarinata* TNHC 4478, *E. velazquezi* SDNHM 68678) or almost absent (*E. cedrosensis* SDNHM 30296). Additionally, the entire anterior end of the frontal of *E. cedrosensis* SDNHM 30296 is marginally wider than the interorbital region of the frontal, in contrast with other specimens of *Elgaria* and other gerrhonotines (see character 32). We interpret these observed morphologies as individual variation.

E9. Ventral extent of the crista cranii (modified from Conrad et al. 2011 [18], character 67): 0 = crista cranii do not project below the dorsal-most extent of the palatine; 1 = crista cranii project below or to the dorsalmost extent of the palatine.

#### Remarks.

Contact between the palatine and the frontal (i.e., between the crista cranii and the palatine) was reported to be an unambiguous apomorphy of non-*Elgaria* gerrhonotines [18]. In skeletal specimens, the crista cranii are more likely to contact the palatine because of shrinkage of soft tissue [14]. In our CT sample, contact was present in *A. lythrochila* TNHC 112900, *A. mixteca*, *E. paucicarinata*, *E. panamintina*, *E. multicarinata* TNHC 35666, and *G. parvus* SRSU 5537. An articulation surface for the palatine is present on the crista cranii of most gerrhonotines. We hypothesize that the ventral extent of the crista cranii will generally depend on the preparation of the specimen (i.e., skeletal preparation vs. wet preparation).

E10. Orientation of the medial edge of the postparietal process proximal to where it meets the parietal table: 0 = relatively horizontal, 1 = slanted ventrally and medially (modified from Good 1987 [12], character 43; Conrad et al. 2011 [18], character 81).

#### Remarks.

Broad and flat supratemporal processes of the parietal (= postparietal processes) were reported to be an unambiguous apomorphy of the clade Anguinae + Gerrhonotinae + Glyptosaurinae [18]. We were not able to use the character as originally described or formulate a derivative character to accommodate the morphology described by Good [12] and Conrad et al. [18].

E11. Shape of the parietal table in dorsal view (modified from Good 1987 [12], character 41): 0 = trapezoidal in shape so that the anterior portion extends more laterally than does the posterior portion; 1 = square-shaped so that the anterior and posterior portions have similar widths.

#### Remarks.

A square parietal table was for the most part observed only in juvenile gerrhonotines. The parietal table is square in *A. campbelli* UTA 35945 and both specimens of *G. parvus*. This character was difficult to assess on many specimens, particularly those with substantially wider parietal tables near the articulation with the frontal (e.g., *A. mixteca* UTA 5790, *A. ornelasi* UTA R-6220). We decided to exclude this character given that difficulty and the documented ontogenetic variation in parietal morphology in anguids and in other anguimorphs [62].

E12. Notch between postparietal processes (Ledesma et al. 2021 [14]; modified from Good 1987 [12], character 43): 0 = absent; 1 = present.

#### Remarks.

In gerrhonotines, a notch can be present on the posterior end of the parietal table itself or in the middle of a posterior projection from the parietal table, but the homology between those two features is not clear. Both morphologies occur within individual species, particularly among *Elgaria*. In *E. multicarinata*, both types of notches and the absence of a notch entirely were observed [14]. Our observations appear to reflect individual variation.

E13. Morphology of the lateral margin of the postfrontal (new): 0 = lateral margin of the postfrontal is a rounded arc; 1 = lateral margin of the postfrontal possesses a distinct inflection point accompanied by a lateral projection.

#### Remarks.

The lateral projection of the postfrontal is variable in gerrhonotines, and can consist of a single projection (e.g., *E. cedrosensis* SDNHM 27702), or a blocky mass with one or more smaller lateral projections (e.g., *A. campbelli* UTA 35945, *E. kingii* SDNHM 24252). The lateral projection is almost absent in *G. lugoi* LACM 116254, a morphology that we consider to be an individual polymorphism of that specimen.

E14. Anterior mediolateral expansion of the postorbital (modified from Ledesma and Scarpetta 2018 [13]): 0 = distinct; 1 = minimal.

#### Remarks.

Mediolateral expansion of the postorbital is present in all specimens of *Elgaria* and in almost all other gerrhonotines, but is most substantial in *E. panamintina* [13]. *Elgaria velazquezi* SDNHM 68678 has a medially pointed shelf instead of the rounded shelf present in other *Elgaria*, which is similar to the morphology of *A. ornelasi* UTA R-6220 and *G. infernalis* TNHC 18988. There is no expansion in *A. graminea* UTA 38831, *A. taeniata* TCWC 4911, and *G. parvus*. The postorbital of *G. parvus* is extremely narrow across its length and there is little width differentiation between the posterior and anterior ends. In *A. taeniata* TCWC 4911, the postorbital is relatively short and narrow, but maintains the same width throughout its length except at its posteriormost end, where it tapers in width abruptly. In *A. graminea* UTA 38831, the postorbital is narrow across its length, but is slightly wider posteriorly.

E15. Contact between the squamosal and the postparietal process (Gauthier et al. 2012 [19], character 161): 0 = absent; 1 = present.

#### Remarks.

We excluded this character because of a discrepancy between data derived from CT scans and dry skeletons. We previously found that only dry skeletons of *Elgaria* displayed contact between the squamosal and postparietal process, likely because of shrinkage of soft tissue bringing the squamosal and postparietal process into contact [14].

E16. Squamosal contribution to the upper temporal fenestra (modified from Gauthier et al. 2012 [19], character 77; originally from Wu et al. 1996 [102]): 0 = the squamosal contributes broadly to the upper temporal fenestra; 1 = the squamosal has a limited contribution to the upper temporal fenestra.

#### Remarks.

The squamosal forms part of the margin of the upper temporal fenestra in all gerrhonotines examined. In *B. levicollis*, *A. gadovii*, *A. monticola* TNHC 32083, *A. moreletti* TNHC 29675, *E. kingii* SDNHM 24252, and *E. panamintina*, the squamosal is nearly excluded from the upper temporal fenestra. However, this character encompasses several morphological features (i.e., length of supratemporal, length of postorbital, length of squamosal, directionality and morphology of the postparietal processes) that each vary among gerrhonotines.

E17. Medial contact of the palatine processes of the vomers (modified from Good 1987 [12], character 26): 0 = Contacting or nearly contacting; 1 = Not close to contacting and often broadly divergent at the posteriormost ends.

#### Remarks.

The palatine processes of the vomer were reported to be divergent from one another in *Abronia* (excluding species previously placed in *Mesaspis*), although with substantial variation [12]. We also found that the palatine processes of the vomer are separated in most specimens of *Abronia*, but we observed that morphology in many other specimens that we examined (e.g., *B. imbricata* TNHC 76894, *E. kingii* SDNHM 24252, *A. gadovii* TCWC 9907, *G. infernalis* TNHC 92262).

E18. Development of an anterolaterally directed ridge that separates the vomeronasal region from the nasal region of the vomer (Ledesma et al. 2021 [14]): 0 = well-developed with high dorsal extent; 1 = poorly-developed with low dorsal extent.

#### Remarks.

Some specimens of *Abronia* and *Gerrhonotus* have a somewhat low dorsal ridge separating the vomeronasal region from the nasal region of the vomer relative to other specimens of *Gerrhonotus*, *Abronia*, and most specimens of *Elgaria*. We had difficulty defining the boundary between relatively low and relatively high for this morphological character, and so excluded it from our analyses.

E19. Dorsomedial flange on the vomerine process of the palatine (Good 1987 [12], character 33): 0 = present; 1 = absent.

#### Remarks.

The absence or reduction of a dorsomedial flange was reported to be a derived feature of *Abronia* and *Mesaspis* [12]. We were not able to identify the feature on the vomerine process of the palatine reported by Good [12].

E20. Condition of the anterior edge of the palatal plate of the pterygoid lateral to the facet for the medial pterygoid process of the palatine (new): 0 = distinct notches are present along the anterior edge; 1 = the anterior edge is smooth.

#### Remarks.

The anterior edge of the palatal plate of the pterygoid is a smooth surface uninterrupted by notches or other excavations in *A. graminea* UTA 38831. A variety of notches may interrupt the anterior edge of the palatal place in other gerrhonotines. The edge is relatively but not completely smooth in *A. mixteca*. We interterpret this feature as an individual polymorphism of *A. graminea* UTA 38831.

E21. Relative length of the paroccipital processes (see Bhullar 2012 [62]): 0 = short and projecting minimally from the otooccipital; 1 = elongate and projecting far from the otooccipital.

#### Remarks.

The paroccipital processes are short in juvenile gerrhonotines relative to adult specimens. Although we did detect variation among adult gerrhonotines (e.g., relatively short paroccipital processes in *G. parvus* SRSU 5538 compared to other adult gerrhonotines), all adult gerrhonotines have substantially longer paroccipital processes compared to those of all juvenile specimens [14].

E22. A distinct, finger-like projection of the intramandibular septum of the dentary located dorsal to the main portion of the ventral free margin of the septum (Ledesma et al. 2021 [14]): 0 = absent; 1 = present.

#### Remarks.

An additional free projection of the free margin of the intramandibular septum is present in *E. paucicarinata* SDNHM 45106 and the left dentary of SDNHM 45100, *A. lythrochila* (TNHC 112900), and *A. taeniata* TCWC 30660. In some other specimens a less separated projection was present (e.g., *G. liocephalus* TCWC 8585), and we found it difficult to differentiate a small separate projection from no projection at all.

E23. Angle of the posterior process of the coronoid with respect to the horizontal-axis of the mandible: 0 = process points more posteroventrally; 1 = process points more ventrally (Good 1987 [12], character 84).

#### Remarks.

A more ventrally directed posterior process of the coronoid was reported to diagnose *Gerrhonotus* from other gerrhonotines [12]. A ventrally directed coronoid process is clearly present in *G. lugoi* LACM 116254, a species that was not observed by Good [12]. The angle of the posterior process can be qualitatively observed or quantitatively measured from either its anterior or its posterior margin. Because the directionality of those two margins often differs, this character could not be scored consistently within many individuals (e.g., *G. infernalis* TNHC 18988).

## Supplementary Information

**Additional file 1.** Other specimens examined. Other specimens examined in this study.

**Additional file 2.** Phylogenetic matrix for alligator lizards. Morphological phylogenetic matrix formatted for Bayesian analysis in MrBayes.

**Additional file 3.** Phylogenetic matrix for alligator lizards. Morphological phylogenetic matrix formatted for parsimony analysis in PAUP*.

**Additional file 4.** Binning commands. Commands for binning continuous character ratios.

**Additional file 5. Table S1.** Continuous character measurements. Measurements (in mm) for continuous characters and relevant ratios for each character. If importing for analysis delete or skip the first row.

**Additional file 6. Table S2.** Metrics from parsimony analyses.

## Data Availability

All CT datasets are available at MorphoSource.org. All specimens scanned for this study, including those from Ledesma et al. [14], are freely available and listed in the MorphoSource project at https://www.morphosource.org/projects/000366370. For other specimens, *Abronia graminea* CAS 138886 (media ID 000040209) is available at https://www.morphosource.org/concern/media/000040209; *Barisia ciliaris* FMNH 30707 (media ID 000065102 and listed as *Barisia imbricata*) is available at https://www.morphosource.org/concern/media/000065102; *Celestus enneagrammus* FMNH 108860 (media ID 000098461) is available at https://www.morphosource.org/concern/media/000098461; *Ophisaurus mimicus* NCSM 25699 (media ID 000069149) is available at https://www.morphosource.org/concern/media/000069149; and *Pseudopus apodus* YPM 12870 (media ID 000098584) is available at https://www.morphosource.org/concern/media/000098584. A list of additional specimens examined are in Additional file 1: Figs. S1–S3. The phylogenetic matrix and associated Bayesian and parsimony analysis commands are in Additional file 2 and Additional file 3, respectively. The code used to bin continuous characters is in Additional file 4. Measurements of the continuous characters are in Additional file 5: Table S1. Parsimony analysis metrics are in Additional file 6.: Table S2.

## Abbreviations

CM: Carnegie Museum of Natural History, Pittsburgh, Pennsylvania
CAS: California Academy of Sciences, San Francisco, California
FMNH: Field Museum of Natural History, Chicago, Illinois
LACM: Natural History Museum of Los Angeles County, Los Angeles, California (formerly Los Angeles County Museum)
MVZ: Museum of Vertebrate Zoology, University of California, Berkeley, California
NCSM: North Carolina Museum of Natural Sciences Herpetology Collection, Raleigh, North Carolina
SDNHM: San Diego Museum of Natural History, San Diego, California
SRSU: Sul Ross State University, Alpine, Texas
TCWC: The Texas A&M Biodiversity Research and Teaching Collections (Texas Cooperative Wildlife Collection), College Station, Texas
TNHC: Biodiversity Collections, The University of Texas at Austin, Austin, Texas (formerly Texas Natural History Collections)
TxVP: Vertebrate Paleontology Collections, The University of Texas at Austin, Austin, Texas (formerly TMM, Texas Memorial Museum)
UF: University of Florida, Gainesville, Floridae
UTA: University of Texas at Arlington Amphibian and Reptile Diversity Research Center, Arlington, Texas
