# Supplementary material for: A new extinct species of alligator lizard (Squamata: Elgaria) and an expanded perspective on the osteology and phylogeny of Gerrhonotinae
Source: BMC Ecol Evol. 2021 Sep 29;21:184. doi: 10.1186/s12862-021-01912-8 (PMC8482661; doi:10.1186/s12862-021-01912-8)
Supplement: Supplementary file 1 — Additional file 1. Other specimens examined. Other specimens examined in this study. [file 12862_2021_1912_MOESM1_ESM.docx]

**Additional File 1**

**A new extinct species of alligator lizard (Squamata: *Elgaria*) and an expanded perspective on the osteology and phylogeny of Gerrhonotinae**

**Simon G. Scarpetta^1,2*^, David T. Ledesma^3^, Christopher J. Bell^1^**

^1^Department of Geological Sciences, Jackson School of Geosciences, The University of Texas at Austin

^2^Museum of Vertebrate Zoology, Department of Integrative Biology, University of California, Berkeley

^3^Department of Integrative Biology, The University of Texas at Austin

*Corresponding author

**Other specimens examined:**

**Xenosauridae**

*Xenosaurus grandis* FMNH 123702

*Xenosaurus platyceps* UTA 23594

*Xenosaurus grandis* TxVP M-8960

**Anguidae**

*Pseudopus apodus* TxVP M-9002

*Diploglossus pleei* CAS 200840

*Ophisaurus ventralis* CAS 74296; TxVP M-8585

*Anguis fragilis* CAS 55193; TxVP M-8963

*Ophiodes striatus* CAS 231485

**Gerrhonotinae (juveniles)**

*Abronia graminea* UTA 38834

*Barisia imbricata* TNHC 32849

*Mesaspis moreletii* TNHC 28472

**Supplemental Figures**

**Fig S1.** Strict consensus tree of the parsimony analysis in which *Gerrhonotus parvus* could attach anywhere.

**Fig S2.** Strict consensus tree of the parsimony analysis in which *Gerrhonotus parvus* was constrained to be the sister taxon of the keeled-scale *Gerrhonotus*.

**Fig S3.** Strict consensus tree of the unconstrained parsimony analysis.
